# Ab Initio Calculations on the Ground and Excited Electronic States of Thorium–Ammonia, Thorium–Aza-Crown, and Thorium–Crown Ether Complexes

**DOI:** 10.3390/molecules28124712

**Published:** 2023-06-12

**Authors:** Zhongyuan Lu, Benjamin A. Jackson, Evangelos Miliordos

**Affiliations:** Department of Chemistry and Biochemistry, Auburn University, Auburn, AL 36849-5312, USA

**Keywords:** thorium, multi-reference wave functions, solvated electrons, ammonia, crown ethers, aza-crown ethers, f-block, exited states, ab initio

## Abstract

Positively charged metal–ammonia complexes are known to host peripheral, diffuse electrons around their molecular skeleton. The resulting neutral species form materials known as expanded or liquid metals. Alkali, alkaline earth, and transition metals have been investigated previously in experimental and theoretical studies of both the gas and condensed phase. This work is the first ab initio exploration of an *f*-block metal–ammonia complex. The ground and excited states are calculated for Th^0–3+^ complexes with ammonia, crown ethers, and aza-crown ethers. For Th^3+^ complexes, the one valence electron Th populates the metal’s 6*d* or 7*f* orbitals. For Th^0–2+^, the additional electrons prefer occupation of the outer s- and p-type orbitals of the complex, except Th(NH_3_)_10_, which uniquely places all four electrons in outer orbitals of the complex. Although thorium coordinates up to ten ammonia ligands, octa-coordinated complexes are more stable. Crown ether complexes have a similar electronic spectrum to ammonia complexes, but excitations of electrons in the outer orbitals of the complex are higher in energy. Aza-crown ethers disfavor the orbitals perpendicular to the crowns, attributed to the N-H bonds pointing along the plane of the crowns.

## 1. Introduction

Isolated (gas phase) metal–ammonia complexes have been shown to host one or more diffuse electrons in their periphery [1,2]. Such complexes are called solvated electron precursors (SEPs) and the diffuse electrons reside at hydrogenic-type orbitals, which follow the energy order observed for the nuclear or Jellium shell models [1]. Specifically, the lowest-energy outer orbital is of s-type (dubbed 1s), followed by the 1p, 1d, 2s, 2p, and 1f orbitals [1]. This shell structure is universal and is independent of the central metal, including alkali metals, alkaline earth metals, boron, and transition metals [2,3,4,5]. The transition metal–ammonia complexes retain inner-valence *d*-orbitals of the metal [3,6,7,8]. Spectroscopic studies exist in the literature for lithium, sodium, magnesium, calcium, aluminum, vanadium, chromium, nickel, cobalt, copper, and silver [7,9,10,11,12,13,14,15,16,17,18,19,20]. In this work, we provide the first theoretical investigation of an *f*-block metal SEP, focusing on thorium–ammonia complexes.

Materials composed of SEPs (liquid or expanded metals) have been synthesized and characterized in detail for lithium [21,22], but have also been reported for multiple metals, including the lanthanides europium and ytterbium with the composition Eu(NH_3_)_6_ and Yb(NH_3_)_6_ [23]. Recently, such materials have been proposed as redox catalysts [24] and candidates for quantum information applications [25].

The first four ionization energies (IEs) of thorium (6.3067, 11.9, 20.0, and 28.8 eV) are remarkably lower than those of transition metals or other *f*-block elements [26]. For example, the same IE values for Eu, Yb, and V are in the ranges of 5.7–6.7, 11.3–14.6, 24.9–29.3, and 42.7–46.7 eV. Note that although the first two IEs are both comparable, the third IE of thorium is about 5 eV lower, while its fourth IE is more than 15 eV lower and of the same order of the third IE of other metals. As will be explained later, this fact in combination with the large coordination numbers for its first solvation shell (up to ten) [27,28] render ammonia ligands capable of stabilizing highly oxidized metal centers and displacing multiple electrons of thorium to the periphery of the thorium–ammonia complex.

As shown later, a Th(NH_3_)_10_ is feasible with a Th^4+^ center and four diffuse electrons. This is the largest number of diffuse electrons observed so far. However, the most stable structure, Th(NH_3_)_8_, has a Th^3+^ center and three diffuse electrons. To see if this electronic structure is characteristic of ammonia coordination only, we used two aza-crown ether ligands with four and five nitrogen atoms each, thus retaining the number of nitrogen atoms anchored to the metal. Such complexes have been studied before for lithium, sodium, and magnesium [29]. Finally, we replaced the aza-crown ethers with the corresponding crown ethers (NH groups replaced by O atoms) to observe the effect of the coordinating atom and the presence of N−H bonds in the electronic structure of the complex.

In the next section, we detail the computational methods employed currently. Then we discuss our findings, and finally summarize our results.

## 2. Computational Details

The geometry optimizations were performed at the density functional theory (DFT) level using the CAM-B3LYP functional combined with the cc-pVDZ, cc-pVDZ, aug-cc-pVDZ, and cc-pVDZ-PP basis sets for carbon, nitrogen, hydrogen, and thorium centers, respectively [30,31,32,33]. The latter basis set is supplemented with the relative pseudopotential [34]. The employed functional was shown to provide accurate geometries of the MP2 and CCSD(T) level for other metal–ammonia complexes [35]. Gaussian 16 was invoked for these calculations [36]. Every optimized structure bears real harmonic vibrational frequencies; geometries and energies are given in the Supplementary Materials (SM).

The Th(NH_3_)_8_^4+^, Th(NH_3_)_10_^4+^, Th(12C4N)_2_^4+^, Th(15C5N)_2_^4+^, Th(12C4O)_2_^4+^ crown ether structures were used for the subsequent multi-reference calculations: 12C4X/15C5X denote aza-crown ethers with 12/15 the total number of non-hydrogen atoms and 4/5 the number of nitrogen (X=N) or oxygen (X=O) atoms. These species are closed shell, and they adopt the highest possible symmetry compared to the trications, dications, monocations, or neutral counterparts.

The active space for the CASSCF (complete active space self-consistent field) calculations generally includes both inner (6*d*, 7*f*) and outer (1s, 1p, 1d) diffuse orbitals, but has been adjusted to balance the computational cost based on the population of the various orbitals. For example, the inner orbitals are less and less populated when more electrons are added. The exact active space has been optimized by multiple trial-and-error attempts for each molecular species and is provided below accordingly. The subsequent CASPT2 (CASSCF + second-order perturbation theory) [37] calculations included the dynamic correlation from the ammonia/ammine/oxygen lone pairs as well. CASPT2 calculations have been shown to be sensitive to the used active space [38]. CASPT2 calculations with similar active spaces have been shown to agree perfectly with electron propagator techniques and EOM-CCSD calculations for other metal–ammonia complexes [1,3,39]. Due to the high computational cost, only the s and p functions of nitrogen and carbon centers from the cc-pVDZ sets are included. This is expected to have a minimal effect on computed excitation energies (<0.1 eV), as excitations occur only within the Th valence space and the peripheral orbitals of the complex, which are described predominantly by the hydrogen atom basis functions [40]. The MOLPRO suite of codes [41] is used, specifically, the internally contracted version of CASPT2 (CASPT2c) [42]. A level shift value of 0.2 a.u. and IPEA shift of 0.25 a.u. were used to resolve linear dependence issues [43].

## 3. Results and Discussion

**Figure 1:**
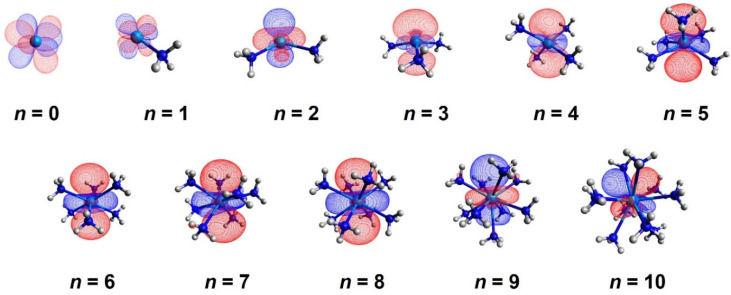
Optimized geometries and contours for the singly occupied orbital of Th(NH_3_)*_n_*^3+^, *n* = 0–10.

We first optimized the geometries for the Th(NH_3_)*_n_*^4+^ and Th(NH_3_)*_n_*^3+^ species for *n* = 1–10. We considered only the isomers where all ammonia ligands are coordinated to thorium. These systems have simple electronic structure (no or one unpaired electron) and are described properly with single determinantal methods such as DFT. The optimized geometries for trications are shown in Figure 1, along with the singly occupied molecular orbital (SOMO). for each structure. The ground state of Th^3+^ is ^2^F(7*f*^1^) and it stays in the ^2^F state only for one ammonia ligand. After coordination of additional ammonias results in the population of a ~6*d*_z_^2^-type SOMO up to *n* = 9. For Th(NH_3_)_10_^3+^, the SOMO becomes a ~6*d*_xz_ type.

**Figure 2:**
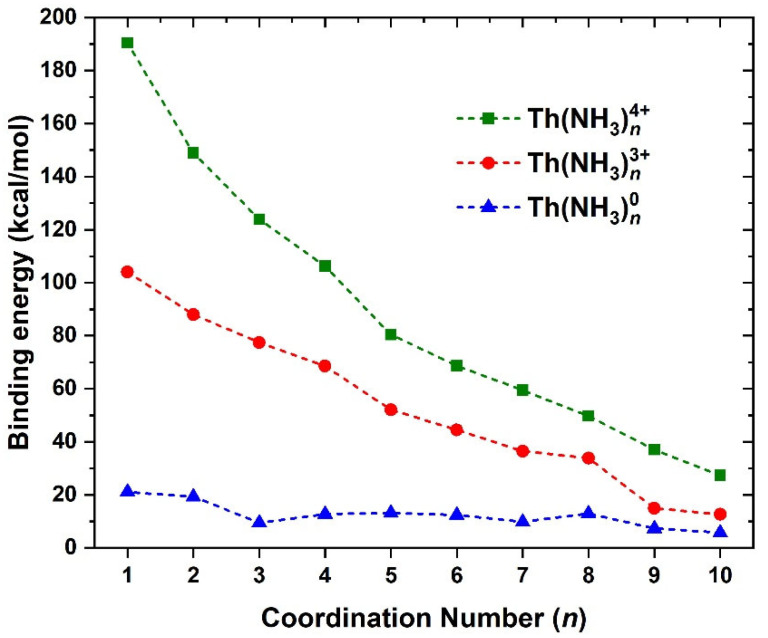
Dissociation energy (D_e_) for ammonia ligands in the Th(NH_3_)*_n_*^4+,3+,0^ complexes corresponding to the Th(NH_3_)*_n_*^4+,3+,0^ → Th(NH_3_)*_n_*_−1_^4+,3+,0^ + NH_3_ dissociation process.

The sequential dissociation energy D_e_ for the ammonia ligands, Th(NH_3_)*_n_*^4+,3+,0^ → Th(NH_3_)*_n_*_−1_^4+,3+,0^ + NH_3_ at CAM-B3LYP (D_e_ = E[Th(NH_3_)*_n_*_−1_^4+,3+,0^] + E[NH_3_] − E[Th(NH_3_)*_n_*^4+,3+,0^], where E[X] denotes the equilibrium energy of species X), is plotted with respect to *n* in Figure 2. We faced insurmountable technical/convergence issues for several cationic and dicationic species, likely due to their complex electronic structure (see below), and thus these species are not included in the figure. The ground state is a singlet and doublet for 4+ and 3+ charges, respectively, and is a singlet for 1 ≤ *n* ≤ 4 and triplet for 5 ≤ *n* ≤ 10 in the neutral complexes (see Supplementary Materials). The binding energy drops sharply with increase in *n* for the +4 charge (on average by 18 kcal/mol per ammonia ligand). For all species, but more evidently for the trications, there is a sudden drop going from *n* = 8 to *n* = 9. For the neutral, the D_e_ range is 15 ± 6 kcal/mol for 1 ≤ *n* ≤ 8, which becomes 7.3 and 5.7 kcal/mol for *n* = 9 and 10, and there is a slight increase in D_e_ from *n* = 7 to *n* = 8 (9.8 to 13.0 kcal/mol). Therefore, we believe that the most prominent structure of a thorium expanded metal will be the octacoordinated Th(NH_3_)_8_, unlike the hexacoordinate Eu(NH_3_)_6_ and Yb(NH_3_)_6_ [23].

**Figure 3:**
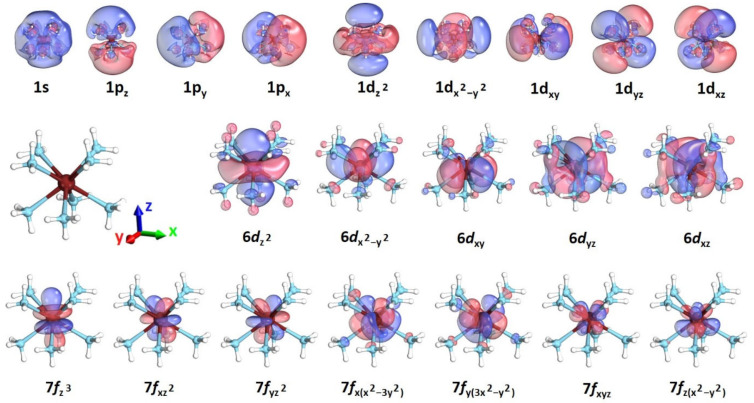
Geometry of Th(NH_3_)_8_^4+^ and CASSCF active orbitals for Th(NH_3_)_8_^3+^; 1s, 1p, 1d correspond to peripheral outer orbitals, and 6*d*, 7*f* to inner thorium orbitals.

Using the Th(NH_3_)_8_^4+^ structure (D_4d_ actual point group, C_2v_ computational point group), we performed CASSCF and CASPT2 calculations for all species with charges from 3+ to 0 in order to elucidate their electronic structure and explain the convergence issues in DFT for the intermediate charges (1+ and 2+). The geometry and CASSCF active orbitals for Th(NH_3_)_8_^3+^ are depicted in Figure 3 and include the inner 6*d* and 7*f* orbitals of thorium and the outer 1s, 1p, and 1d of the whole complex (1e^−^/21 orbitals). The energies for the 1s^1^, 1p^1^, 1d^1^, 6*d*^1^, and 7*f*^1^ states are listed in Table 1. The lowest energy states, $\tilde{X}$^2^A_1_, *1*^2^E_2_, and *1*^2^E_3_, correspond to 6*d* orbitals of thorium. The ground state has a (6*d*_z_^2^)^1^ configuration and is well separated from the other 6*d* states and higher excited states by ≥1.3 eV. The first few 7*f* states (*1*^2^B_2_ and *1*^2^E_1_) appear next at ~2.0 eV, followed closely by the first electronic state with an outer electron (*2*^2^A_1_; 1s^1^). At ~1.0 eV higher are the 1p^1^ states (*2*^2^B_2_, *2*^2^E_1_) and one more degenerate 7*f* state (*2*^2^E_2_). The last 7*f* state is at ~3.5 eV. All outer 1d states lie between 4.2 and 4.9 eV. Note that the CASSCF and CASPT2 excitation energies differ by less than 0.17 eV.

The addition of one more electron results in a highly multi-reference wave function, explaining the convergence issues with DFT. CASSCF calculations (2e^−^/15 orbitals) of the dication includes two electrons in fifteen orbitals (1s, 1p, 1d, 6*d*z^2^, and five orbitals of 7*f*/6*d* character). The results show the ground state $\tilde{X}$^1^A_1_ of Th(NH_3_)_8_^2+^ has two major electronic configurations, X˜A 11〉≈0.84 1s2〉−0.49 6dz22〉, and it is just 0.06 eV lower than the A˜A 31 state, A˜A 31〉≈0.99 1s16dz21〉. The singlet state can be seen as a mixture of a closed-shell singlet 1s^2^ (49%) and an open-shell singlet 1s^1^(6*d*_z_^2^)^1^ (51%). The percentages are estimated from the 0.84 and 0.49 coefficients [44].

The next electronic states (^1,3^B_2_, ^1,3^E_1_) lie between 0.72 and 0.93 eV and include all combinations of 1s^1^1p^1^ and (6*d*_z_^2^)^1^1p^1^. The first ^1,3^E_2_ states follow at 1.28 eV with configurations 1s^1^(*f*/*d*)^1^ and (6*d*_z_^2^)^1^1(*f*/*d*)^1^, where *f*/*d* refers to the orbitals produced from mixing 7*f*/6*d* orbital functions. We calculated ten more states, where electrons are promoted to outer 1d orbitals and Th 7*f* orbitals: 1.33 (^1^A_1_), 1.43 (^1^A_1_), 1.75 (^1^A_1_), 1.76 (^3^E_3_), 1.80 (^1^E_1_), 1.82 (^3^E_2_), 1.83 (^3^A_1_), 1.85 (^1^E_2_), 1.89 (^3^E_1_), 1.90 (^1^E_3_) eV.

Upon adding one more electron to form Th(NH_3_)_8_^1+^, the ground state $\tilde{X}$^2^A_1_ still has two major configurations involving the 1s and 6*d*_z_^2^ orbitals, X˜A 21〉≈0.84 6dz211s2〉−0.34 6dz221s1〉, comprising 71% and 12% of the wave function, respectively. Next, four quasi-degenerate states (two quartet and two doublet states) lie at 0.53 ± 0.03 eV (see Table 2). The quartets are single reference states with 95% of the wave function produced from some (6*d*_z_^2^)^1^1s^1^1p^1^ configuration. The same percentages for the doublet states are 45% (*1*^2^E_1_) and 41% (*1*^2^B_2_) after adding the contribution from all three Slater determinants with different spin-up/spin-down combinations of (6*d*_z_^2^)^1^1s^1^1p^1^. The next-largest term (34% for *1*^2^E_1_ and 37% for *1*^2^B_2_) corresponds to the 1s^2^1p^1^ configuration, which has three diffuse outer electrons, and finally a 15% portion (for both *1*^2^E_1_ and *1*^2^B_2_) belongs to (6*d*_z_^2^)^2^1p^1^, where there is only one outer diffuse electron.

The above results were obtained with a 3e^−^/10 orbital active space, with the 10 orbitals being the 6*d*_z_^2^, 1s, 1p, and 1d. According to these calculations, the higher-energy states are extremely multi-reference lying above 1.0 eV at the CASSCF level of theory. Compared to the dicationic and tricationic species, Th(NH_3_)_8_^1+^ has a higher density of low-lying electronic states with five states in the first 0.6 eV. Only one or two states are present in this energy range for Th(NH_3_)_8_^3+^ and Th(NH_3_)_8_^2+^. Finally, as noted for the trication, the CASSCF and CASPT2 excitation energies are also in perfect agreement here (within 0.04 eV).

Moving to the neutral species (4e^−^/10 orbitals), the fourth valence electron occupies a 1p orbital, resulting in the (6*d*_z_^2^)^1^1s^2^1p^1^ configuration of the $\tilde{X}$^3^E_1_ ground state, which is 72% of the wave function. This state is comparable to the addition of a 1p electron to (6*d*_z_^2^)^1^1s^2^, the major component of ground state $\tilde{X}$^2^A_1_ in Th(NH_3_)_8_^1+^. The other component of $\tilde{X}$^2^A_1_ is a (6*d*_z_^2^)^2^1s^1^; addition of a 1p electron to this configuration constitutes only 2% of the ground state for Th(NH_3_)_8_^0^. The 1p_x,y_ orbitals are populated first and the corresponding singlet and triplet states (^1,3^E_1_) are practically degenerate (see Table 2). The states *1*^1,3^B_2_ pertain to occupation of 1p_z_ and are higher by <0.1 eV.

The next batch of electronic states, *1*^3,5^A_2_ and *1*^3,5^E_3_, have a (6*d*_z_^2^)^1^1s^1^1p^2^ character by 72% (S = 1) and 92% (S = 2). In every case, the 1p^2^ electrons couple into a triplet spin state. All lie in the range between 0.30 and 0.39 eV (see Table 2). Coupling of the 1p^2^ electrons into a singlet spin multiplicity generates the largest portion (from 42% to 58%) of the last six states of Table 2. Resembling the ^1^D state of carbon, there are five 1p^2^ components belonging to the E_2_, E_3_ and A_1_ irreducible representations. The second-largest contribution to the wave function of these states (26–34%) pertains to (6*d*_z_^2^)^1^1s^2^1d^1^, which also has five components of the same irreducible representations. The excitation energies for these six states are 0.54−0.63 eV. Overall, the neutral species have the most “dense” electronic spectrum, with 14 states present within 0.63 eV. As in the cation, no 6*d* or 7*f* orbital (excluding 6*d*_z_^2^) is occupied within the states studied, as additional electrons (relative to Th^3+^) favor occupation of the outer orbitals. The active space used is the same as in Th(NH_3_)_8_^1+^ (4e^−^/10 orbitals).

**Figure 4:**
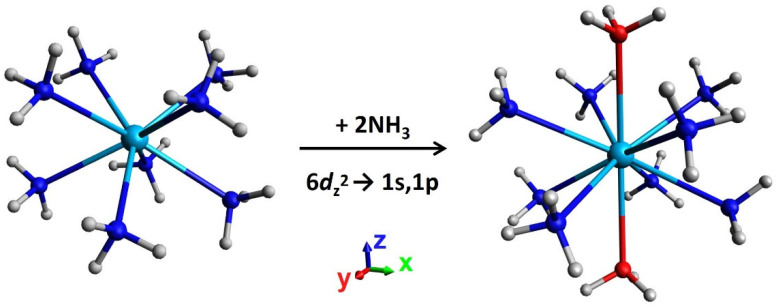
The addition of two ammonia ligands (marked with red nitrogen atoms) to the Th(NH_3_)_8_^3+,2+,1+,0^ species occurs along the z-direction, resulting in the promotion of the 6*d*_z_^2^ electron to an outer 1s or 1p orbital.

In all octacoordinated thorium complexes, there is one inner electron in 6*d*_z_^2^, which is perturbed by the molecular skeleton in order to avoid all Th-N coordination bonds (see Figure 3). This orbital has a substantial metallic/non-bonding character. The other 6*d* orbitals have some σ_Th−N_* anti-bonding character and are higher in energy by at least 1.3 eV (see Table 1). As such, the addition of further ammonia ligands is expected to destabilize the 6*d*_z_^2^ orbital, as these must approach along the z-direction, inducing a similar anti-bonding character. This is evidenced by our study of Th(NH_3_)_10_, where the addition of two ammonia ligands results in the promotion of the 6*d*_z_^2^ electron to an outer 1s or 1p orbital (see Figure 4).

Specifically, we performed multi-reference calculations for all Th(NH_3_)_10_^3+,2+,1+,0^ species (4e^−^/10 orbitals, 3e^−^/14 orbitals, 2e^−^/9 orbitals, 1e^−^/9 orbitals) and found the ground states of Th(NH_3_)_10_^1+,0^ have no inner electrons. Instead, they adopt 1s^1^1p^2^ (S = 3/2) and 1s^2^1p^2^ (S = 1) configurations. The ground states of Th(NH_3_)_10_^2+,3+^ retain a metallic electron; this electron occupies an orbital and is composed of a mixture of *6d*/*7f* orbitals in order to minimize its amplitude along the Th-N bonds. However, the *6d*/*7f* → 1s^1^ excitation for Th(NH_3_)_10_^3+^ occurs at 0.72 eV and for Th(NH_3_)_10_^2+^ at 0.14 eV compared to 2.05 eV for Th(NH_3_)_8_^3+^ These results illustrate how the decacoordinate complex destabilizes the metallic electronic states and favors the promotion of electrons to the outer orbitals. The CASSCF active space used for each system included (number of electrons/number of orbitals) 1/10, 2/14, 3/9, and 4/9 for Th(NH_3_)_10_^3+,2+,1+,0^, respectively; at CASPT2, correlation of all NH_3_ lone pairs was also included and the geometries used were of C_2_ symmetry. Further, it appears that the displacement of the 6*d*_z_^2^ electron to the periphery of the complex reduces the binding energy of the ninth and tenth ammonia ligands (see Figure 2).

**Figure 5:**
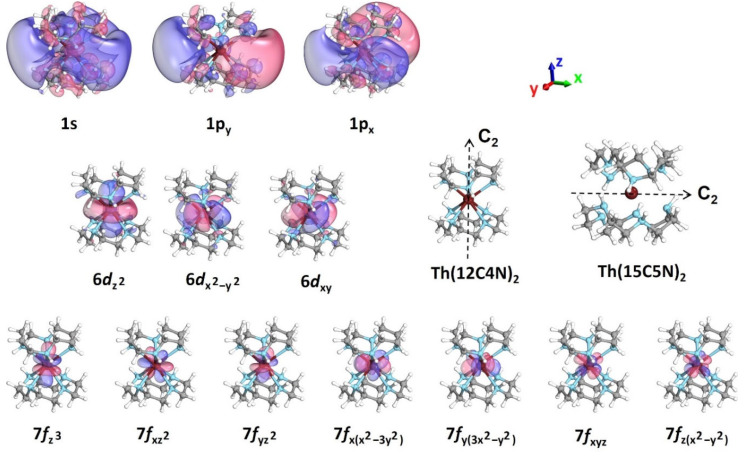
Geometries of Th(12C4N)_2_^4+^, Th(15C5N)_2_^4+^, and CASSCF active orbitals for Th(12C4N)_2_^3+^; 1s and 1p correspond to peripheral outer orbitals, and 6*d*, 7*f* to inner thorium orbitals.

In an attempt to identify more stable complexes, we then studied the Th(12C4N)_2_ and Th(15C5N)_2_ aza-crown ethers (shown in Figure 5), where the “top” and “bottom” ammonia ligands of Figure 4 are connected via a hydrocarbon bridge with two carbon atoms (tetradentate ligands). Binding energies of the aza-crown ligands were calculated in two ways: (1) by dissociating the two ligands from Th, but keeping them fixed in the optimum geometry of the complex, and (2) by optimizing the dissociated ligands. For the constrained dissociation, the binding energy per Th-N bond in Th(12C4N)_2_^4+,3+,0^ is 114.0, 68.8, and 13.7 kcal/mol, respectively. The corresponding binding energies of the unconstrained geometry are 108.9, 63.7, and 8.6 kcal/mol. The drop in binding energy illustrates a steric strain introduced to the aza-crown rings during Th coordination of the order of ~5 kcal/mol. At CAM-B3LYP, the binding energy per Th-N bond in Th(NH_3_)_8_^4+,3+,0^ is 103.5, 63.1, and 13.6 kcal/mol. From this, we see the energy of the 4+ and 3+ aza-crowns is comparable to that of the Th(NH_3_)_n_^0^ complexes. However, the polydentate binding results in a more stable complex, as the removal of one aza-crown ligand is significantly more difficult, e.g., 4 × 8.6 = 34.4 kcal/mol. A similar situation is found for the Th(15C5N)_2_^4+,3+,0^ complexes: the per Th-N bond energies are 89.2, 53.2, and 12.2 kcal/mol for Th(NH_3_)_10_^4+,3+,0^. The corresponding values for Th(15C5N)_2_^4+,3+,0^ are 93.0, 55.0, and 7.1 kcal/mol, respectively. Removal of one 15C5N ligand requires a minimum of 28.4 kcal/mol (4 × 7.1). In this case, we were able to obtain the CAM-B3LYP binding energies for all charges from 4+ to 0, which are listed in Table 3. This increased stability facilitates the formation of the neutral, decacoordinate Th complex, where it was unfavorable with ammonia ligands. This begs the question: How does the electronic structure change when replacing ammonia with aza-crown ethers, and does Th(15C5N)_2_ have four peripheral electrons like Th(NH_3_)_10_?

To answer these questions, we performed CASSCF calculations. These were performed using the Th(12C4N)_2_^4+^ and Th(15C5N)_2_^4+^ optimized geometries, as they bear the highest possible symmetry (C_2_ point group; see Figure 5 for the C_2_ axis). Under this symmetry, the 1p_x_ and 1p_y_ orbitals remain quasi-degenerate, but the 1p_z_ is destabilized considerably and is not populated in the low-lying electronic states (see Figure 5 for the z-axis). The same happens for the 1d orbitals, where only the 1d_xy_ and 1d_x_^2^_−y_^2^ orbitals participate in the low-lying states; all orbitals with amplitudes along the z-axis shift to higher energies, as has been seen for lithium–crown ether complexes [45]. Therefore, the active space has been adjusted to exclude these orbitals. Specifically, the active space used for the aza-crown ethers is (number of electrons/number of orbitals): 1/13, 2/8, 3/6, 4/6 and 1/15, 2/9, 3/6, 4/6 for Th(12C4N)_2_^3+,2+,1+,0^ and Th(15C5N)_2_^3+,2+,1+,0^, respectively.

The active orbitals and excitation energies for Th(12C4N)_2_^3+^ are given in Figure 5 and Table 1, respectively. Similarly to Th(NH_3_)_8_^3+^, the ground state retains its (6*d*_z_^2^)^1^ character and is still followed by the (6*d*_xy_)^1^/(6*d*_x_^2^_−y_^2^)^1^ states at around 1.04/1.02 eV (~0.3 eV lower). However, the inner 6*d*_xz/yz_ and outer 1p_z_ orbitals are no longer populated in the low-lying states, while the 1s outer orbital is polarized towards the xy plane. There are three reasons for these observed differences: (1) the Th^4+^ charge is less screened along the xy plane, (2) all N-H bonds (known to solvate electrons) point in the ±x/y directions, and (3) many of the C-H bonds (known to “repel” diffuse electrons) [5,25] point in the ±z directions. These effects shift the energy of the 1s^1^ state by ~0.7 eV from 2.05 to 2.76 eV. The 1p_x,y_^1^ states are practically unaffected, and among the 1*f* orbitals, only the energy of the (7*f*_x(x_^2^_−3y_^2^_)_)^1^ and (7*f*_y(3x_^2^_−y_^2^_)_)^1^ states changes considerably, shifting from 3.59 to 1.86 ± 0.02 eV.

Going from Th(12C4N)_2_^3+^ to Th(15C5N)_2_^3+^, the overall picture remains the same, but now the inner 6*d*_xz/yz_ orbitals are populated and are highly mixed with the 7*f* ones. It appears that the larger ring provides more space along the xz/yz diagonals, lowering the energy of the 6*d*_xz/yz_^1^ states. This is the reason that the active space of Th(15C5N)_2_^3+^ includes two more orbitals (see above). These nine states (seven 7*f*^1^ + two 6*d*_xz/yz_^1^) cover an energy range between 1.48 and 3.08 eV. The (6*d*_xy_)^1^/(6*d*_x_^2^_−y_^2^)^1^ states are located at 1.18/1.22 eV, and the outer 1s^1^ and 1p_x,y_^1^ states move to 3.23 and 3.44/3.52 eV (~0.4 and ~0.2 eV higher). In all the three complexes Th(NH_3_)_8_^3+^, Th(12C4N)_2_^3+^, and Th(15C5N)_2_^3+^ CASPT2 (vs. CASSCF) stabilizes the 1s^1^ and 1p^1^ states by about 0.3 eV.

The ground state of the dicationic thorium–aza-crown–ether complexes is a multi-reference singlet electronic state involving a mixture of 1s^2^, (6*d*_z_^2^)^2^, and 1s^1^(6*d*_z_^2^)^1^ configurations. This is followed by the single reference triplet 1s^1^(6*d*_z_^2^)^1^ state. These states are 0.07 and 0.20 eV apart for Th(12C4N)_2_^2+^ and Th(15C5N)_2_^2+^, respectively. This mirrors the first two states of Th(NH_3_)_8_^2+^. In Th(NH_3_)_8_^2+^, the next states are six states with 1s^1^1p^1^ and (6*d*_z_^2^)^1^1p^1^ character, but for the aza-crown ether complexes, only four of these states remain below 1.0 eV (0.28–0.38 eV for the tetra- and 0.59–0.71 eV for the penta-amine ligands), as the 1p_z_ orbital is highly destabilized.

The low-lying electronic states of the aza-crown monocationic complexes are identical to those of Th(NH_3_)_8_^+^ (see Table 2) if we exclude the ^2,4^B_2_ states, which populate the 1p_z_ orbital. The degeneracy of the ^2,4^E_1_ states is lifted due to the lower symmetry: the two components of the doublet states are at 0.02/0.26 and 0.09/0.43 eV for Th(12C4N)_2_^+^ and Th(15C5N)_2_^+^, respectively, and those of the quartet states are at 0.04/0.29 and 0.14/0.57 eV.

Finally, for the neutral aza-crown complexes, only the states corresponding to $\tilde{X}$^3^E_1_, ${\tilde{X}}^{΄}$^1^E_1_, *1*^3^A_2_, *1*^5^A_2_, *1*^1^E_2_, and *1*^3^E_2_ of Th(NH_3_)_8_ (see Table 2) “survive”, since the 1p_z_, 1d_z_^2^, and 1d_xz/yz_ outer orbitals do not contribute. There are five nearly degenerate states within 0.03/0.13 eV for Th(12C4N)_2_/Th(15C5N)_2_. These are very multi-reference states (largest coefficient 0.48) with (6*d*_z_^2^)^1^1s^2^1p_x,y_^1^ and (6*d*_z_^2^)^1^1s^1^1p_x_^1^1p_y_^1^ characters, with the ^3^B always the ground state, followed by the ^1^A (at 0.00/0.09 eV), ^1^B (at 0.01/0.10 eV), ^3^A (at 0.02/0.13 eV), and ^5^B (at 0.03/0.12 eV) states. Notice the ^5^B [(6*d*_z_^2^)^1^1s^1^1p_x_^1^1p_y_^1^] state is now closer to the ground state, since it has two electrons in the 1p orbitals, which are stabilized over 1s (see discussion on Th(NH_3_)_8_^3+^ above and Table 1). The next four states are also highly multi-reference, involving the (6*d*_z_^2^)^1^1s^1^1p^2^ and (6*d*_z_^2^)^1^1s^2^(1d_xy,x_^2^_−y_^2^)^1^ configurations, and are located at 0.74–0.77/0.24–0.35 eV higher than the ground state.

To answer our earlier questions: the replacement of ammonia ligands with aza-crown ethers has a significant impact on the electronic structure and Th(15C5N)_2_ has only three peripheral electrons, unlike Th(NH_3_)_10_, which has four. While Th(NH_3_)_10_ hosts four outer electrons (1s^2^1p^2^) in the ground state, Th(15C5N)_2_ favors the (6*d*_z_^2^)^1^1s*^m^*1p_x,y_*^n^* (*m* + *n* = 3) configurations. The main reason is that two ammonia ligands are placed along the z-axis, which disfavors the presence of a 6*d*_z_^2^ electron and promotes this electron to the outer 1s orbital (see Figure 4). The two neutral octa-coordination complexes, Th(NH_3_)_8_ and Th(12C4N)_2_, have three outer electrons (1s^2^1p^1^) and one inner 6*d*_z_^2^ electron in their ground states. For the three reasons discussed above and symmetry lowering (D_4d_ to C_2_), only the 1p_x,y_ and 1d_xy,x_^2^_−y_^2^ (unlike 1p_z_ and 1d_z_^2^,_xz,yz_) participate in the low-lying states.

Finally, we performed calculations for the crown ether Th(12C4O)_2_*^q^* and Th(15C5O)_2_*^q^* (q = +4, +3, +2, +1, 0) complexes. Such complexes are the building units of electrides and our goal is to provide insights for electrides made of oxygen- and nitrogen-based crown ethers. Our CAM-B3LYP geometries and energies are listed in the Supplementary Materials and the binding energies are reported in Table 3. The per-bond binding energies are identical for the two kinds of ethers within the accuracy of our calculations.

Regarding the excited states of Th(12C4O)_2_^3+^, the electronic structure more closely resembles Th(NH_3_)_8_^3+^ than Th(12C4N)_2_^3+^ owing to the greater equivalence of the z-direction to the x-,y-, as demonstrated by the relative energy of 1p_z_^1^ to 1p_x,y_^1^ (3.36, 3.60, 3.6 eV). The absence of the N-H bonds along the xy plane appears to make the space more isotropic. Calculated excitation energies are listed in Table 1 (1e^−^/20 orbitals). The excited 6*d* states are higher in energy than those of Th(NH_3_)_8_^3+^ and the 7*f* orbitals are highly mixed. The outer 1s and 1p states are higher than the ammonia and aza-crown complexes, but the 1d states are in the same energy range as Th(NH_3_)_8_^3+^.

The addition of an electron (CASSCF active space 2e^−^/12 orbitals) leads to two singlet states of (6*d*_z_^2^)^2^ and (6*d*_z_^2^)^1^1s^1^ mixed character at 0.00 and 0.43 eV, while the triplet (6*d*_z_^2^)^1^1s^1^ state is located in between (0.21 eV). The next six states correspond to (6*d*_z_^2^)^1^1p^1^. The three triplets have energies of 0.31, 0.43, 0.55 eV and the three singlets 0.43, 0.51, 0.56 eV. The addition of further electrons (CASSCF active spaces 3e^−^/6 orbitals, 4e^−^/6 orbitals) yields highly multi-reference wave functions. The ground state of Th(12C4O)_2_^1+^ is mainly (6*d*_z_^2^)^1^1s^2^ followed by the (6*d*_z_^2^)^1^1s^1^1p^1^ states lying between 0.04 and 0.18 eV, while the ground state of Th(12C4O)_2_ is (6*d*_z_^2^)^1^1s^2^1p^1^ followed by (6*d*_z_^2^)^1^1s^1^1p^2^ states spanning an energy range of 0.05–0.25 eV. In every case, the low-lying states have only one inner electron and up to three outer electrons, similarly to the other complexes.

## 4. Conclusions

This work is the first systematic investigation of thorium SEPs. We used high-level electronic structure methods to study the thorium–ammonia, thorium–aza-crown, and thorium–crown ether complexes. We found that the octa-coordinate complexes are more stable, suggesting that the stoichiometry of thorium expanded metals will be Th(NH_3_)_8_. In all cases, except Th(NH_3_)_10_, there is a Th^3+^ center and zero/one (1s^1^)/two (1s^2^)/three (1s^2^1p^1^) outer electrons for 3+/2+/1+/0 charged complexes. In the case of Th(NH_3_)_10_ there is a Th^4+^ center with four diffuse electrons. The nature and energetics of the outer orbitals change considerably with the ligand type. Ammonia makes isotropic structures keeping the near degeneracy of the 1p and 1d orbitals. The aza-crown ethers have N−H bonds parallel to the crowns (xy dimension) and thus disfavor orbitals lying along the z-axis. The replacement of NH with O (plain metal–crown ether complexes) restores partially the isotropic environment, but increases the energy of the 1s^1^ and 1p^1^ states of the trications. For all species, although the 1s^1^/1p^1^ states are higher in energy than 6*d*^1^/7*f*^1^ states, they are populated first when at least a second electron is added. The wave functions become extremely multi-determinantal when two or more active electrons are present, and thus multi-reference methods are sine qua non for these systems. More work on other lanthanide/actinide complexes in the gas and condensed phases is in progress.

## Figures and Tables

**Table 1:** Electronic configurations (EC) and excitation energies ΔΕ (eV) for the lowest lying electronic states of Th(NH_3_)_8_^3+^, Th(12C4N)_2_^3+^ and Th(12C4O)_2_^3+^ at the CASSCF and CASPT2 levels of theory.

State	EC	CASSCF	CASPT2	CASSCF	CASPT2	CASSCF	CASPT2
		ΔΕ[Th(NH_3_)_8_^3+^]		ΔΕ[Th(12C4N)_2_^3+^] ^a^		ΔΕ[Th(12C4O)_2_^3+^] ^a^	
$\tilde{X}$^2^A_1_	(6*d*_z_^2^)^1^	0.00	0.00	0.00	0.00	0.00	0.00
*1*^2^E_2_	(6*d*_xy_)^1^/(6*d*_x_^2^_−y_^2^)^1^	1.35	1.28	1.03/1.05	1.04/1.02	1.61/1.29	1.57/1.27
*1*^2^E_3_	(6*d*_xz_)^1^/(6*d*_yz_)^1^	1.92	1.79			4.36/4.00	4.28/3.88
*1*^2^B_2_	(7*f*_z_^3^)^1^	2.08	1.94	2.03	2.03	N/C ^b^	N/C ^b^
*1*^2^E_1_	(7*f*_xz_^2^)^1^/(7*f*_yz_^2^)^1^	2.18	2.01	2.13/2.11	2.11/2.11	N/C ^b^	N/C ^b^
*2*^2^A_1_	(1s)^1^	2.15	2.05	3.03	2.76	3.15	3.06
*2*^2^B_2_	(1p_z_)^1^	3.19	3.10			3.73	3.60
*2*^2^E_1_	(1p_x_)^1^/(1p_y_)^1^	3.24	3.15	3.50/3.36	3.23/3.10	3.42/3.71	3.36/3.64
*2*^2^E_2_	(7*f*_xyz_)^1^/(7*f*_z(x_^2^_−y_^2^_)_)^1^	3.30	3.18	3.24/3.26	3.22/3.25	N/C ^b^	N/C ^b^
*2*^2^E_3_	(7*f*_x(x_^2^_−3y_^2^_)_)^1^/(7*f*_y(3x_^2^_−y_^2^_)_)^1^	3.59	3.51	1.89/1.83	1.88/1.84	N/C ^b^	N/C ^b^
*3*^2^A_1_	(1d_z_^2^)^1^	4.24	4.20			3.95	3.82
*3*^2^E_2_	(1d_x_^2^_−y_^2^)^1^/(1d_xy_)^1^	4.46	4.40			4.29/4.26	
*3*^2^E_3_	(1d_xz_)^1^/(1d_yz_)^1^	4.89	4.86				

^a^ The degenerate states split into the ^2^A/^2^B components (C_2_ point group; see text). ^b^ The assignment was not clear, since the 7*f* orbitals are highly mixed. The CASSCF/CASPT2 energies are 1.94, 2.09, 2.35, 2.37, 2.53, 3.55, 3.72/1.93, 2.09, 2.31, 2.34, 2.49, 3.49, 3.67 eV.

**Table 2:** Electronic configurations (EC) and excitation energies ΔΕ (eV) for the lowest-lying electronic states of Th(NH_3_)_8_^1+^ and Th(NH_3_)_8_^0^ at the CASSCF and CASPT2 levels of theory.

State	Electron Configuration	ΔE (CASSCF)	ΔE (CASPT2)
Th(NH_3_)_8_^1+^			
$\tilde{X}$^2^A_1_^a^	(6*d*_z_^2^)^1^1s^2^/(6*d*_z_^2^)^2^1s^1^	0.00	0.00
*1*^2^E_1_ ^a^	(6*d*_z_^2^)^1^1s^1^1p_x,y_^1^/1s^2^1p_x,y_^1^/(6*d*_z_^2^)^2^1p_x,y_^1^	0.47	0.50
*1*^4^B_2_	(6*d*_z_^2^)^1^1s^1^1p_z_^1^	0.48	0.51
*1*^4^E_1_	(6*d*_z_^2^)^1^1s^1^1p_x,y_^1^	0.49	0.53
*1*^2^B_2_^a^	1s^2^1p_z_^1^/(6*d*_z_^2^)^2^1p_z_^1^/(6*d*_z_^2^)^1^1s^1^1p_z_^1^	0.53	0.56
**Th(NH_3_)_8_^0^**			
$\tilde{X}$^3^E_1_	(6*d*_z_^2^)^1^1s^2^1p_x,y_^1^	0.00	0.00
${\tilde{X}}^{\prime }$^1^E_1_	(6*d*_z_^2^)^1^1s^2^1p_x,y_^1^	0.01	0.00
*1*^3^B_2_	(6*d*_z_^2^)^1^1s^2^1p_z_^1^	0.04	0.04
*1*^1^B_2_	(6*d*_z_^2^)^1^1s^2^1p_z_^1^	0.11	0.09
*1*^3^A_2_	(6*d*_z_^2^)^1^1s^1^1p_x_^1^1p_y_^1^	0.28	0.30
*1*^5^A_2_	(6*d*_z_^2^)^1^1s^1^1p_x_^1^1p_y_^1^	0.27	0.31
*1*^5^E_3_	(6*d*_z_^2^)^1^1s^1^1p_z_^1^1p_x,y_^1^	0.32	0.36
*1*^3^E_3_	(6*d*_z_^2^)^1^1s^1^1p_z_^1^1p_x,y_^1^	0.38	0.39
*1*^1^E_2_^a,b^	(6*d*_z_^2^)^1^1s^1^1p^2^/(6*d*_z_^2^)^1^1s^2^(1d_xy,x_^2^_−y_^2^)^1^	0.56	0.54
*1*^3^E_2_^a,b^	(6*d*_z_^2^)^1^1s^1^1p^2^/(6*d*_z_^2^)^1^1s^2^(1d_xy,x_^2^_-y_^2^)^1^	0.55	0.56
*1*^1^E_3_^a,b^	(6*d*_z_^2^)^1^1s^1^1p^2^/(6*d*_z_^2^)^1^1s^2^(1d_xz,yz_)^1^	0.63	0.60
*2*^3^E_3_^a,b^	(6*d*_z_^2^)^1^1s^1^1p^2^/(6*d*_z_^2^)^1^1s^2^(1d_xz,yz_)^1^	0.63	0.62
*1*^3^A_1_^a,b^	(6*d*_z_^2^)^1^1s^1^1p^2^/(6*d*_z_^2^)^1^1s^2^(1d_z_^2^)^1^	0.63	0.63
*1*^1^A_1_^a,b^	(6*d*_z_^2^)^1^1s^1^1p^2^/(6*d*_z_^2^)^1^1s^2^(1d_z_^2^)^1^	0.65	0.63

^a^ See text for percentages of each configuration. ^b^ The 1p^2^ configuration is in a singlet spin multiplicity; see text for more details.

**Table 3:** CAM-B3LYP binding energies per Th-N bond for thorium ammonia, thorium aza-crown ethers, and thorium crown ethers for various charges *q*.

*q*	Th(NH_3_)_8_*^q^*	Th(12C4N)_2_*^q^*	Th(12C4O)_2_*^q^*	Th(NH_3_)_10_*^q^*	Th(15C5N)_2_*^q^*	Th(15C5O)_2_*^q^*
+4	103.5	108.9	108.0	89.2	93.0	92.9
+3	63.1	63.7	64.1	53.2	55.0	54.9
+2	N/A	38.8	38.6	34.1	36.8	36.9
+1	N/A	19.3	18.7	19.1	15.4	14.2
0	13.6	8.6	6.1	12.2	7.1	5.1

## Data Availability

DFT/CAM-B3LYP geometries and energies for all structures are listed in Tables S1–S4 of the Supplementary Materials File.

## Supplementary Materials

The following supporting information can be downloaded at: https://www.mdpi.com/article/10.3390/molecules28124712/s1, Figure S1: Structures of aza-crown ether 12C4N: (a) fully optimized, (b) fixed at the geometry in the Th(12C4N)_2_^4+^ complex.; Table S1: Energies (hartrees) for the Th(NH_3_)*_n_*_ = 0–10_^4+,3+,0^ species. The spin for the charged species is singlet (4+) and doublet (3+), while for the neutral species both the lowest singlet (S = 0) and triplet (S = 1) states are reported.; Table S2: Cartesian coordinates (Å) for the Th(NH_3_)*_n_*_ = 1–10_^4+,3+,0^ species. The spin for the charged species is singlet (4+) and doublet (3+), while for the neutral species both the lowest singlet (S = 0) and triplet (S = 1) states are reported.; Table S3: Energies (hartrees) for the Th(12C4X)_2_*^q^* and Th(15C5X)_2_*^q^* species (*q* = 4+, 3+, 2+, 1+, 0 and X = N, O). Different spin states are reported (spin is given in parenthesis).; Table S4: Cartesian coordinates (Å) for the Th(12C4X)_2_*^q^* and Th(15C5X)_2_*^q^* species (*q* = 4+, 3+, 2+, 1+, 0 and X = N, O). Different spin states are reported (spin is given in parenthesis).

## References

[1] Ariyarathna I.R., Khan S.N., Pawłowski F., Ortiz J.V., Miliordos E. (2018). Aufbau Rules for Solvated Electron Precursors: Be(NH_3_)_4_^0,±^ Complexes and Beyond. J. Phys. Chem. Lett..

[2] Ariyarathna I.R., Pawłowski F., Ortiz J.V., Miliordos E. (2018). Molecules mimicking atoms: Monomers and dimers of alkali metal solvated electron precursors. Phys. Chem. Chem. Phys..

[3] Almeida N.M.S., Pawłowski F., Ortiz J.V., Miliordos E. (2019). Transition-metal solvated-electron precursors: Diffuse and 3d electrons in V(NH_3_)^0,±^_6_. Phys. Chem. Chem. Phys..

[4] Ariyarathna I.R., Almeida N.M.S., Miliordos E. (2019). Stability and Electronic Features of Calcium Hexa-, Hepta-, and Octa-Coordinated Ammonia Complexes: A First-Principles Study. J. Phys. Chem. A.

[5] Jordan Z., Khan S.N., Jackson B.A., Miliordos E. (2022). Can boron form coordination complexes with diffuse electrons? Evidence for linked solvated electron precursors. Electron. Struct..

[6] Almeida N.M.S., Miliordos E. (2019). Electronic and structural features of octa-coordinated yttrium–ammonia complexes: The first neutral solvated electron precursor with eight ligands and three outer electrons. Phys. Chem. Chem. Phys..

[7] Jackson B.A., Miliordos E. (2021). Electronic and geometric structure of cationic and neutral chromium and molybdenum ammonia complexes. J. Chem. Phys..

[8] Khan S.N., Miliordos E. (2020). Scandium in Neutral and Positively Charged Ammonia Complexes: Balancing between Sc^2+^ and Sc^3+^. J. Phys. Chem. A.

[9] Takasu R., Misaizu F., Hashimoto K., Fuke K. (1997). Microscopic Solvation Process of Alkali Atoms in Finite Clusters:  Photoelectron and Photoionization Studies of M(NH_3_)*_n_* and M(H_2_O)*_n_* (M = Li, Li^-^, Na^-^). J. Phys. Chem. A.

[10] Brockhaus P., Hertel I.V., Schulz C.P. (1999). Electronically excited states in size-selected solvated alkali metal atoms. III. Depletion spectroscopy of Na(NH_3_)*_n_*-clusters. J. Chem. Phys..

[11] Lee J.I., Sperry D.C., Farrar J.M. (2004). Spectroscopy and reactivity of size-selected Mg^+^-ammonia clusters. J. Chem. Phys..

[12] Mune Y., Ohashi K., Iino T., Inokuchi Y., Judai K., Nishi N., Sekiya H. (2006). Infrared photodissociation spectroscopy of [Al(NH3)*_n_*]+ (*n* = 1–5): Solvation structures and insertion reactions of Al^+^ into NH_3_. Chem. Phys. Lett..

[13] Inoue K., Ohashi K., Iino T., Judai K., Nishi N., Sekiya H. (2007). Coordination and solvation of copper ion: Infrared photodissociation spectroscopy of Cu^+^(NH_3_)*_n_* (*n* = 3–8). Phys. Chem. Chem. Phys..

[14] Salter T.E., Ellis A.M. (2007). Structures of Small Li(NH3)*_n_* and Li(NH_3_)*_n_*+ Clusters (*n* = 1−5):  Evidence from Combined Photoionization Efficiency Measurements and ab Initio Calculations. J. Phys. Chem. A.

[15] Inoue K., Ohashi K., Iino T., Sasaki J., Judai K., Nishi N., Sekiya H. (2008). Coordination structures of the silver ion: Infrared photodissociation spectroscopy of Ag^+^(NH_3_)*_n_* (*n* = 3–8). Phys. Chem. Chem. Phys..

[16] Ohashi K., Inoue K., Iino T., Sasaki J., Judai K., Nishi N., Sekiya H. (2009). A molecular picture of metal ion solvation: Infrared spectroscopy of Cu^+^(NH_3_)*_n_* and Ag^+^(NH_3_)*_n_* in the gas phase. J. Mol. Liq..

[17] Imamura T., Ohashi K., Sasaki J., Inoue K., Furukawa K., Judai K., Nishi N., Sekiya H. (2010). Infrared photodissociation spectroscopy of Co^+^(NH_3_)*_n_* and Ni^+^(NH_3_)*_n_*: Preference for tetrahedral or square-planar coordination. Phys. Chem. Chem. Phys..

[18] Koga N., Ohashi K., Furukawa K., Imamura T., Judai K., Nishi N., Sekiya H. (2012). Coordination and Solvation of V^+^ with Ammonia Molecules: Infrared Photodissociation Spectroscopy of V^+^(NH_3_)*_n_* (n=4–8). Chem. Phys. Lett..

[19] Albaqami M.D., Ellis A.M. (2018). Infrared spectroscopy of Ca(NH_3_)*_n_* complexes. Chem. Phys. Lett..

[20] Kozubal J., Heck T.R., Metz R.B. (2019). Vibrational Spectroscopy of Cr^+^(NH_3_)*_n_* (*n* = 1–6) Reveals Coordination and Hydrogen-Bonding Motifs. J. Phys. Chem. A.

[21] Seel A.G., Swan H., Bowron D.T., Wasse J.C., Weller T., Edwards P.P., Howard C.A., Skipper N.T. (2017). Electron Solvation and the Unique Liquid Structure of a Mixed-Amine Expanded Metal: The Saturated Li–NH_3_–MeNH_2_ System. Angew. Chem. Int. Ed..

[22] Seel A.G., Zurek E., Ramirez-Cuesta A.J., Ryan K.R., Lodge M.T.J., Edwards P.P. (2014). Low energy structural dynamics and constrained libration of Li(NH_3_)_4_, the lowest melting point metal. Chem. Comm..

[23] Glaunsinger W.S., Von Dreele R.B., Marzke R.F., Hanson R.C., Chieux P., Damay P., Catterall R. (1984). Structures and properties of metal-ammonia compounds on the trail of a new ammonia geometry. J. Phys. Chem..

[24] Jackson B.A., Miliordos E. (2022). Simultaneous CO_2_ capture and functionalization: Solvated electron precursors as novel catalysts. Chem. Comm..

[25] Jackson B.A., Dale S.G., Camarasa-Gómez M., Miliordos E. (2023). Introducing Novel Materials with Diffuse Electrons for Applications in Redox Catalysis and Quantum Computing via Theoretical Calculations. J. Phys. Chem. C.

[26] Haynes W.M. (2012). CRC Handbook of Chemistry and Physics.

[27] Tutson C.D., Gorden A.E.V. (2017). Thorium coordination: A comprehensive review based on coordination number. Coord. Chem. Rev..

[28] Blanchard F., Rivenet M., Vigier N., Hablot I., Grandjean S., Abraham F. (2018). Solid State Chemistry of Ten-Fold Coordinate Thorium(IV) Complexes with Oxalates in the Presence of Ammonium and Hydrazinium Ions. Cryst. Growth Des..

[29] Ariyarathna I.R. (2022). Superatomic Chelates: The Cases of Metal Aza-Crown Ethers and Cryptands. Inorg. Chem..

[30] Dunning T.H. (1989). Gaussian basis sets for use in correlated molecular calculations. I. The atoms boron through neon and hydrogen. J. Chem. Phys..

[31] Kendall R.A., Dunning T.H., Harrison R.J. (1992). Electron affinities of the first-row atoms revisited. Systematic basis sets and wave functions. J. Chem. Phys..

[32] Woon D.E., Dunning T.H. (1994). Gaussian basis sets for use in correlated molecular calculations. IV. Calculation of static electrical response properties. J. Chem. Phys..

[33] Peterson K.A. (2015). Correlation consistent basis sets for actinides. I. The Th and U atoms. J. Chem. Phys..

[34] Weigand A., Cao X., Hangele T., Dolg M. (2014). Relativistic Small-Core Pseudopotentials for Actinium, Thorium, and Protactinium. J. Phys. Chem. A.

[35] Ariyarathna I.R., Pawłowski F., Ortiz J.V., Miliordos E. (2020). Aufbau Principle for Diffuse Electrons of Double-Shell Metal Ammonia Complexes: The Case of M(NH_3_)_4_@12NH_3_, M = Li, Be^+^, B^2+^. J. Phys. Chem. A.

[36] Frisch M.J., Schlegel G.W.T.H.B., Scuseria G.E., Robb M.A., Cheeseman J.R., Scalmani G., Barone V., Petersson G.A., Nakatsuji H., Li X. (2016). Gaussian 16.

[37] Ghigo G., Roos B.O., Malmqvist P.A. (2004). A Modified Definition of the Zeroth-Order Hamiltonian in Multiconfigurational Perturbation Theory (CASPT2). Chem. Phys. Lett..

[38] Azizi Z., Roos B.O., Veryazov V. (2006). How accurate is the CASPT2 method?. Phys. Chem. Chem. Phys..

[39] Ariyarathna I.R., Miliordos E. (2020). Geometric and electronic structure analysis of calcium water complexes with one and two solvation shells. Phys. Chem. Chem. Phys..

[40] Jackson B.A., Miliordos E. (2022). The nature of supermolecular bonds: Investigating hydrocarbon linked beryllium solvated electron precursors. J. Chem. Phys..

[41] Werner H.-J., Knowles P.J., Knizia G., Manby F.R., Schütz M., Celani P., Györffy W., Kats D., Korona T., Lindh R. (2015). MOLPRO, Version 2015.1, a Package of ab Initio Programs. https://www.molpro.net/.

[42] Celani P., Werner H.-J. (2000). Multireference perturbation theory for large restricted and selected active space reference wave functions. J. Chem. Phys..

[43] Roos B.O., Andersson K. (1995). Multiconfigurational Perturbation-Theory with Level Shift—The Cr_2_ Potential Revisited. Chem. Phys. Lett..

[44] Miliordos E., Ruedenberg K., Xantheas S.S. (2013). Unusual Inorganic Biradicals: A Theoretical Analysis. Angew. Chem. Int. Ed..

[45] Ariyarathna I.R., Miliordos E. (2021). Ground and excited states analysis of alkali metal ethylenediamine and crown ether complexes. Phys. Chem. Chem. Phys..

