# Supplementary material for: Ab Initio Calculations on the Ground and Excited Electronic States of Thorium–Ammonia, Thorium–Aza-Crown, and Thorium–Crown Ether Complexes
Source: Molecules. 2023 Jun 12;28(12):4712. doi: 10.3390/molecules28124712 (PMC10301992; doi:10.3390/molecules28124712)
Supplement: Supplementary file 1 [file molecules-28-04712-s001.zip › molecules-2445835-supplementary.pdf]

# Supplementary Materials

# Ab Initio Calculations on the Ground and Excited Electronic States of Thorium–Ammonia, Thorium–Aza-Crown, and Thorium–Crown Ether Complexes

*Zhongyuan Lu, Benjamin A. Jackson, and Evangelos Miliordos**

Department of Chemistry and Biochemistry, Auburn University, Auburn, AL 36849-5312, USA

**Corresponding Author**

* E-mail: emiliord@auburn.edu

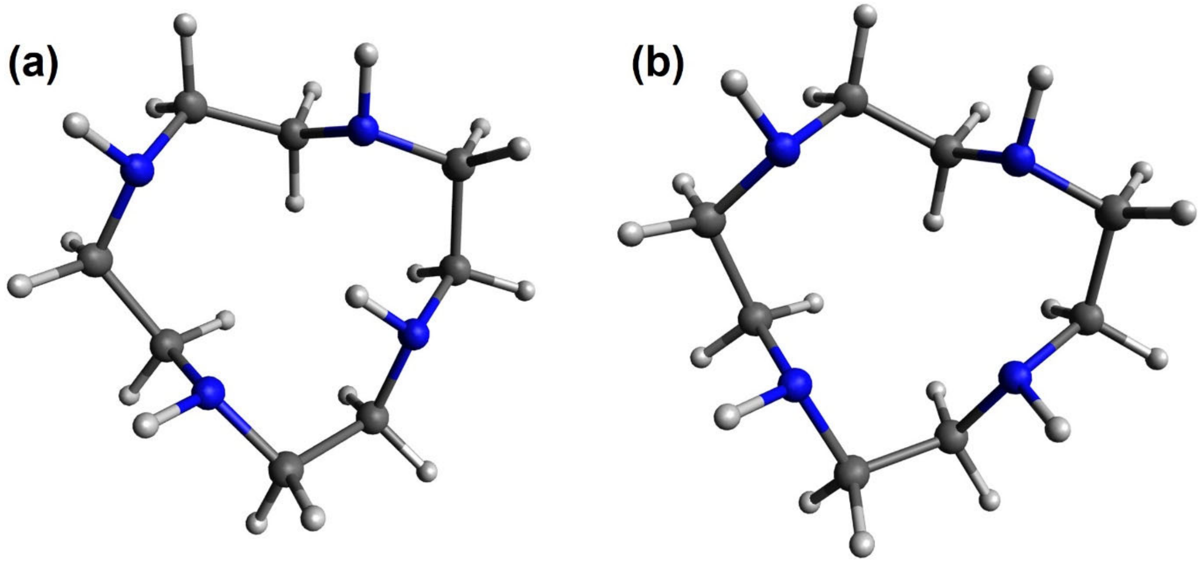


**Figure S1.** Structures of aza-crown ether 12C4N: (a) fully optimized, (b) fixed at the geometry in the $Th(12C4N)_2^{4+}$ complex.

**Table S1.** Energies (hartrees) for the $Th(NH_3)_{n=0\text{-}10}^{4+,3+,0}$ species. The spin for the charged species is singlet (4+) and doublet (3+), while for the neutral species both the lowest singlet (S=0) and triplet (S=1) states are reported.

| $n$ | $Th(NH_3)_n^{4+}$ (S=0) | $Th(NH_3)_n^{3+}$ (S=$^1/_2$) | $Th(NH_3)_n^0$ (S=0) | $Th(NH_3)_n^0$ (S=1) |
|---|---|---|---|---|
| 0 | -404.214983 | -405.278100 | -406.593310 | -406.598549 |
| 1 | -461.053438 | -461.978898 | -463.157366 | -463.164141 |
| 2 | -517.825621 | -518.654077 | -519.723392 | -519.729842 |
| 3 | -574.558086 | -575.312384 | -576.278371 | -576.279880 |
| 4 | -631.262347 | -631.956535 | -632.834821 | -632.835097 |
| 5 | -687.925485 | -688.574423 | -689.390959 | -689.386922 |
| 6 | -744.569805 | -745.180261 | -745.945630 | -745.942632 |
| 7 | -801.199465 | -801.773312 | -802.496153 | -802.495036 |
| 8 | -857.813602 | -858.362174 | -859.051770 | -859.048942 |
| 9 | -914.408325 | 914.921105 | -915.598359 | -915.597735 |
| 10 | -970.985977 | -971.475997 | -972.142435 | -972.139865 |

**Table S2.** Cartesian coordinates (Å) for the $Th(NH_3)_{n=1\text{-}10}^{4+,3+,0}$ species. The spin for the charged species is singlet (4+) and doublet (3+), while for the neutral species both the lowest singlet (S=0) and triplet (S=1) states are reported.

| *n* | $Th(NH_3)_n^{4+}$ (S=0) | $Th(NH_3)_n^{3+}$ (S=$^1/_2$) | $Th(NH_3)_n^0$ (S=0) | $Th(NH_3)_n^0$ (S=1) |
|---|---|---|---|---|
| 1 | Th -0.246876 0.000000 0.000001 | Th -0.263568 -0.000000 0.000001 | Th -0.270210 -0.000102 -0.000002 | Th -0.269575 0.000000 -0.000002 |
| | N 2.075202 -0.000000 0.000013 | N 2.234263 -0.000002 0.000008 | N 2.316789 -0.001188 -0.000020 | N 2.311247 0.000001 -0.000032 |
| | H 2.564039 -0.831207 -0.476032 | H 2.693713 -0.807113 -0.477132 | H 2.711028 -0.484307 0.815544 | H 2.694129 0.799161 0.517534 |
| | H 2.564038 0.827812 -0.481914 | H 2.693681 0.816750 -0.460468 | H 2.711161 -0.456442 -0.831385 | H 2.694136 -0.847677 0.433519 |
| | H 2.564381 0.003396 0.957757 | H 2.693886 -0.009613 0.937493 | H 2.679200 0.958214 0.016170 | H 2.694720 0.048502 -0.950670 |
| 2 | Th -0.000258 -0.258676 -0.000001 | Th -0.000220 -0.290728 0.000000 | Th 0.000011 0.016628 -0.000934 | Th 0.000000 0.000001 -0.000026 |
| | N 1.976349 1.085714 -0.000003 | N 2.033464 1.229136 -0.000001 | N 2.626899 -0.067883 0.005247 | N -2.633334 0.000015 0.000115 |
| | H 2.119755 1.740301 0.820415 | H 2.142533 1.862525 0.817007 | H 3.054150 0.510270 0.736241 | H -3.016983 0.858923 -0.407924 |
| | H 2.891363 0.550187 -0.000765 | H 2.944615 0.727013 -0.013389 | H 2.950778 -1.030919 0.151641 | H -3.016581 -0.782972 -0.539763 |
| | H 2.119109 1.741447 -0.819603 | H 2.129078 1.881437 -0.803668 | H 3.019055 0.245221 -0.888737 | H -3.016810 -0.076204 0.948027 |
| | N -1.973644 1.088013 0.000001 | N -2.031416 1.230687 -0.000001 | N -2.626935 -0.069011 0.001981 | N 2.633331 -0.000029 0.000116 |
| | H -2.634481 0.943557 0.816886 | H -2.683498 1.096373 0.799392 | H -2.978038 -0.271478 0.944374 | H 3.016829 0.076179 -0.947792 |
| | H -2.630859 0.949442 -0.820825 | H -2.664257 1.123763 -0.818739 | H -2.986305 -0.803364 -0.618322 | H 3.017000 -0.858911 0.408185 |
| | H -1.860609 2.139776 0.003971 | H -1.862996 2.255631 0.019392 | H -3.060345 0.812034 -0.291771 | H 3.016549 0.782975 0.539987 |
| 3 | Th 0.000388 -0.000652 -0.216373 | Th -0.000046 -0.000055 -0.145074 | Th 0.143217 -0.021051 -0.006042 | Th 0.000260 -0.000010 -0.050023 |
| | N -1.754317 -1.488519 0.605929 | N 0.740449 2.348277 0.404796 | N -1.944694 1.657573 0.009342 | N 2.612630 -0.022591 0.146594 |
| | H -2.479080 -1.073075 1.241774 | H 1.213064 2.863917 -0.362487 | H -1.739230 2.398313 -0.665153 | H 3.008663 -0.847686 -0.320382 |
| | H -2.354221 -1.924724 -0.140335 | H -0.010866 3.003473 0.691583 | H -2.898946 1.338882 -0.187803 | H 3.022858 0.795452 -0.320532 |
| | H -1.450637 -2.332881 1.150997 | H 1.416784 2.422870 1.187928 | H -1.959938 2.098650 0.935136 | H 2.944048 -0.025369 1.116501 |
| | N -0.417611 2.262281 0.604266 | N 1.663802 -1.814968 0.404870 | N 2.759969 0.051462 0.029188 | N -1.327005 -2.250171 0.146403 |
| | H -1.253762 2.402139 1.223457 | H 2.609948 -1.491869 0.681389 | H 3.145357 0.656643 -0.702492 | H -0.823355 -3.015565 -0.318571 |
| | H -0.586744 2.983146 -0.143368 | H 1.866331 -2.488740 -0.358637 | H 3.060989 -0.908646 -0.186575 | H -2.238602 -2.180600 -0.322487 |
| | H 0.343379 2.717617 1.166088 | H 1.395038 -2.430994 1.195200 | H 3.194113 0.328509 0.913512 | H -1.497268 -2.534212 1.116415 |
| | N 2.168841 -0.768349 0.606863 | N -2.403864 -0.532897 0.405035 | N -2.060324 -1.514364 0.014991 | N -1.287795 2.272847 0.146395 |
| | H 2.183454 -1.661271 1.158879 | H -3.090464 -0.358132 -0.353891 | H -2.999702 -1.139093 0.181763 | H -0.771525 3.029122 -0.319662 |
| | H 2.882140 -0.970982 -0.139608 | H -2.600224 -1.516833 0.668484 | H -2.087020 -2.033416 -0.870189 | H -2.200999 2.218737 -0.321399 |
| | H 2.702195 -0.119154 1.236314 | H -2.798151 -0.001597 1.204175 | H -1.889836 -2.207918 0.750944 | H -1.452010 2.560427 1.116408 |
| 4 | Th -0.000884 -0.000257 0.000055 | Th 0.000057 -0.000014 -0.000001 | Th 0.000017 -0.000050 0.000480 | Th -0.000000 -0.000021 -0.000031 |
| | N -0.031027 2.420062 -0.575221 | N 1.689186 1.497461 -1.217624 | N 2.149776 -1.050478 -1.007585 | N 2.188170 -1.584823 -0.000107 |
| | H 0.270033 3.078913 0.177882 | H 1.269577 2.203276 -1.847069 | H 2.739476 -0.352608 -1.490334 | H 3.114565 -1.137537 0.000243 |
| | H 0.575971 2.715498 -1.372840 | H 2.322605 2.059714 -0.622800 | H 2.738891 -1.503048 -0.289186 | H 2.163503 -2.198493 0.819451 |
| | H -0.956216 2.819741 -0.851388 | H 2.340315 0.996193 -1.846282 | H 1.928965 -1.772736 -1.699391 | H 2.163977 -2.198210 -0.819887 |
| | N 1.350047 -0.342651 2.061110 | N -1.497109 1.688906 1.218290 | N 2.151692 1.049673 1.005631 | N 2.188058 1.584953 0.000408 |
| | H 1.995345 0.434919 2.327395 | H -0.995692 2.339757 1.847115 | H 2.743936 0.351434 1.484716 | H 3.114473 1.137710 0.000199 |
| | H 1.994902 -1.164930 2.061318 | H -2.202944 1.269211 1.847657 | H 1.931170 1.769299 1.700282 | H 2.163678 2.198278 0.820231 |
| | H 0.815806 -0.489980 2.947015 | H -2.059297 2.322604 0.623702 | H 2.738028 1.505661 0.287173 | H 2.163515 2.198682 -0.819108 |
| | N 1.016161 -1.283838 -1.872064 | N 1.496968 -1.689720 1.217518 | N -2.151957 -1.008407 1.046456 | N -2.188100 -1.584889 -0.000340 |
| | H 1.075105 -0.792695 -2.792568 | H 2.058729 -2.323518 0.622635 | H -2.737731 -1.493942 0.347144 | H -2.163993 -2.198074 -0.820273 |
| | H 2.002563 -1.600808 -1.735871 | H 2.203167 -1.270375 1.846709 | H -1.931522 -1.698561 1.770427 | H -2.163579 -2.198751 0.819074 |
| | H 0.539674 -2.175597 -2.136010 | H 0.995516 -2.340484 1.846402 | H -2.744774 -0.291218 1.495936 | H -3.114391 -1.137399 0.000092 |
| | N -2.327832 -0.791375 0.385741 | N -1.689525 -1.496541 -1.218165 | N -2.149644 1.009645 -1.048578 | N -2.188129 1.584897 0.000282 |
| | H -2.473176 -1.423417 1.205172 | H -2.322966 -2.058998 -0.623559 | H -2.739039 1.491060 -0.349431 | H -2.163712 2.197987 0.820283 |

| | | | | |
|---|---|---|---|---|
| | H -3.046973 -0.052596 0.555787 | H -2.340645 -0.994810 -1.846460 | H -1.928653 1.703194 -1.769120 | H -2.163369 2.198859 -0.819052 |
| | H -2.764921 -1.341320 -0.387830 | H -1.270122 -2.202090 -1.848047 | H -2.739333 0.292897 -1.502874 | H -3.114644 1.137853 -0.000165 |
| 5 | Th -0.000026 0.001009 0.088091 | Th -0.002553 -0.019804 0.004700 | Th 0.126675 -0.064040 0.000075 | Th -0.003024 0.008773 -0.017559 |
| | N 2.368037 -0.039535 -0.838832 | N 1.216075 -1.842972 -1.383392 | N 2.121160 1.607237 -0.000600 | N -2.548425 -0.184727 0.863817 |
| | H 2.472083 -0.057923 -1.872911 | H 1.877044 -1.456405 -2.076109 | H 2.716661 1.455387 0.832901 | H -2.484227 -0.064508 1.881420 |
| | H 2.973044 0.763583 -0.573993 | H 1.780096 -2.544991 -0.878001 | H 2.716225 1.455502 -0.834429 | H -3.199016 0.524529 0.512398 |
| | H 2.953925 -0.847418 -0.546514 | H 0.591917 -2.419741 -1.969730 | H 1.882997 2.602647 -0.000450 | H -3.010132 -1.084715 0.708822 |
| | N -0.041099 -2.359945 -0.856474 | N -2.406450 0.561884 -0.871981 | N -1.929135 -0.808488 1.475291 | N 2.159824 -1.500566 0.564431 |
| | H 0.752017 -2.972623 -0.579234 | H -2.948728 -0.246077 -1.215126 | H -1.778724 -0.529928 2.451630 | H 1.985582 -2.509216 0.530566 |
| | H -0.038654 -2.456235 -1.891461 | H -2.403319 1.189077 -1.691043 | H -2.038967 -1.829813 1.496029 | H 2.434966 -1.300190 1.534159 |
| | H -0.859245 -2.941954 -0.586003 | H -3.060552 1.020627 -0.218569 | H -2.864891 -0.439359 1.210558 | H 2.981152 -1.334224 -0.029531 |
| | N 0.040291 2.375348 -0.822248 | N 2.492126 0.485075 0.654130 | N 2.441197 -1.293390 0.000470 | N -1.026916 2.479062 -0.405228 |
| | H -0.752406 2.984052 -0.535141 | H 2.593884 1.094560 1.479728 | H 3.013345 -1.075562 -0.830715 | H -0.995354 2.688385 -1.409414 |
| | H 0.858372 2.953493 -0.543333 | H 3.051954 -0.339662 0.919005 | H 3.013004 -1.074085 0.831539 | H -1.995628 2.637694 -0.112314 |
| | H 0.037193 2.486989 -1.855718 | H 3.079139 0.946196 -0.058623 | H 2.314142 -2.309532 0.001383 | H -0.472134 3.195964 0.077037 |
| | N -2.369891 0.041786 -0.834160 | N 0.068662 2.587139 0.040102 | N -1.928158 -0.806178 -1.477570 | N 2.179011 1.588976 -0.026326 |
| | H -2.985261 -0.740645 -0.533261 | H -0.841607 3.069790 0.009374 | H -1.776700 -0.527099 -2.453596 | H 2.804986 1.190851 -0.740507 |
| | H -2.943554 0.869590 -0.575678 | H 0.512201 2.995318 0.877286 | H -2.038309 -1.827465 -1.499058 | H 2.035841 2.565854 -0.302277 |
| | H -2.476793 0.017940 -1.867836 | H 0.585518 3.018408 -0.742734 | H -2.864100 -0.436949 -1.213602 | H 2.695689 1.616089 0.861461 |
| | N 0.002862 -0.025826 2.611311 | N -1.351975 -1.630036 1.522910 | N -1.774634 1.854982 0.001707 | N -0.755175 -2.459152 -0.838276 |
| | H -0.786787 0.467185 3.078251 | H -1.982163 -1.143768 2.180924 | H -1.731255 2.472972 -0.815496 | H -0.712033 -2.387114 -1.860792 |
| | H -0.032800 -0.964152 3.061864 | H -1.966388 -2.327560 1.073065 | H -2.743359 1.479012 -0.000739 | H -0.147303 -3.238623 -0.567832 |
| | H 0.829832 0.404492 3.075635 | H -0.768290 -2.201054 2.155128 | H -1.733799 2.468773 0.822216 | H -1.708464 -2.755465 -0.611831 |
| 6 | Th -0.000013 -0.000029 -0.000347 | Th 0.018040 0.001947 -0.000218 | Th 0.000001 -0.000004 -0.009662 | Th -0.000006 0.073224 -0.000679 |
| | N 1.588979 -1.989948 -0.467301 | N 0.147328 -2.387804 1.132320 | N -1.985326 0.825842 1.559190 | N -1.990991 -0.323115 -1.779169 |
| | N -1.574319 -1.742458 -1.089737 | N -2.162865 0.898228 1.209868 | N 1.985328 -0.826665 1.558777 | N 1.857540 -1.591112 -1.046226 |
| | N 1.579645 1.410102 -1.489570 | N -1.923185 -1.335530 -1.209722 | N 2.022404 1.773215 -0.121628 | N -1.857638 -1.578300 1.064708 |
| | N 1.577220 0.592775 1.964733 | N 2.207131 -1.034388 -1.065734 | N -2.079570 0.933777 -1.393579 | N -2.137851 1.572532 0.721480 |
| | N -1.585319 -0.077321 2.044736 | N 1.937046 1.477237 1.067187 | N -2.022438 -1.773139 -0.122520 | N 2.139132 1.563210 -0.738375 |
| | N -1.586100 1.807102 -0.960019 | N -0.360437 2.365568 -1.132103 | N 2.079597 -0.933013 -1.394038 | N 1.989845 -0.302435 1.783659 |
| | H 1.686545 -2.283164 -1.456864 | H -0.650021 -2.603299 1.747615 | H -1.822494 0.564327 2.537896 | H -1.772101 -1.082053 -2.431828 |
| | H -2.575032 1.751388 -0.655239 | H -0.467734 3.193705 -0.529078 | H 2.117853 -1.959130 -1.406790 | H 2.960513 -0.453295 1.480483 |
| | H 1.349661 -2.883252 0.002121 | H 0.950358 -2.489082 1.769648 | H -2.938041 0.474358 1.329830 | H -1.951655 0.540745 -2.330933 |
| | H 2.576978 -1.858521 -0.183434 | H 0.213825 -3.220324 0.529437 | H -2.073257 1.849935 1.562000 | H -2.962391 -0.463994 -1.473572 |
| | H -1.680424 1.840869 -1.991862 | H -1.187280 2.409168 -1.744727 | H 3.021382 -0.628275 -1.075908 | H 1.767387 -1.048783 2.449444 |
| | H -1.347651 2.790035 -0.730673 | H 0.401540 2.632550 -1.771978 | H 2.012690 -0.652487 -2.379555 | H 1.954663 0.570943 2.320547 |
| | H -1.328397 -2.038829 -2.052702 | H -2.949929 1.234603 0.637078 | H 2.937997 -0.474913 1.329651 | H 1.996229 -2.434395 -0.479730 |
| | H -1.343303 -0.764928 2.782473 | H 2.559834 0.946952 1.693379 | H -2.198511 -2.101152 -1.078522 | H 1.597442 2.164306 -1.371089 |
| | H -2.563125 -1.453262 -1.202192 | H -1.977447 1.685759 1.847502 | H 2.073396 -1.850746 1.560962 | H 2.796604 -1.215763 -1.247894 |
| | H -1.670101 -2.651081 -0.599872 | H -2.601882 0.208078 1.835895 | H 1.822434 -0.565765 2.537635 | H 1.490439 -1.918333 -1.946570 |
| | H -2.573047 -0.318859 1.844614 | H 1.591993 2.226919 1.683518 | H -2.937717 -1.417062 0.211547 | H 2.901049 1.173349 -1.303661 |
| | H -1.684104 0.799809 2.588442 | H 2.590405 1.967242 0.439286 | H -1.839151 -2.614884 0.430940 | H 2.563437 2.171665 -0.034704 |
| | H 1.338114 1.452025 -2.497305 | H -2.620440 -1.831834 -0.636821 | H 2.937681 1.417107 0.212414 | H -1.995675 -2.428531 0.508513 |
| | H 1.332185 1.444001 2.504177 | H 2.704053 -0.384640 -1.691933 | H -3.021363 0.628807 -1.075698 | H -1.594575 2.182466 1.344317 |
| | H 2.568846 1.102645 -1.520538 | H -1.575085 -2.065238 -1.848112 | H 1.838937 2.614708 0.432158 | H -2.796992 -1.200552 1.260595 |
| | H 2.565653 0.777177 1.713757 | H 2.029197 -1.840500 -1.681766 | H -2.012598 0.653888 -2.379271 | H -2.897770 1.189984 1.294374 |
| | H 1.673522 2.413420 -1.246108 | H -2.499072 -0.753963 -1.835056 | H 2.198572 2.101653 -1.077468 | H -1.491621 -1.894462 1.969439 |
| | H 1.674086 -0.118677 2.712521 | H 2.948941 -1.374476 -0.436943 | H -2.117866 1.959900 -1.405671 | H -2.564668 2.171058 0.010829 |
| 7 | Th -0.022171 0.000240 -0.000021 | Th 0.006280 0.059886 -0.023644 | Th 0.002895 0.087356 -0.059320 | Th -0.036544 0.011472 -0.006761 |
| | N -0.696122 -2.031413 -1.515128 | N 2.154809 -0.299844 -1.599381 | N 2.214017 -0.415994 -1.486839 | N -2.078715 -0.105188 1.749799 |
| | N 1.649140 -1.883548 0.778437 | N 1.823202 -1.066488 1.593875 | N 1.862873 -1.043363 1.510779 | N -2.115291 -1.395324 -0.930772 |
| | N -0.656439 -0.317397 2.524758 | N -0.171684 -2.611878 -0.404934 | N -0.216816 -2.583054 -0.325284 | N 0.947149 -2.521953 -0.077124 |
| | N -0.704406 2.339776 -0.968981 | N -1.460263 2.285601 0.306976 | N -1.461799 2.301784 0.323442 | N 1.192289 2.433781 -0.014345 |

| | | | | |
|---|---|---|---|---|
| | N 1.593280 0.260856 -2.066210 | N 1.626169 2.177575 0.310968 | N 1.802522 2.009864 0.341291 | N -1.960619 1.667912 -0.811376 |
| | N 1.645015 1.638020 1.218606 | N -1.890816 -0.875725 1.626712 | N -1.912665 -0.881968 1.565365 | N 2.212601 -0.116139 -1.468177 |
| | H 0.078118 -2.570565 -1.935561 | H 3.087862 -0.384918 -1.175470 | H 3.127898 -0.301632 -1.025543 | H -2.911622 0.470299 1.527570 |
| | H 2.285729 2.177337 0.613251 | H -2.356110 -0.116739 2.141163 | H -2.495072 -0.166906 2.019838 | H 2.537377 0.780009 -1.850857 |
| | H -1.276490 -1.805944 -2.341244 | H 2.262805 0.474767 -2.267351 | H 2.169557 0.292017 -2.232090 | H -1.741458 0.246125 2.651869 |
| | H -1.251701 -2.773013 -1.055231 | H 2.100397 -1.115498 -2.221877 | H 2.259702 -1.324526 -1.964845 | H -2.450072 -1.045386 1.933417 |
| | H 2.301007 1.211598 1.893590 | H -1.538265 -1.471151 2.386729 | H -1.492935 -1.408881 2.346217 | H 2.029280 -0.698822 -2.292650 |
| | H 1.203715 2.382170 1.785291 | H -2.672096 -1.421728 1.240767 | H -2.584149 -1.543251 1.135200 | H 3.050123 -0.519551 -1.004858 |
| | H 2.299386 -1.637189 1.542778 | H 1.433283 -1.541172 2.417909 | H 1.499379 -1.348053 2.423052 | H -2.074204 -1.397846 -1.957150 |
| | H 1.128868 0.371329 -2.983714 | H 1.577678 2.895206 -0.425583 | H 1.909369 2.497143 -0.558494 | H -1.837827 2.646382 -0.527260 |
| | H 2.295865 -2.251102 0.061153 | H 2.443861 -0.365806 2.019662 | H 2.627693 -0.379371 1.729721 | H -3.045007 -1.011010 -0.684372 |
| | H 1.209391 -2.747901 1.137816 | H 2.473751 -1.759573 1.201562 | H 2.349284 -1.861884 1.122838 | H -2.148519 -2.380564 -0.641462 |
| | H 2.243161 -0.522431 -2.244972 | H 2.630726 1.967554 0.360525 | H 2.746012 1.661012 0.590172 | H -2.920121 1.420783 -0.504023 |
| | H 2.236375 1.069542 -2.049900 | H 1.454220 2.708026 1.174456 | H 1.591272 2.735742 1.035574 | H -1.987402 1.666593 -1.839496 |
| | H -1.219424 -1.156374 2.746842 | H 0.469063 -2.992404 -1.113003 | H 0.430242 -3.015413 -0.998234 | H 0.510744 -3.170650 0.582622 |
| | H 0.067680 2.970630 -1.238810 | H -1.248552 2.806311 1.167599 | H -1.049838 2.958543 0.990686 | H 1.172866 2.878688 -0.939857 |
| | H -1.219543 0.440346 2.947727 | H -1.091486 -2.934168 -0.730695 | H -1.160895 -2.868431 -0.633401 | H 1.954016 -2.551537 0.133294 |
| | H -1.266775 2.941415 -0.342953 | H -2.476802 2.145625 0.360373 | H -2.435930 2.173899 0.614250 | H 2.185963 2.382477 0.243671 |
| | H 0.128325 -0.401187 3.191123 | H 0.005466 -3.206555 0.414825 | H -0.075594 -3.103372 0.554374 | H 0.851146 -2.942191 -1.010296 |
| | H -1.280066 2.322323 -1.828312 | H -1.363662 2.995462 -0.432334 | H -1.471934 2.779325 -0.586090 | H 0.768895 3.094395 0.641877 |
| | N -2.641823 -0.008241 0.028775 | N -2.135555 -0.128877 -1.634242 | N -2.325002 -0.121823 -1.402930 | N 2.101344 -0.063448 1.620482 |
| | H -3.106716 0.107545 -0.887477 | H -2.212655 0.685539 -2.257614 | H -2.296530 0.509674 -2.209503 | H 1.920702 0.514009 2.450987 |
| | H -3.089661 -0.866088 0.392824 | H -2.087615 -0.908322 -2.302118 | H -2.443924 -1.065204 -1.792100 | H 2.333039 -0.997268 1.984404 |
| | H -3.092411 0.729570 0.595873 | H -3.078079 -0.212772 -1.231338 | H -3.206066 0.059450 -0.903564 | H 2.999730 0.285140 1.231658 |
| 8 | Th 0.000099 -0.000061 0.000261 | Th 0.000081 0.000197 -0.000600 | Th 0.000086 -0.007166 -0.000266 | Th -0.001418 -0.001915 0.001915 |
| | N 1.683751 1.443269 1.476705 | N 2.103132 1.063317 1.359260 | N 2.262682 0.867201 1.136073 | N -0.252036 -2.355750 -1.232081 |
| | N -1.443166 1.683161 1.477440 | N -1.066145 2.102296 1.358160 | N -0.724516 2.201809 1.342644 | N -2.355934 0.264797 -1.226419 |
| | N -1.683096 -1.444182 1.476661 | N -2.102646 -1.064088 1.359826 | N -2.155591 -0.870729 1.317104 | N 0.254247 2.347701 -1.238883 |
| | N 0.163437 2.211169 -1.477390 | N 0.567987 2.288552 -1.356335 | N 0.718985 2.205289 -1.340398 | N -1.980136 -1.313979 1.224737 |
| | N -0.164013 -2.209653 -1.480022 | N -0.564727 -2.287303 -1.360028 | N -0.994174 -2.180406 -1.228612 | N 1.974287 1.303811 1.237190 |
| | N 2.211875 -0.163497 -1.476731 | N 2.287930 -0.569223 -1.359321 | N 2.158233 -0.865258 -1.317242 | N 1.326149 -1.967740 1.227476 |
| | N 1.442754 -1.684226 1.476972 | N 1.061366 -2.100317 1.364143 | N 0.998186 -2.178812 1.228476 | N 2.347193 -0.239712 -1.244823 |
| | N -2.212317 0.164542 -1.475903 | N -2.287578 0.564977 -1.360890 | N -2.264493 0.863373 -1.135977 | N -1.300908 1.976857 1.236615 |
| | H 1.640947 2.465668 1.349181 | H 2.638226 1.826245 0.927052 | H 2.829284 1.512736 0.565578 | H -0.711454 -3.090758 -0.662621 |
| | H 1.574625 1.348112 2.498613 | H 1.843331 1.438119 2.278828 | H 2.123751 1.357477 2.030004 | H -0.800247 -2.320402 -2.099505 |
| | H 2.687613 1.246152 1.347301 | H 2.829458 0.375333 1.588824 | H 2.913908 0.089842 1.355613 | H 0.653594 -2.769514 -1.500266 |
| | H -2.465226 1.643864 1.346269 | H -1.821077 2.644273 0.920503 | H -1.293837 2.869667 0.801205 | H -3.087003 0.729464 -0.655433 |
| | H -1.351999 1.570503 2.499342 | H -1.452340 1.840565 2.272428 | H -1.263359 2.030887 2.203560 | H -2.321031 0.809750 -2.095805 |
| | H -1.242601 2.686866 1.352137 | H -0.376249 2.823266 1.598653 | H 0.093152 2.757657 1.642766 | H -2.774106 -0.639849 -1.491204 |
| | H -1.642698 -2.466160 1.345057 | H -2.629933 -1.835236 0.932688 | H -2.904022 -1.244359 0.698016 | H 0.761083 3.070416 -0.693516 |
| | H -1.571295 -1.353244 2.498647 | H -1.844214 -1.428210 2.284059 | H -2.001765 -1.609119 2.015551 | H 0.757346 2.298877 -2.132531 |
| | H -2.686868 -1.244439 1.350629 | H -2.835423 -0.379565 1.579182 | H -2.620273 -0.111665 1.837333 | H -0.651811 2.786022 -1.463211 |
| | H 2.465494 -1.637168 1.353899 | H 1.834931 -2.627561 0.941371 | H 1.049906 -3.044280 0.672420 | H 3.076840 -0.728469 -0.693271 |
| | H 1.343054 -1.579450 2.498908 | H 1.420959 -1.839773 2.289565 | H 1.978687 -2.006148 1.506453 | H 2.303147 -0.755857 -2.131205 |
| | H 1.249815 -2.688316 1.343001 | H 0.376704 -2.833412 1.581850 | H 0.518585 -2.435199 2.098276 | H 2.770904 0.669883 -1.482233 |
| | H 1.011474 2.783695 -1.349220 | H 1.177935 2.987626 -0.915377 | H 1.284348 2.874894 -0.797031 | H -2.426824 -2.051257 0.648034 |
| | H 0.152568 2.066684 -2.499276 | H 1.007567 2.122141 -2.268822 | H 1.260434 2.037172 -2.200189 | H -1.703031 -1.794486 2.088780 |
| | H -0.591281 2.902002 -1.348905 | H -0.266594 2.834085 -1.600310 | H -0.100285 2.757986 -1.641821 | H -2.759879 -0.696510 1.497072 |
| | H -1.009789 -2.784593 -1.347801 | H -1.190676 -2.978885 -0.929876 | H -1.970747 -2.004756 -1.517899 | H 2.385429 2.083174 0.689797 |
| | H -0.159075 -2.064096 -2.501792 | H -0.985052 -2.117653 -2.280953 | H -0.506676 -2.444781 -2.091617 | H 1.700992 1.730325 2.130168 |
| | H 0.593300 -2.898514 -1.356388 | H 0.268480 -2.842336 -1.586630 | H -1.056339 -3.042142 -0.667710 | H 2.777602 0.698048 1.462658 |
| | H 2.786789 -1.009359 -1.344944 | H 2.973207 -1.204172 -0.932296 | H 2.906183 -1.239935 -0.698154 | H 2.076082 -2.403716 0.658230 |
| | H 2.067496 -0.157379 -2.498655 | H 2.117494 -0.980491 -2.284176 | H 2.006467 -1.601378 -2.018496 | H 1.793535 -1.683492 2.096355 |

| | | | | |
|---|---|---|---|---|
| | H 2.900565 0.593687 -1.351384 | H 2.850200 0.261450 -1.577239 | H 2.622818 -0.103857 -1.834143 | H 0.716720 -2.755599 1.494273 |
| | H -2.784155 1.012996 -1.347465 | H -2.973139 1.200445 -0.935108 | H -2.829558 1.511539 -0.566925 | H -2.062761 2.404218 0.677359 |
| | H -2.068622 0.153569 -2.497892 | H -2.116726 0.974752 -2.286347 | H -2.127038 1.349917 -2.032156 | H -1.749796 1.701416 2.117894 |
| | H -2.903648 -0.589638 -1.346901 | H -2.849614 -0.266192 -1.577608 | H -2.916560 0.085489 -1.351186 | H -0.687747 2.768786 1.481186 |
| 9 | Th 0.000000 0.000020 0.000088 | Th -0.006306 0.006816 -0.077266 | Th -0.013250 0.019830 -0.106458 | Th -0.012230 0.012286 -0.117556 |
| | N -0.084406 -2.551244 0.906178 | N 2.161089 0.353799 -1.743603 | N -2.538601 -0.039076 -1.079353 | N 0.154774 -2.192815 -1.676122 |
| | N 1.925334 -1.463030 -1.212184 | N 2.083001 -1.620808 0.778805 | N -1.796535 1.142702 1.578772 | N -1.401513 -2.065867 0.931703 |
| | N 1.972488 1.772780 -0.531337 | N -1.558289 -1.994029 1.073753 | N 1.517187 1.866574 1.156059 | N -1.873373 1.387994 1.284910 |
| | N -1.904389 -1.381226 -1.334794 | N 1.723287 1.930667 0.929656 | N -1.303974 -2.155494 0.855737 | N 2.044568 -1.576015 0.637383 |
| | N -1.858736 1.852450 -0.654992 | N -1.953920 1.486378 1.237080 | N 2.149801 -1.547527 0.264307 | N 1.538744 1.905782 1.054547 |
| | N -1.984415 -0.350570 1.804578 | N -0.444245 2.424839 -1.341592 | N 0.026038 -2.065814 -1.837401 | N 2.275584 0.508074 -1.487940 |
| | N -0.013639 2.059958 1.758723 | N -2.399699 -0.230021 -1.439332 | N 2.124981 0.811350 -1.556665 | N -0.463412 2.440799 -1.260503 |
| | N 0.096656 0.492198 -2.661124 | N 0.290440 -0.128031 2.817868 | N 0.591289 -0.630885 2.565376 | N 0.320788 -0.326823 2.682014 |
| | H 0.704453 -3.159013 0.647417 | H 3.116555 0.262909 -1.378786 | H -3.198480 -0.700565 -0.645908 | H 0.316997 -3.082492 -1.181777 |
| | H -0.107360 -2.645496 1.931075 | H 2.132675 -0.297331 -2.534573 | H -3.023353 0.873111 -0.994667 | H -0.695343 -2.335506 -2.232119 |
| | H -0.894456 -3.115222 0.616019 | H 2.172764 1.266413 -2.210033 | H -2.546224 -0.244677 -2.085810 | H 0.909295 -2.117236 -2.366903 |
| | H 2.437826 -0.999333 -1.974336 | H 1.790621 -2.523377 1.169506 | H -1.426488 1.728134 2.339965 | H -2.070277 -1.777110 1.663261 |
| | H 2.690349 -1.800935 -0.611784 | H 2.741999 -1.889136 0.040145 | H -2.487528 1.749434 1.096262 | H -1.976755 -2.583054 0.251918 |
| | H 1.600180 -2.329180 -1.666177 | H 2.692467 -1.237990 1.509361 | H -2.386450 0.440871 2.053396 | H -0.810301 -2.788448 1.374830 |
| | H 1.687213 2.598351 -1.078152 | H -2.076302 -1.736684 1.920756 | H 2.444306 1.455019 1.371682 | H -1.537910 2.047753 2.003280 |
| | H 2.431610 2.201959 0.283243 | H -2.290733 -2.349974 0.450310 | H 1.749552 2.704281 0.604597 | H -2.512440 1.950822 0.695901 |
| | H 2.774978 1.416791 -1.069125 | H -1.057395 -2.843517 1.357108 | H 1.173218 2.225206 2.055996 | H -2.519480 0.757722 1.794321 |
| | H -0.831342 2.129987 2.379197 | H -2.438506 0.434736 -2.218181 | H 2.310963 0.185132 -2.349479 | H 0.175786 2.613257 -2.041036 |
| | H 0.767309 2.100341 2.427295 | H -2.517279 -1.131216 -1.913723 | H 2.029661 1.740805 -1.983095 | H -1.401918 2.468714 -1.674357 |
| | H 0.016778 2.994937 1.328934 | H -3.296027 -0.101975 -0.954677 | H 3.012483 0.848151 -1.024123 | H -0.416241 3.268739 -0.657092 |
| | H -2.725437 -1.674469 -0.787194 | H 2.064484 2.589085 0.221452 | H -1.953789 -2.614879 0.202690 | H 2.583544 -1.996744 -0.132880 |
| | H -2.338730 -0.902692 -2.135362 | H 1.351541 2.542177 1.664926 | H -0.647582 -2.906560 1.142478 | H 2.755304 -1.045038 1.175826 |
| | H -1.591183 -2.267548 -1.757084 | H 2.588432 1.565598 1.342733 | H -1.874031 -1.977921 1.698008 | H 1.786969 -2.374356 1.239561 |
| | H -1.505155 2.663757 -1.182943 | H -2.516121 0.986022 1.934164 | H 2.887929 -1.059036 0.804753 | H 1.140317 2.392202 1.869574 |
| | H -2.640021 1.528968 -1.242242 | H -1.612750 2.305262 1.753066 | H 1.953807 -2.416230 0.790903 | H 2.415644 1.475445 1.406603 |
| | H -2.349695 2.302797 0.129012 | H -2.661310 1.884279 0.610108 | H 2.635632 -1.860288 -0.587873 | H 1.874191 2.655172 0.433593 |
| | H -2.760219 0.325285 1.769589 | H -1.429113 2.640489 -1.524759 | H 0.684621 -1.903301 -2.605092 | H 2.288124 1.398414 -2.001180 |
| | H -2.480544 -1.251131 1.767133 | H -0.089236 3.298020 -0.935647 | H 0.267144 -2.981935 -1.438751 | H 3.121419 0.535849 -0.893844 |
| | H -1.694236 -0.304797 2.792341 | H -0.038077 2.399088 -2.282279 | H -0.884577 -2.188219 -2.291864 | H 2.474114 -0.203742 -2.202288 |
| | H 0.099100 -0.347700 -3.256380 | H 1.185573 0.206307 3.189859 | H -0.073896 -0.304091 3.276289 | H 0.296994 -1.316887 2.959135 |
| | H -0.679817 1.039230 -3.056520 | H -0.394242 0.402479 3.367020 | H 0.636206 -1.651944 2.703761 | H 1.229442 0.013110 3.021875 |
| | H 0.918698 1.006978 -3.004197 | H 0.217826 -1.073388 3.208392 | H 1.511303 -0.290677 2.874407 | H -0.381575 0.127731 3.276519 |
| | N 1.851227 -0.431546 1.924296 | N 0.155746 -2.280186 -1.616123 | N -0.664616 2.474457 -1.043086 | N -2.496829 -0.172086 -1.185611 |
| | H 1.504393 -0.365294 2.892442 | H -0.218646 -2.049350 -2.542229 | H -1.623902 2.781613 -0.827211 | H -2.540188 -0.757134 -2.028988 |
| | H 2.658281 0.207573 1.933464 | H -0.350312 -3.130139 -1.343532 | H -0.649355 2.300054 -2.054911 | H -2.874728 0.741833 -1.482478 |
| | H 2.306188 -1.354351 1.921052 | H 1.096790 -2.630532 -1.822117 | H -0.067622 3.289804 -0.871398 | H -3.225615 -0.538015 -0.553874 |
| 10 | Th 0.000165 -0.000397 -0.004258 | Th 0.000262 0.000026 -0.043516 | Th 0.000024 -0.000032 -0.009355 | Th 0.000370 0.001124 0.000152 |
| | N 1.217173 2.267658 -0.946978 | N 1.810341 -1.852360 1.095451 | N -1.159529 -2.311986 -0.720063 | N -1.385421 -1.813482 -1.394948 |
| | N 1.071924 -1.133195 -2.254037 | N 1.046711 -1.207810 -2.384248 | N -1.001144 1.119569 -2.232892 | N -1.438780 1.583682 -1.599389 |
| | N -1.217160 -2.337474 -0.760423 | N -1.045162 1.235960 -2.368104 | N 1.161782 2.313247 -0.712778 | N 0.971532 2.471015 -0.270975 |
| | N 1.225788 0.965975 2.242935 | N -1.160511 -1.015697 2.344757 | N -1.246035 -0.849467 2.206085 | N -0.827142 -1.755091 1.853683 |
| | N -1.230431 -0.780125 2.311871 | N -1.813833 1.844261 1.100538 | N 1.245913 0.845098 2.208102 | N 1.539899 0.001841 2.200751 |
| | N -1.120087 2.316992 0.927890 | N 1.148505 0.994590 2.359711 | N 1.139262 -2.187666 1.015764 | N 1.194114 -2.388636 -0.147241 |
| | N -2.786955 0.073464 -0.132498 | N 0.702313 2.756875 -0.308114 | N 2.710010 -0.051956 -0.225528 | N 2.692707 0.187791 -0.381814 |
| | N 1.119164 -2.232939 1.113304 | N -2.776061 -0.554754 -0.549947 | N -1.139897 2.185128 1.020787 | N -0.809527 1.741399 1.863683 |
| | H 1.900892 2.708007 -0.318022 | H 1.420024 -2.568128 1.716027 | H -1.793271 -2.688910 0.015445 | H -1.884128 -2.493673 -0.786845 |
| | H 1.761043 2.155007 -1.812203 | H 2.300049 -2.405935 0.385324 | H -1.761352 -2.254934 -1.556082 | H -2.118287 -1.427182 -2.009801 |
| | H 0.593257 3.049949 -1.180934 | H 2.573703 -1.446180 1.644970 | H -0.505406 -3.084001 -0.921378 | H -0.815866 -2.407815 -2.015263 |

| | | | | | | | | | | | | | | | | |
|---|---|---|---|---|---|---|---|---|---|---|---|---|---|---|---|---|
| | H | 1.686688 | -1.942905 | -2.100071 | H | 0.442073 | -1.887232 | -2.855600 | H | -1.559604 | 1.964603 | -1.991358 | H | -1.919615 | 2.332349 | -1.053141 |
| | H | 0.398517 | -1.493406 | -2.941776 | H | 1.221330 | -0.500115 | -3.104173 | H | -0.307698 | 1.455337 | -2.913491 | H | -0.902210 | 2.093941 | -2.315538 |
| | H | 1.663742 | -0.518071 | -2.827331 | H | 1.945260 | -1.697277 | -2.322448 | H | -1.648807 | 0.530713 | -2.774194 | H | -2.198784 | 1.123324 | -2.122747 |
| | H | -1.899661 | -2.726055 | -0.096947 | H | -1.952553 | 1.708650 | -2.305160 | H | 1.797749 | 2.686533 | 0.022549 | H | 1.673555 | 2.702911 | 0.456689 |
| | H | -1.762463 | -2.295408 | -1.631104 | H | -0.449058 | 1.932993 | -2.824377 | H | 1.761592 | 2.258558 | -1.550482 | H | 1.457884 | 2.638632 | -1.165412 |
| | H | -0.593114 | -3.135838 | -0.931539 | H | -1.202071 | 0.536922 | -3.100434 | H | 0.508452 | 3.087152 | -0.909631 | H | 0.262812 | 3.219611 | -0.209382 |
| | H | -3.215797 | 1.006238 | -0.163084 | H | 1.107017 | 3.237269 | 0.501871 | H | 3.119365 | -0.999819 | -0.258269 | H | 3.229457 | -0.670240 | -0.188265 |
| | H | -3.203968 | -0.381492 | -0.953378 | H | 1.393170 | 2.916261 | -1.046675 | H | 3.055668 | 0.397122 | -1.086275 | H | 2.994481 | 0.471914 | -1.324773 |
| | H | -3.295412 | -0.371823 | 0.641004 | H | -0.072894 | 3.369106 | -0.578522 | H | 3.225995 | 0.440801 | 0.521684 | H | 3.123416 | 0.890136 | 0.245973 |
| | H | 1.818094 | 1.796077 | 2.111247 | H | -0.625761 | -1.734319 | 2.842492 | H | -1.834995 | -1.683488 | 2.012472 | H | -1.454740 | -2.474669 | 1.446029 |
| | H | 0.596886 | 1.263221 | 2.999494 | H | -1.344768 | -0.317055 | 3.071266 | H | -0.632218 | -1.145303 | 2.975819 | H | -0.066294 | -2.306287 | 2.275907 |
| | H | 1.862278 | 0.317851 | 2.724636 | H | -2.074108 | -1.457302 | 2.204214 | H | -1.904138 | -0.179746 | 2.627451 | H | -1.354995 | -1.379331 | 2.653437 |
| | H | -1.826178 | -1.615592 | 2.246416 | H | -2.299696 | 2.402147 | 0.391091 | H | 1.837248 | 1.677694 | 2.016033 | H | 2.112721 | 0.867677 | 2.268085 |
| | H | -0.603158 | -1.018591 | 3.090302 | H | -2.580140 | 1.435367 | 1.643941 | H | 0.631714 | 1.141949 | 2.977157 | H | 1.062197 | -0.061645 | 3.110055 |
| | H | -1.864345 | -0.092945 | 2.739827 | H | -1.426171 | 2.556087 | 1.727303 | H | 1.901630 | 0.173348 | 2.629970 | H | 2.241178 | -0.757339 | 2.225871 |
| | H | -1.630952 | 2.877574 | 0.233594 | H | 2.068424 | 1.426486 | 2.230658 | H | 1.619562 | -2.725813 | 0.259994 | H | 1.695045 | -2.553246 | -1.034744 |
| | H | -1.815261 | 2.213861 | 1.678302 | H | 0.616352 | 1.717856 | 2.853487 | H | 1.879435 | -2.045183 | 1.717326 | H | 1.904807 | -2.546800 | 0.586671 |
| | H | -0.464969 | 3.003040 | 1.323186 | H | 1.316977 | 0.291983 | 3.086257 | H | 0.495971 | -2.871279 | 1.440380 | H | 0.548990 | -3.191840 | -0.058991 |
| | H | 1.633201 | -2.845875 | 0.467332 | H | -2.897982 | -0.924966 | -1.497023 | H | -1.620929 | 2.725402 | 0.267030 | H | -1.422943 | 2.463809 | 1.436110 |
| | H | 1.811422 | -2.069404 | 1.855659 | H | -3.242013 | -1.253751 | 0.036736 | H | -1.879402 | 2.040461 | 1.722676 | H | -1.362771 | 1.371418 | 2.646867 |
| | H | 0.463568 | -2.886434 | 1.559686 | H | -3.426563 | 0.236345 | -0.524735 | H | -0.496296 | 2.867755 | 1.446603 | H | -0.044310 | 2.274478 | 2.299875 |
| | N | -1.068155 | 0.944741 | -2.341103 | N | 2.776933 | 0.553850 | -0.539438 | N | 0.999708 | -1.108944 | -2.238265 | N | 0.746356 | -0.138078 | -2.574133 |
| | H | -0.394479 | 1.243797 | -3.057319 | H | 3.420243 | -0.243457 | -0.541945 | H | 0.305762 | -1.440158 | -2.920601 | H | -0.034649 | -0.263484 | -3.235842 |
| | H | -1.679806 | 1.766696 | -2.254603 | H | 3.250592 | 1.229320 | 0.068310 | H | 1.557138 | -1.956072 | -2.001195 | H | 1.397875 | -0.909812 | -2.793411 |
| | H | -1.662941 | 0.285889 | -2.860108 | H | 2.899679 | 0.953397 | -1.474361 | H | 1.648271 | -0.518067 | -2.776328 | H | 1.232712 | 0.711695 | -2.909222 |
| | N | 2.787530 | -0.081665 | -0.121261 | N | -0.691529 | -2.755008 | -0.335296 | N | -2.710280 | 0.047497 | -0.225337 | N | -2.691004 | 0.085993 | 0.454766 |
| | H | 3.204812 | 0.302351 | -0.977474 | H | 0.081317 | -3.354686 | -0.638638 | H | -3.053805 | -0.388618 | -1.093625 | H | -3.256940 | -0.335171 | -0.295520 |
| | H | 3.217729 | -1.012972 | -0.071616 | H | -1.401179 | -2.907455 | -1.057358 | H | -3.124517 | 0.993567 | -0.242390 | H | -3.044965 | 1.056463 | 0.517145 |
| | H | 3.293973 | 0.428947 | 0.612138 | H | -1.068795 | -3.254038 | 0.476720 | H | -3.223806 | -0.460375 | 0.513542 | H | -3.038070 | -0.376008 | 1.305791 |

**Table S3.** Energies (hartrees) for the $Th(12C4X)_2{}^q$ and $Th(15C5X)_2{}^q$ species ($q$ = 4+, 3+, 2+, 1+, 0 and X = N, O). Different spin states are reported (spin is given in parenthesis).

| $q$ | $Th(12C4N)_2$ | $Th(15C5N)_2$ | $Th(12C4O)_2$ | $Th(15C5O)_2$ |
|---|---|---|---|---|
| 4 (S=0) | -1476.839126 | -1744.736150 | -1635.730076 | -1943.393506 |
| 3 (S=$^1/_2$) | -1477.326154 | -1745.193644 | -1636.233438 | -1943.850463 |
| 2 (S=0) | -1477.664916 | -1745.485247 | -1636.564960 | -1944.145241 |
| 2 (S=1) | -1477.667265 | -1745.476753 | -1636.566613 | -1944.140814 |
| 1 (S=$^1/_2$) | -1477.849360 | -1745.657442 | -1636.749266 | -1944.296001 |
| 1 (S=$^3/_2$) | -1477.851669 | -1745.651736 | -1636.737169 | -1944.287642 |
| 0 (S=0) | -1477.943915 | -1745.740132 | -1636.813739 | -1944.371739 |
| 0 (S=1) | -1477.944112 | -1745.750461 | -1636.813744 | -1944.366650 |
| 0 (S=2) | -1477.942209 | -1745.745896 | -1636.804662 | -1944.362478 |

**Table S4.** Cartesian coordinates (Å) for the $Th(12C4X)_2^q$ and $Th(15C5X)_2^q$ species ($q$ = 4+, 3+, 2+, 1+, 0 and X = N, O). Different spin states are reported (spin is given in parenthesis).

| $q$ | $Th(12C4O)_2$ | $Th(15C5O)_2$ | $Th(12C4N)_2$ | $Th(15C5N)_2$ |
|---|---|---|---|---|
| 4 | O -1.940506 0.672850 1.411955 | O 0.410822 1.304549 -2.113994 | N 1.644022 0.484427 -1.980167 | N -0.275437 1.368031 -2.427902 |
| | O -1.275173 -1.850496 0.898317 | O -0.629678 -1.093440 -2.218302 | N 1.422423 -2.160739 -0.600453 | N -1.433812 -1.214048 -1.907143 |
| (S=0) | O -1.275366 1.850461 -0.898025 | O -1.511226 2.048634 -0.509600 | N 1.852453 1.841190 0.564622 | N -1.614511 2.297354 0.010570 |
| | O -1.940587 -0.672862 -1.411474 | O -1.309323 -2.198805 0.094962 | N 1.560788 -0.676384 1.958933 | N -1.273570 -2.453219 0.718064 |
| | C -2.744791 1.851418 1.059500 | O -2.638051 -0.056674 0.602913 | C 2.138285 1.901109 -1.930272 | N -2.395008 0.008350 1.542016 |
| | C -1.901315 -1.601124 2.204288 | C -1.596873 -2.187628 -2.294350 | C 2.224210 -1.932799 -1.856478 | C -2.561024 -2.041584 -1.398397 |
| | C -1.941227 2.703123 0.099431 | C -1.496577 2.726784 -1.797926 | C 2.750466 2.232347 -0.582446 | C -2.188338 2.557311 -1.338216 |
| | C -1.941116 -2.703198 -0.099042 | C -1.393454 -3.051555 -1.079707 | C 2.232847 -2.626946 0.582435 | C -2.057827 -3.063483 -0.402174 |
| | C -1.901649 1.601084 -2.203922 | C 0.689070 0.537628 -3.320155 | C 2.654239 1.564942 1.812686 | C -0.711138 0.462372 -3.532446 |
| | C -2.744772 -1.851502 -1.059036 | C -0.432663 -0.445234 -3.511409 | C 2.718146 -1.486078 1.456959 | C -1.854854 -0.419645 -3.095380 |
| | C -2.724111 0.332095 -2.142378 | C -2.732447 2.283270 0.253948 | C 1.978900 0.550144 2.716436 | C -2.686707 2.386247 1.043798 |
| | C -2.723909 -0.332211 2.142843 | C -2.355325 -2.342584 1.088538 | C 2.760517 -0.518662 -1.975330 | C -2.080569 -2.373164 1.965947 |
| | H -2.981322 2.418850 1.971254 | C -3.541171 1.022997 0.250191 | H 1.287538 2.560144 -2.156682 | C -3.385838 1.060290 1.198077 |
| | H -3.682687 1.488978 0.615806 | C -3.382808 -1.283473 0.816905 | H 2.882551 2.080560 -2.721009 | C -3.095558 -1.267375 1.851694 |
| | H -1.066593 -1.528160 2.916054 | C -0.077898 2.671232 -2.283926 | H 1.569546 -2.161057 -2.711267 | C -1.088821 2.622920 -2.369195 |
| | H -2.513681 -2.465788 2.495020 | H 0.001203 2.967551 -3.337167 | H 3.056525 -2.649816 -1.908908 | H -1.511325 2.854897 -3.357364 |
| | H -2.578225 3.438659 -0.410426 | H -1.413815 -2.768172 -3.209255 | H 3.701731 1.704032 -0.453345 | H -3.060253 -2.562426 -2.229387 |
| | H -1.130387 3.243003 0.604771 | H -2.603830 -1.749202 -2.348290 | H 2.994717 3.303019 -0.541416 | H -3.303625 -1.362903 -0.961001 |
| | H -2.578048 -3.438743 0.410884 | H -1.809273 3.771902 -1.666209 | H 3.089527 -3.227422 0.242976 | H -2.745759 3.506054 -1.341346 |
| | H -1.130309 -3.243060 -0.604455 | H -2.201651 2.223844 -2.475822 | H 1.596763 -3.305964 1.170285 | H -2.914504 1.768012 -1.566555 |
| | H -1.067001 1.528196 -2.915783 | H -2.223122 -3.761638 -0.970244 | H 2.838954 2.491572 2.373560 | H -2.902900 -3.648019 -0.010406 |
| | H -2.514121 2.465702 -2.494569 | H -0.455387 -3.614774 -1.138178 | H 3.640018 1.202986 1.500385 | H -1.420869 -3.795489 -0.922957 |
| | H -2.981313 -2.418917 -1.970798 | H 1.651404 0.037841 -3.153336 | H 3.299034 -1.886704 2.301150 | H 0.147766 -0.163301 -3.823784 |
| | H -3.682671 -1.489144 -0.615280 | H 0.793495 1.208073 -4.182071 | H 3.392699 -0.824012 0.902679 | H -0.986749 1.039180 -4.426829 |
| | H -3.671082 0.471431 -1.602247 | H -1.376218 0.034665 -3.810413 | H 2.660912 0.286089 3.539037 | H -2.744102 0.163437 -2.827713 |
| | H -2.943749 -0.038868 -3.153939 | H -0.166169 -1.203938 -4.261051 | H 1.078140 0.971074 3.188462 | H -2.155455 -1.086414 -3.916829 |
| | H -2.943531 0.038694 3.154429 | H -3.293516 3.117432 -0.184957 | H 3.356875 -0.426658 -2.895165 | H -3.411214 3.172514 0.786081 |
| | H -3.670892 -0.471610 1.602749 | H -2.431941 2.560175 1.268776 | H 3.435400 -0.283618 -1.144524 | H -2.236956 2.698987 1.997905 |
| | Th -0.000022 -0.000003 0.000037 | H -1.871467 -2.204356 2.063815 | Th -0.000322 -0.001221 -0.013429 | H -1.399273 -2.178719 2.810217 |
| | H 2.980871 2.419003 -1.971409 | H -2.779997 -3.353854 1.057509 | H 1.193514 0.385670 -2.897306 | H -2.573287 -3.332680 2.181357 |
| | H 2.943088 0.038875 -3.154595 | H -4.352675 1.085991 0.989756 | H 1.303960 2.683078 0.775886 | H -4.166860 1.135650 1.969749 |
| | C 2.744518 1.851547 -1.059624 | H -3.980705 0.803153 -0.734847 | H 1.087334 -1.274927 2.646001 | H -3.893493 0.763889 0.269609 |
| | H 1.130127 3.243065 -0.604635 | H -3.976639 -1.498594 -0.084832 | H 0.826045 -2.961588 -0.826985 | H -3.821219 -1.472601 1.055969 |
| | C 2.723643 -0.332062 -2.142982 | H -4.069880 -1.159046 1.666690 | H -1.281441 -2.954797 -1.572983 | H -3.676615 -1.178630 2.781272 |
| | O 1.940263 0.672921 -1.411959 | H 0.570178 3.319766 -1.683993 | H -1.300555 -2.455564 1.326708 | H -0.410860 3.457197 -2.130805 |
| | H 1.066346 -1.528163 -2.915997 | Th -0.000094 0.008249 -0.000056 | H -1.194491 -0.983382 -2.751396 | Th 0.000076 0.013505 0.000053 |
| | C 1.941055 2.703199 -0.099422 | O -0.412394 1.305644 2.113055 | C -2.134394 -2.266018 -1.487743 | N 0.275017 1.361992 2.431042 |
| | H 3.682508 1.489169 -0.616078 | O 0.631791 -1.090581 2.218823 | N -1.851249 -1.680202 0.939574 | N 1.434313 -1.218340 1.904236 |
| | C 1.901160 -1.601053 -2.204331 | O 1.507502 2.051946 0.507395 | N -1.644765 -0.889746 -1.833590 | N 1.613545 2.297641 -0.004886 |
| | H 3.670717 -0.471387 -1.603029 | O 1.312381 -2.197225 -0.093937 | H -1.084333 -0.279656 3.324647 | N 1.274484 -2.450883 -0.723932 |
| | H 2.578092 3.438739 0.410381 | O 2.638103 -0.053457 -0.601393 | H -2.982879 -3.349422 0.165906 | N 2.394891 0.012948 -1.542018 |
| | H 2.513564 -2.465668 -2.495127 | C 1.600703 -2.183282 2.295376 | C -2.745259 -2.309883 -0.099660 | C 2.561895 -2.044087 1.393406 |
| | O 1.275354 1.850466 0.898078 | C 1.492353 2.730896 1.795258 | H -2.878634 -2.609250 -2.222391 | C 2.187566 2.554490 1.344408 |
| | O 1.275186 -1.850452 -0.898288 | C 1.397696 -3.048538 1.081640 | C -1.982469 0.034301 2.771199 | C 2.058978 -3.063721 0.394748 |

| | | | | |
|---|---|---|---|---|
| | C 1.901808 1.601086 2.203904 | C -0.689477 0.539200 3.319770 | C -2.657096 -1.146790 2.098378 | C 0.710678 0.453756 3.533494 |
| | H 2.514212 2.465772 2.494490 | C 0.433602 -0.442059 3.511657 | N -1.560288 1.073408 1.773772 | C 1.854847 -0.426745 3.094523 |
| | C 1.941308 -2.703115 0.099001 | C 2.728626 2.287095 -0.256066 | H -1.086115 1.801956 2.320345 | C 2.685315 2.389765 -1.038231 |
| | H 2.578190 -3.438624 -0.411039 | C 2.359299 -2.339788 -1.086671 | H -2.846070 -1.934749 2.840523 | C 2.081451 -2.367580 -1.971622 |
| | O 1.940890 -0.672928 1.411669 | C 3.539373 1.028129 -0.249644 | H -0.823900 2.715450 -1.436901 | C 3.385247 1.064623 -1.195881 |
| | H 1.067260 1.528097 2.915873 | C 3.385016 -1.279058 -0.814791 | N -1.422554 1.984161 -1.043178 | C 3.095992 -1.261690 -1.854749 |
| | H 3.671276 0.471630 1.601908 | C 0.074049 2.673260 2.282034 | H -1.581027 1.543380 -3.106697 | C 1.088142 2.617217 2.375657 |
| | C 2.724455 0.332207 2.142280 | H -0.004948 2.969977 3.335169 | C -2.763833 0.089311 -2.037214 | H 1.510668 2.846622 3.364414 |
| | H 3.682872 -1.488926 0.614949 | H 1.419099 -2.763190 3.210971 | C -2.230534 1.497882 -2.219378 | H 3.061446 -2.566747 2.223059 |
| | H 1.130609 -3.243030 0.604531 | H 2.607016 -1.743280 2.348243 | H -3.699900 -1.772237 -0.084394 | H 3.304119 -1.363984 0.957590 |
| | C 2.745123 -1.851436 1.058896 | H 1.803199 3.776467 1.662729 | H -2.666492 0.466459 3.517437 | H 2.744688 3.503398 1.349763 |
| | H 2.944387 -0.038651 3.153816 | H 2.198585 2.229598 2.473174 | H -3.361368 -0.195162 -2.916013 | H 2.913999 1.764849 1.570733 |
| | H 2.981946 -2.418909 1.970548 | H 2.227774 -3.758228 0.972656 | H -1.584735 3.475927 0.448732 | H 2.904190 -3.647083 0.001522 |
| | | H 0.460058 -3.612375 1.141043 | C -2.226393 2.692796 0.017094 | H 1.422248 -3.797160 0.913821 |
| | | H -1.651198 0.038068 3.153497 | H -3.640839 -0.857007 1.712841 | H -0.148083 -0.172945 3.823028 |
| | | H -0.794605 1.210109 4.181237 | C -2.715364 1.762765 1.111606 | H 0.985823 1.028460 4.429369 |
| | | H 1.376455 0.039227 3.810622 | H -3.436977 0.032545 -1.174294 | H 2.743927 0.157347 2.828489 |
| | | H 0.167983 -1.200866 4.261498 | H -3.391674 1.000771 0.708348 | H 2.155512 -1.095441 3.914378 |
| | | H 3.288121 3.123073 0.181406 | H -3.064843 2.187128 -2.415439 | H 3.409340 3.175955 -0.778938 |
| | | H 2.428191 2.561433 -1.271618 | H -3.295203 2.333417 1.852364 | H 2.234925 2.704337 -1.991419 |
| | | H 1.875899 -2.202639 -2.062309 | H -3.080771 3.214251 -0.438803 | H 1.400046 -2.171425 -2.815410 |
| | | H 2.785519 -3.350395 -1.054945 | | H 2.574564 -3.326389 -2.189289 |
| | | H 4.351289 1.091156 -0.988745 | | H 4.165966 1.142257 -1.967631 |
| | | H 3.978538 0.810479 0.736040 | | H 3.893360 0.766304 -0.268282 |
| | | H 3.978851 -1.493000 0.087216 | | H 3.821780 -1.468506 -1.059550 |
| | | H 4.072194 -1.153712 -1.664353 | | H 3.676955 -1.170503 -2.784153 |
| | | H -0.575509 3.320411 1.682219 | | H 0.410014 3.452000 2.139539 |
| | | | | H 0.749212 -1.887842 2.271091 |
| | | | | H -0.651421 1.684876 2.707719 |
| | | | | H 1.002545 3.097311 -0.184806 |
| | | | | H 0.543252 -3.128382 -0.936695 |
| | | | | H 0.650807 1.691849 -2.704153 |
| | | | | H -1.004204 3.097067 0.192744 |
| | | | | H -0.748419 -1.882293 -2.275756 |
| | | | | H -0.542155 -3.131034 0.929066 |
| | | | | H -2.004070 0.294126 2.443342 |
| | | | | H 2.003659 0.300557 -2.442627 |
| 3 (S=$^{1}/_{2}$) | O 2.081076 -0.821358 1.291993 | O -0.034789 -1.320132 -2.209898 | N -1.733434 0.675174 1.934545 | N 0.303037 -1.402595 -2.437083 |
| | O 1.359358 1.772330 1.096698 | O 0.766351 1.248340 -2.276122 | N -1.507295 -2.095166 0.825000 | N 1.316881 1.330248 -2.049942 |
| | O 1.355626 -1.772012 -1.097246 | O 1.546775 -2.191184 -0.238599 | N -1.892039 1.825027 -0.763803 | N 1.619710 -2.362990 0.024030 |
| | O 2.082796 0.819869 -1.292224 | O 1.371232 2.212433 0.187604 | N -1.597869 -0.870873 -1.901017 | N 1.324137 2.466556 0.668181 |
| | C 2.888666 -1.917593 0.798177 | O 2.625861 -0.015767 0.953786 | C -2.186015 2.079368 1.723634 | N 2.425428 -0.047270 1.597728 |
| | C 2.006026 1.351988 2.329832 | C 1.803323 2.242612 -2.191464 | C -2.338585 -1.729036 2.014829 | C 2.460966 2.115507 -1.538345 |
| | C 2.056574 -2.683973 -0.207666 | C 1.832775 -2.685661 -1.561388 | C -2.781747 2.296206 0.343175 | C 2.229446 -2.538634 -1.313649 |
| | C 2.061706 2.683014 0.206861 | C 1.544098 3.072016 -0.960467 | C -2.274827 -2.663442 -0.327720 | C 2.024983 3.106262 -0.480033 |
| | C 2.002957 -1.352884 -2.330468 | C -0.256407 -0.551134 -3.416033 | C -2.686208 1.383303 -1.952711 | C 0.655701 -0.491385 -3.555100 |
| | C 2.892318 1.914902 -0.798891 | C 0.799739 0.518034 -3.513700 | C -2.745951 -1.619824 -1.324794 | C 1.757939 0.464968 -3.167511 |
| | C 2.839372 -0.107575 -2.109403 | C 2.634844 -2.365117 0.695026 | C -2.008792 0.282347 -2.751413 | C 2.654721 -2.445468 1.082374 |
| | C 2.839712 0.104866 2.108792 | C 2.340331 2.309874 1.247552 | C -2.853824 -0.301408 1.985466 | C 2.179724 2.372772 1.874194 |
| | H 3.176700 -2.574826 1.631717 | C 3.496725 -1.132582 0.715984 | H -1.315897 2.733616 1.880802 | C 3.372987 -1.128031 1.255013 |
| | H 3.804349 -1.503485 0.351304 | C 3.357976 1.213488 1.085987 | H -2.934037 2.369599 2.477903 | C 3.155316 1.228620 1.759241 |
| | H 1.184838 1.172690 3.034250 | C 0.544574 -2.641765 -2.336973 | H -1.713126 -1.875256 2.908440 | C 1.160695 -2.618007 -2.379549 |
| | H 2.625901 2.169061 2.724460 | H 0.720129 -2.876979 -3.394424 | H -3.188175 -2.421142 2.116125 | H 1.626544 -2.813800 -3.356687 |

| | | | | |
|---|---|---|---|---|
| | H 2.676629 -3.377178 -0.791713 | H 1.777289 2.884288 -3.085185 | H -3.731815 1.756675 0.260646 | H 2.964741 2.659074 -2.353924 |
| | H 1.269513 -3.266193 0.289344 | H 2.781471 1.735771 -2.150834 | H -3.032757 3.360066 0.226101 | H 3.188770 1.400338 -1.133515 |
| | H 2.683026 3.375276 0.790682 | H 2.195587 -3.721950 -1.498188 | H -3.140569 -3.238480 0.034307 | H 2.841655 -3.454188 -1.348246 |
| | H 1.275426 3.266459 -0.289970 | H 2.611271 -2.063574 -2.030638 | H -1.616783 -3.384493 -0.835848 | H 2.904576 -1.694620 -1.498160 |
| | H 1.181975 -1.171589 -3.034615 | H 2.375522 3.768428 -0.790611 | H -2.902152 2.227453 -2.622857 | H 2.898774 3.678593 -0.134084 |
| | H 2.620890 -2.171280 -2.725376 | H 0.618310 3.650525 -1.070861 | H -3.660172 1.033903 -1.592853 | H 1.353921 3.852176 -0.934878 |
| | H 3.181378 2.571353 -1.632693 | H -1.259180 -0.114773 -3.322922 | H -3.329462 -2.114307 -2.117000 | H -0.244080 0.081537 -3.829861 |
| | H 3.807354 1.499376 -0.351981 | H -0.241072 -1.209030 -4.293907 | H -3.416545 -0.903415 -0.839563 | H 0.944605 -1.060547 -4.451333 |
| | H 3.780018 -0.332773 -1.585874 | H 1.803717 0.084276 -3.653604 | H -2.693228 -0.041724 -3.550945 | H 2.652395 -0.082292 -2.843491 |
| | H 3.084229 0.358213 -3.075284 | H 0.590708 1.195529 -4.355745 | H -1.104859 0.662332 -3.250811 | H 2.059287 1.066926 -4.038518 |
| | H 3.083853 -0.361215 3.074711 | H 3.220454 -3.255104 0.429035 | H -3.486149 -0.127461 2.869927 | H 3.386521 -3.237069 0.857809 |
| | H 3.780665 0.328002 1.584946 | H 2.176365 -2.529452 1.675627 | H -3.488691 -0.142823 1.107599 | H 2.173691 -2.745368 2.025414 |
| | Th 0.000000 0.001571 0.000010 | H 1.778157 2.195075 2.183039 | Th 0.000002 -0.000422 0.035186 | H 1.527381 2.216133 2.747813 |
| | H -3.176688 -2.574818 -1.631737 | H 2.816237 3.299220 1.247197 | H -1.304040 0.662615 2.864105 | H 2.719898 3.314951 2.053567 |
| | H -3.083817 -0.361204 -3.074730 | H 4.243929 -1.207457 1.520869 | H -1.362254 2.645027 -1.067063 | H 4.156773 -1.234464 2.021788 |
| | C -2.888661 -1.917585 -0.798194 | H 4.029914 -0.979214 -0.236667 | H -1.123268 -1.522676 -2.532321 | H 3.884177 -0.842909 0.325432 |
| | H -1.269519 -3.266189 -0.289338 | H 3.978310 1.362767 0.186904 | H -0.926364 -2.869659 1.149945 | H 3.810359 1.364127 0.889462 |
| | C -2.839685 0.104875 -2.108808 | H 4.022788 1.163961 1.961999 | H 1.317239 -2.733110 1.881432 | H 3.814920 1.203982 2.640389 |
| | O -2.081061 -0.821354 -1.292003 | H -0.179560 -3.358870 -1.929182 | H 1.363874 -2.645409 -1.066203 | H 0.512201 -3.484895 -2.177189 |
| | H -1.184795 1.172693 -3.034245 | Th 0.000059 -0.005371 -0.000094 | H 1.304221 -0.661957 2.864494 | Th 0.000365 -0.013099 0.000560 |
| | C -2.056585 -2.683967 0.207661 | O 0.025045 -1.311819 2.214581 | C 2.187032 -2.078438 1.724318 | N -0.304435 -1.393456 2.441867 |
| | H -3.804348 -1.503472 -0.351332 | O -0.757759 1.262469 2.271318 | N 1.893234 -1.825029 -0.763230 | N -1.316095 1.338797 2.044477 |
| | C -2.005991 1.351993 -2.329838 | O -1.561210 -2.181362 0.246285 | N 1.733661 -0.674442 1.934956 | N -1.621383 -2.362268 -0.015232 |
| | H -3.780641 0.328015 -1.584971 | O -1.356886 2.220658 -0.195474 | H 1.105765 -0.663612 -3.250909 | N -1.323176 2.465221 -0.677909 |
| | H -2.676650 -3.377169 0.791700 | O -2.625887 -0.002377 -0.952801 | H 3.034850 -3.358985 0.227288 | N -2.425221 -0.051983 -1.597581 |
| | H -2.625858 2.169069 -2.724474 | C -1.787776 2.263635 2.183407 | C 2.783094 -2.295259 0.343999 | C -2.460017 2.122497 1.530149 |
| | O -1.355646 -1.772006 1.097249 | C -1.851071 -2.667966 1.571136 | H 2.935088 -2.368149 2.478755 | C -2.232404 -2.530886 1.322712 |
| | O -1.359338 1.772334 -1.096696 | C -1.523995 3.086303 0.948820 | C 2.009268 -0.282890 -2.751287 | C -2.023903 3.109140 0.468007 |
| | C -2.002992 -1.352877 2.330463 | C 0.253475 -0.539545 3.417409 | C 2.687217 -1.383253 -1.952202 | C -0.655095 -0.477243 3.556404 |
| | H -2.620933 -2.171271 2.725363 | C -0.795095 0.537353 3.511841 | N 1.597467 0.870340 -1.901298 | C -1.757011 0.478239 3.165795 |
| | C -2.061695 2.683021 -0.206868 | C -2.650800 -2.350824 -0.686388 | H 1.122710 1.521663 -2.532982 | C -2.655444 -2.448550 -1.074138 |
| | H -2.683004 3.375285 -0.790697 | C -2.324875 2.320246 -1.256270 | H 2.903642 -2.227482 -2.622093 | C -2.179023 2.366831 -1.883430 |
| | O -2.082811 0.819873 1.292214 | C -3.504242 -1.112496 -0.711227 | H 0.924281 2.869144 1.148964 | C -3.373254 -1.131594 -1.252516 |
| | H -1.182018 -1.171584 3.034621 | C -3.349781 1.231188 -1.090529 | N 1.506160 2.095222 0.824380 | C -3.154850 1.223373 -1.763978 |
| | H -3.780041 -0.332761 1.585849 | C -0.563286 -2.628897 2.347698 | H 1.711887 1.876360 2.907942 | C -1.164432 -2.607501 2.389594 |
| | C -2.839400 -0.107565 2.109387 | H -0.741581 -2.857485 3.406152 | C 2.853518 0.302750 1.985714 | H -1.631215 -2.797711 3.367380 |
| | H -3.807355 1.499390 0.351952 | H -1.756542 2.908690 3.074523 | C 2.337522 1.730120 2.014457 | H -2.963377 2.669347 2.343785 |
| | H -1.275419 3.266463 0.289974 | H -2.769478 1.763484 2.145625 | H 3.732806 -1.755104 0.261427 | H -3.188128 1.405978 1.128295 |
| | C -2.892323 1.914912 0.798873 | H -2.220532 -3.702172 1.512388 | H 2.693705 0.041419 -3.550729 | H -2.846376 -3.445119 1.360974 |
| | H -3.084265 0.358226 3.075265 | H -2.625911 -2.038868 2.037097 | H 3.485822 0.129446 2.870316 | H -2.905983 -1.684713 1.503002 |
| | H -3.181389 2.571363 1.632673 | H -2.351469 3.786789 0.776408 | H 1.614873 3.384245 -0.836771 | H -2.897529 3.680551 0.120102 |
| | | H -0.594787 3.659880 1.056302 | C 2.273341 2.663733 -0.328424 | H -1.352475 3.856472 0.919961 |
| | | H 1.259099 -0.110479 3.321249 | H 3.660978 -1.033235 -1.592393 | H 0.245384 0.096174 3.827781 |
| | | H 0.234766 -1.193820 4.297915 | C 2.745098 1.620074 -1.325152 | H -0.943524 -1.042359 4.455346 |
| | | H -1.801939 0.111165 3.654410 | H 3.488583 0.144186 1.107987 | H -2.652024 -0.069793 2.844600 |
| | | H -0.580608 1.216731 4.350990 | H 3.415947 0.904138 -0.839577 | H -2.057395 1.083861 4.034585 |
| | | H -3.242470 -3.235875 -0.417307 | H 3.186705 2.422754 2.115549 | H -3.387706 -3.239061 -0.847210 |
| | | H -2.193979 -2.521482 -1.666682 | H 3.328484 2.114576 -2.117437 | H -2.173545 -2.752279 -2.015490 |
| | | H -1.763152 2.197770 -2.191039 | H 3.138719 3.239401 0.033469 | H -1.526804 2.206687 -2.756517 |
| | | H -2.794104 3.312764 -1.260170 | | H -2.719084 3.308390 -2.066386 |
| | | H -4.252102 -1.184990 -1.515729 | | H -4.155931 -1.240629 -2.020053 |
| | | H -4.036277 -0.952371 0.240946 | | H -3.885825 -0.843210 -0.324708 |
| | | H -3.969537 1.388350 -0.192393 | | H -3.809863 1.362310 -0.894725 |

| | | | | |
|---|---|---|---|---|
| | | H -4.014540 1.182231 -1.966612 | | H -3.814463 1.195519 -2.645036 |
| | | H 0.156418 -3.353050 1.944514 | | H -0.517572 -3.476634 2.191711 |
| | | | | H -0.665174 2.013590 2.457029 |
| | | | | H 0.626205 -1.741295 2.661576 |
| | | | | H -1.026499 -3.182712 -0.147202 |
| | | | | H -0.574948 3.104060 -0.938639 |
| | | | | H -0.628036 -1.749743 -2.656054 |
| | | | | H 1.024066 -3.182170 0.160357 |
| | | | | H 0.666537 2.003744 -2.465534 |
| | | | | H 0.575454 3.105813 0.926434 |
| | | | | H 2.069782 -0.266839 2.531476 |
| | | | | H -2.068397 -0.274316 -2.530210 |
| 2 | O -2.236502 1.088546 1.092190 | O -0.416021 -1.677803 -1.984082 | N 1.972667 -1.355481 -1.431762 | N 0.270074 1.406979 2.442874 |
| | O -1.516254 -1.471937 1.467033 | O -1.049997 1.045751 -2.337098 | N 1.796262 -1.608676 1.489947 | N 1.166873 -1.426225 2.140666 |
| (S=0) | O -1.516256 1.471937 -1.467032 | O 2.092315 -1.562359 -0.830466 | N 1.979275 1.504465 -1.283615 | N 1.628660 2.396390 0.005738 |
| | O -2.236501 -1.088547 -1.092190 | O 0.267585 2.596406 -0.395158 | N 1.710852 1.330580 1.567023 | N 1.307058 -2.493880 -0.622527 |
| | C -3.019964 2.044474 0.363914 | O 2.603382 1.129160 -0.112481 | C 2.351104 -0.566769 -2.622150 | N 2.461513 0.044849 -1.577111 |
| | C -2.190286 -0.805228 2.548438 | C -0.465392 2.302576 -2.695103 | C 2.666278 -2.470662 0.652169 | C 2.298559 -2.245573 1.667945 |
| | C -2.190120 2.551162 -0.796643 | C 1.983678 -1.952618 -2.204469 | C 2.878215 0.804743 -2.234738 | C 2.210210 2.561777 1.352041 |
| | C -2.190117 -2.551163 0.796644 | C -0.430745 3.210685 -1.491979 | C 2.509891 -0.800996 2.508309 | C 1.899133 -3.186008 0.549071 |
| | C -2.190288 0.805227 -2.548437 | C -1.379346 -1.233646 -2.954144 | C 2.714229 2.447879 -0.406509 | C 0.573255 0.488886 3.563298 |
| | C -3.019962 -2.044475 -0.363913 | C -1.033861 0.146453 -3.450658 | C 2.902973 0.573902 2.000053 | C 1.637395 -0.522999 3.208185 |
| | C -3.020067 -0.357030 -2.045555 | C 3.399553 -1.100049 -0.465094 | C 2.035705 2.629803 0.941212 | C 2.676026 2.449319 -1.033171 |
| | C -3.020066 0.357029 2.045555 | C 1.475125 3.210676 0.075305 | C 3.128035 -1.784658 -0.620392 | C 2.228162 -2.389454 -1.771561 |
| | H -3.303696 2.881470 1.020891 | C 3.593104 0.357582 -0.797610 | H 1.450912 -0.464604 -3.244333 | C 3.386714 1.123623 -1.190268 |
| | H -3.943524 1.553639 0.018615 | C 2.675618 2.503436 -0.494982 | H 3.115493 -1.082614 -3.227825 | C 3.189083 -1.235271 -1.619229 |
| | H -1.388795 -0.453297 3.209581 | C 0.627114 -2.577117 -2.408005 | H 2.082481 -3.363482 0.385872 | C 1.123117 2.619929 2.403603 |
| | H -2.816946 -1.517292 3.104559 | H 0.492633 -2.840630 -3.466010 | H 3.543942 -2.817925 1.220850 | H 1.577400 2.813148 3.387982 |
| | H -2.816688 3.109807 -1.506809 | H -1.064117 2.776818 -3.490163 | H 3.857170 0.700201 -1.751607 | H 2.734333 -2.838423 2.491851 |
| | H -1.388521 3.210971 -0.442454 | H 0.554812 2.111957 -3.069091 | H 3.055053 1.396609 -3.145446 | H 3.066130 -1.544097 1.318725 |
| | H -2.816685 -3.109808 1.506809 | H 2.762566 -2.695245 -2.440439 | H 3.406712 -1.325971 2.874598 | H 2.812536 3.485617 1.412022 |
| | H -1.388517 -3.210971 0.442454 | H 2.106634 -1.066869 -2.847512 | H 1.835345 -0.685657 3.369519 | H 2.878463 1.713889 1.537416 |
| | H -1.388796 0.453297 -3.209581 | H 0.057392 4.155678 -1.767372 | H 2.849180 3.432253 -0.879679 | H 2.776577 -3.781412 0.250494 |
| | H -2.816948 1.517291 -3.104558 | H -1.447881 3.431868 -1.143759 | H 3.724169 2.047670 -0.257640 | H 1.169874 -3.918360 0.931886 |
| | H -3.303694 -2.881472 -1.020890 | H -2.348416 -1.233122 -2.438373 | H 3.457041 1.112669 2.786234 | H -0.354170 -0.043305 3.828690 |
| | H -3.943523 -1.553642 -0.018614 | H -1.426620 -1.942751 -3.791541 | H 3.582313 0.479154 1.145800 | H 0.876206 1.050508 4.461380 |
| | H -3.943560 -0.013372 -1.553443 | H -0.026357 0.155821 -3.900038 | H 2.687835 3.241985 1.586621 | H 2.539863 -0.015594 2.844802 |
| | H -3.303921 -1.011175 -2.884725 | H -1.765685 0.476593 -4.205359 | H 1.091377 3.179782 0.819798 | H 1.926198 -1.089517 4.109513 |
| | H -3.303921 1.011174 2.884726 | H 4.164282 -1.705728 -0.972946 | H 3.786533 -2.464899 -1.185276 | H 3.420017 3.231986 -0.809014 |
| | H -3.943560 0.013370 1.553444 | H 3.487476 -1.260101 0.613856 | H 3.727195 -0.899901 -0.376468 | H 2.211129 2.745645 -1.985741 |
| | Th -0.000000 0.000001 0.000000 | H 1.452100 3.122643 1.169204 | Th -0.000071 -0.131352 -0.005895 | H 1.624145 -2.241910 -2.680855 |
| | H 3.303860 2.884152 -1.012931 | H 1.491142 4.276322 -0.190821 | H 1.512781 -2.205269 -1.762261 | H 2.790426 -3.325933 -1.918019 |
| | H 3.303757 1.019136 -2.882052 | H 4.599652 0.677218 -0.481553 | H 1.327744 2.056945 -1.840722 | H 4.204313 1.236543 -1.923337 |
| | C 3.020027 2.045372 -0.358275 | H 3.491738 0.536943 -1.880937 | H 1.176277 1.531409 2.413870 | H 3.847845 0.831637 -0.237910 |
| | H 1.388658 3.209776 0.451276 | H 2.681237 2.568746 -1.596509 | H 1.189448 -2.243480 2.005741 | H 3.757463 -1.333855 -0.686017 |
| | C 3.020002 0.362670 -2.044662 | H 3.605306 2.952677 -0.109587 | H -1.458282 -0.823752 3.173809 | H 3.925683 -1.253655 -2.439872 |
| | O 2.236502 1.091531 -1.089211 | H 0.532424 -3.488561 -1.801726 | H -1.333148 1.837345 2.056615 | H 0.472944 3.485125 2.196006 |
| | H 1.388657 -0.444476 -3.210782 | Th 0.006353 -0.009379 -0.000399 | H -1.516245 -2.389690 1.507983 | Th 0.001360 0.005153 -0.000491 |
| | C 2.190202 2.548931 0.803655 | O 1.534579 -0.857432 1.960794 | C -2.356989 -0.856587 2.542052 | N -0.222435 1.373841 -2.465420 |
| | H 3.943533 1.553506 -0.014297 | O -0.046399 1.439318 2.366390 | N -1.983922 1.350122 1.440923 | N -1.216524 -1.423928 -2.121988 |
| | C 2.190204 -0.798217 -2.550673 | O -0.261003 -2.604298 0.794627 | N -1.974946 -1.508061 1.272587 | N -1.546744 2.447837 -0.041223 |
| | H 3.943550 0.017690 -1.553580 | O -2.090588 1.554839 0.428713 | H -1.094322 3.253151 -0.460036 | N -1.393871 -2.442778 0.659341 |
| | H 2.816804 3.105572 1.515363 | O -2.591616 -1.156655 0.110754 | H -3.062852 1.036825 3.277562 | N -2.457425 0.149074 1.578965 |

| | | | | | | | | | | | | | | | | |
|---|---|---|---|---|---|---|---|---|---|---|---|---|---|---|---|---|
| | H | 2.816831 | -1.508741 | -3.108797 | C | -1.370731 | 1.838355 | 2.734573 | C | -2.884083 | 0.549151 | 2.307424 | C | -2.377146 | -2.192592 | -1.634568 |
| | O | 1.516257 | 1.467908 | 1.471061 | C | 0.111956 | -2.813216 | 2.161640 | H | -3.122595 | -1.436638 | 3.084848 | C | -2.123676 | 2.606228 | -1.390265 |
| | O | 1.516255 | -1.467908 | -1.471061 | C | -2.064766 | 2.457625 | 1.548078 | C | -2.037365 | 2.718378 | -0.643447 | C | -2.011144 | -3.130058 | -0.501820 |
| | C | 2.190206 | 0.798217 | 2.550672 | C | 1.862171 | 0.135447 | 2.947221 | C | -2.718010 | 2.385654 | 0.674022 | C | -0.556203 | 0.449586 | -3.571808 |
| | H | 2.816834 | 1.508740 | 3.108796 | C | 0.613364 | 0.801169 | 3.464466 | N | -1.708777 | 1.497872 | -1.409629 | C | -1.654848 | -0.519466 | -3.202118 |
| | C | 2.190200 | -2.548932 | -0.803655 | C | -1.485011 | -3.253343 | 0.425326 | H | -1.172226 | 1.793118 | -2.226982 | C | -2.591397 | 2.551300 | 0.996495 |
| | H | 2.816801 | -3.105573 | -1.515364 | C | -3.362673 | 1.085876 | -0.039307 | H | -2.854574 | 3.310443 | 1.254747 | C | -2.308292 | -2.289540 | 1.808273 |
| | O | 2.236501 | -1.091531 | 1.089210 | C | -2.691100 | -2.418920 | 0.774298 | H | -1.182343 | -2.006620 | -2.239923 | C | -3.345457 | 1.252384 | 1.174521 |
| | H | 1.388659 | 0.444477 | 3.210782 | C | -3.650355 | -0.285695 | 0.511477 | N | -1.790320 | -1.432611 | -1.658411 | C | -3.228462 | -1.104576 | 1.641214 |
| | H | 3.943551 | -0.017692 | 1.553580 | C | 1.491754 | -2.240869 | 2.362653 | H | -2.078007 | -3.300045 | -0.758918 | C | -1.036375 | 2.613645 | -2.443169 |
| | C | 3.020003 | -0.362672 | 2.044661 | H | 1.786284 | -2.337978 | 3.416557 | C | -3.127816 | -1.844799 | 0.415402 | H | -1.485621 | 2.806291 | -3.429988 |
| | H | 3.943531 | -1.553508 | 0.014296 | H | -1.321957 | 2.576159 | 3.552322 | C | -2.662060 | -2.383409 | -0.924750 | H | -2.836802 | -2.781439 | -2.448298 |
| | H | 1.388655 | -3.209776 | -0.451276 | H | -1.913959 | 0.941993 | 3.079054 | H | -3.862003 | 0.498277 | 1.813725 | H | -3.117130 | -1.457466 | -1.295111 |
| | C | 3.020026 | -2.045374 | 0.358274 | H | 0.134945 | -3.893357 | 2.377650 | H | -2.689760 | 3.397602 | -1.217623 | H | -2.698309 | 3.546359 | -1.466422 |
| | H | 3.303757 | -1.019138 | 2.882051 | H | -0.618009 | -2.318119 | 2.821425 | H | -3.786463 | -2.585085 | 0.898761 | H | -2.817147 | 1.775606 | -1.560705 |
| | H | 3.303857 | -2.884153 | 1.012930 | H | -3.089457 | 2.733921 | 1.832265 | H | -1.824930 | -0.306769 | -3.424222 | H | -2.908838 | -3.689796 | -0.194546 |
| | | | | | H | -1.537463 | 3.362734 | 1.219999 | C | -2.501871 | -0.517679 | -2.583237 | H | -1.307915 | -3.892678 | -0.873744 |
| | | | | | H | 2.510808 | 0.861331 | 2.439784 | H | -3.727460 | 2.004379 | 0.479075 | H | 0.352559 | -0.117858 | -3.828540 |
| | | | | | H | 2.424334 | -0.322715 | 3.772066 | C | -2.898385 | 0.791843 | -1.926703 | H | -0.839876 | 1.007529 | -4.478461 |
| | | | | | H | -0.069625 | 0.055464 | 3.905509 | H | -3.728345 | -0.939422 | 0.270495 | H | -2.538615 | 0.024742 | -2.846042 |
| | | | | | H | 0.867958 | 1.550491 | 4.231170 | H | -3.579041 | 0.601693 | -1.089561 | H | -1.964286 | -1.087852 | -4.095375 |
| | | | | | H | -1.550585 | -4.233939 | 0.918952 | H | -3.537908 | -2.665134 | -1.531224 | H | -3.309588 | 3.354066 | 0.759622 |
| | | | | | H | -1.431907 | -3.410395 | -0.656417 | H | -3.452056 | 1.413437 | -2.649481 | H | -2.116909 | 2.847749 | 1.944256 |
| | | | | | H | -3.289914 | 1.060377 | -1.134145 | H | -3.396753 | -0.999707 | -3.008497 | H | -1.697534 | -2.150544 | 2.714420 |
| | | | | | H | -4.157021 | 1.790100 | 0.243256 | | | | | H | -2.902934 | -3.203393 | 1.969115 |
| | | | | | H | -3.606463 | -2.940117 | 0.449233 | | | | | H | -4.158788 | 1.404333 | 1.905286 |
| | | | | | H | -2.755933 | -2.242275 | 1.860717 | | | | | H | -3.816375 | 0.960661 | 0.226885 |
| | | | | | H | -3.697565 | -0.263392 | 1.613600 | | | | | H | -3.801936 | -1.196908 | 0.710493 |
| | | | | | H | -4.612103 | -0.660061 | 0.124704 | | | | | H | -3.963637 | -1.085526 | 2.463121 |
| | | | | | H | 2.220898 | -2.778259 | 1.740796 | | | | | H | -0.358898 | 3.460909 | -2.249363 |
| | | | | | | | | | | | | | H | -0.588443 | -2.103124 | -2.569011 |
| | | | | | | | | | | | | | H | 0.729612 | 1.681822 | -2.649621 |
| | | | | | | | | | | | | | H | -0.941034 | 3.261251 | 0.096115 |
| | | | | | | | | | | | | | H | -0.652123 | -3.056526 | 0.989601 |
| | | | | | | | | | | | | | H | -0.671766 | 1.747573 | 2.622674 |
| | | | | | | | | | | | | | H | 1.048759 | 3.225834 | -0.146473 |
| | | | | | | | | | | | | | H | 0.513502 | -2.074804 | 2.597037 |
| | | | | | | | | | | | | | H | 0.545784 | -3.086961 | -0.946070 |
| | | | | | | | | | | | | | H | 2.189764 | 0.232621 | -2.547955 |
| | | | | | | | | | | | | | H | -2.177409 | 0.343785 | 2.546072 |
| 2 | O | -2.256205 | 1.090762 | -1.089243 | O | -1.550165 | 0.076371 | 2.138520 | N | 1.832825 | 0.088842 | 2.032797 | N | -0.896612 | -0.952336 | 2.645689 |
| | O | -1.528285 | 1.463426 | 1.465514 | O | 1.036077 | -0.978682 | 2.417180 | N | 1.643322 | 2.230170 | 0.007179 | N | -1.780159 | 1.671302 | 1.447369 |
| (S=1) | O | -1.528288 | -1.463425 | -1.465515 | O | -1.086881 | 2.410266 | 0.735226 | N | 2.114337 | -1.910876 | -0.005547 | N | -1.547172 | -2.424179 | 0.142267 |
| | O | -2.256196 | -1.090764 | 1.089246 | O | 2.631232 | -0.157288 | 0.258697 | N | 1.833851 | 0.101432 | -2.031657 | N | -1.383251 | 2.236505 | -1.414887 |
| | C | -3.036950 | 0.361378 | -2.044649 | O | 1.586450 | 2.366983 | -0.167093 | C | 2.285510 | -1.269961 | 2.400160 | N | -2.178812 | -0.531385 | -1.963197 |
| | C | -2.201020 | 2.546434 | 0.799823 | C | 2.401297 | -0.618351 | 2.635014 | C | 2.429348 | 2.354952 | 1.260068 | C | -2.781874 | 2.391008 | 0.639657 |
| | C | -2.201104 | -0.796273 | -2.547580 | C | -1.387792 | 2.494807 | 2.131794 | C | 2.938714 | -1.979157 | 1.225438 | C | -2.476174 | -2.358786 | 1.286202 |
| | C | -2.201093 | 0.796272 | 2.547582 | C | 3.189961 | -0.869068 | 1.374089 | C | 2.429738 | 2.362810 | -1.244663 | C | -2.143262 | 3.131945 | -0.516265 |
| | C | -2.201017 | -2.546434 | -0.799821 | C | -1.212928 | -0.854544 | 3.179828 | C | 2.939468 | -1.971433 | -1.236445 | C | -1.502885 | 0.162655 | 3.393146 |
| | C | -3.036939 | -0.361381 | 2.044655 | C | 0.235919 | -0.712255 | 3.570400 | C | 2.946344 | 1.035192 | -1.767837 | C | -2.461008 | 0.984113 | 2.559070 |
| | C | -3.036922 | -2.045132 | 0.358488 | C | -0.439475 | 3.575197 | 0.209643 | C | 2.286888 | -1.255040 | -2.407146 | C | -2.236679 | -2.830642 | -1.098393 |
| | C | -3.036930 | 2.045129 | -0.358481 | C | 3.418406 | 0.869601 | -0.357967 | C | 2.945566 | 1.024049 | 1.775214 | C | -2.133718 | 1.835213 | -2.624102 |
| | H | -3.320702 | 1.016867 | -2.882980 | C | 1.050068 | 3.543680 | 0.439921 | H | 1.399735 | -1.828229 | 2.734463 | C | -3.035588 | -1.692240 | -1.690486 |

| | | | |
|---|---|---|---|
| H | -3.960708 | 0.013696 | -1.555609 |
| H | -1.398610 | 3.204364 | 0.443011 |
| H | -2.820671 | 3.105397 | 1.515483 |
| H | -2.820826 | -1.511169 | -3.107440 |
| H | -1.398740 | -0.438618 | -3.205105 |
| H | -2.820814 | 1.511167 | 3.107445 |
| H | -1.398726 | 0.438620 | 3.205103 |
| H | -1.398604 | -3.204362 | -0.443013 |
| H | -2.820669 | -3.105399 | -1.515477 |
| H | -3.320686 | -1.016870 | 2.882988 |
| H | -3.960699 | -0.013701 | 1.555618 |
| H | -3.960655 | -1.555596 | 0.011438 |
| H | -3.320719 | -2.884381 | 1.012781 |
| H | -3.320731 | 2.884378 | -1.012774 |
| H | -3.960661 | 1.555592 | -0.011428 |
| Th | 0.000000 | 0.000004 | -0.000006 |
| H | 3.320825 | -1.010096 | -2.885257 |
| H | 3.320596 | -2.882096 | -1.019547 |
| C | 3.036992 | -0.356582 | -2.045412 |
| H | 1.398815 | 0.446090 | -3.204068 |
| C | 3.036880 | -2.044363 | -0.363279 |
| O | 2.256202 | -1.088227 | -1.091774 |
| H | 1.398526 | -3.205391 | 0.435538 |
| C | 2.201152 | 0.802222 | -2.545685 |
| H | 3.960705 | -0.010015 | -1.555496 |
| C | 2.200965 | -2.548325 | 0.793872 |
| H | 3.960657 | -1.555706 | -0.015113 |
| H | 2.820887 | 1.518424 | -3.103858 |
| H | 2.820602 | -3.108977 | 1.508220 |
| O | 1.528295 | 1.466848 | -1.462091 |
| O | 1.528274 | -1.466850 | 1.462092 |
| C | 2.200987 | 2.548319 | -0.793866 |
| H | 2.820633 | 3.108967 | -1.508210 |
| C | 2.201129 | -0.802228 | 2.545691 |
| H | 2.820855 | -1.518434 | 3.103869 |
| O | 2.256201 | 1.088220 | 1.091781 |
| H | 1.398549 | 3.205390 | -0.435537 |
| H | 3.960668 | 1.555689 | 0.015132 |
| C | 3.036891 | 2.044352 | 0.363291 |
| H | 3.960694 | 0.009996 | 1.555514 |
| H | 1.398789 | -0.446091 | 3.204069 |
| C | 3.036980 | 0.356570 | 2.045424 |
| H | 3.320607 | 2.882083 | 1.019562 |
| H | 3.320812 | 1.010081 | 2.885271 |

| | | | |
|---|---|---|---|
| C | 2.977096 | 2.226121 | 0.124221 |
| C | -2.223364 | 1.295830 | 2.501759 |
| H | -2.434103 | 1.305637 | 3.579992 |
| H | 2.822825 | -1.224877 | 3.453930 |
| H | 2.441533 | 0.447430 | 2.918578 |
| H | -1.962563 | 3.413261 | 2.332931 |
| H | -0.451486 | 2.513165 | 2.712298 |
| H | 4.234357 | -0.565822 | 1.530754 |
| H | 3.171885 | -1.936672 | 1.117367 |
| H | -1.408679 | -1.853625 | 2.768324 |
| H | -1.864556 | -0.700802 | 4.050701 |
| H | 0.445195 | 0.311646 | 3.925386 |
| H | 0.486365 | -1.423986 | 4.373991 |
| H | -0.872133 | 4.480363 | 0.660814 |
| H | -0.655172 | 3.583163 | -0.863531 |
| H | 3.254831 | 0.769472 | -1.439122 |
| H | 4.485778 | 0.713087 | -0.149900 |
| H | 1.511184 | 4.440495 | -0.005911 |
| H | 1.290357 | 3.529495 | 1.516233 |
| H | 3.128258 | 2.323499 | 1.213051 |
| H | 3.551985 | 3.018784 | -0.382155 |
| H | -3.176923 | 1.314439 | 1.955845 |
| Th | -0.032182 | -0.023406 | -0.027633 |
| O | -0.744083 | 1.474027 | -2.061901 |
| O | 1.241827 | -0.481326 | -2.436697 |
| O | -2.674174 | 0.148203 | -0.615002 |
| O | 1.127361 | -2.371051 | -0.356580 |
| O | -1.586457 | -2.344545 | 0.202040 |
| C | 1.430552 | -1.874755 | -2.716530 |
| C | -2.949789 | 0.459414 | -1.986640 |
| C | 1.977601 | -2.574888 | -1.497258 |
| C | 0.217770 | 1.561444 | -3.127079 |
| C | 0.653084 | 0.184843 | -3.560454 |
| C | -3.485992 | -0.906032 | -0.079729 |
| C | 0.493250 | -3.505666 | 0.250889 |
| C | -2.901252 | -2.266368 | -0.363278 |
| C | -0.952759 | -3.574262 | -0.164688 |
| C | -2.138500 | 1.674744 | -2.363842 |
| H | -2.266567 | 1.888227 | -3.433907 |
| H | 2.141919 | -1.989822 | -3.550873 |
| H | 0.454500 | -2.300894 | -3.003369 |
| H | -4.020522 | 0.692941 | -2.104071 |
| H | -2.684350 | -0.402135 | -2.619217 |
| H | 2.070818 | -3.648600 | -1.711315 |
| H | 2.969546 | -2.183360 | -1.236220 |
| H | 1.065918 | 2.131480 | -2.724410 |
| H | -0.204922 | 2.115208 | -3.976176 |
| H | -0.213533 | -0.404059 | -3.905266 |
| H | 1.390304 | 0.257192 | -4.376576 |
| H | -4.502466 | -0.842436 | -0.494911 |
| H | -3.539447 | -0.729124 | 0.999462 |
| H | 0.583949 | -3.359662 | 1.335648 |
| H | 1.016970 | -4.431849 | -0.023050 |

| | | | |
|---|---|---|---|
| H | 2.999921 | -1.248295 | 3.240911 |
| H | 1.767715 | 2.800575 | 2.017574 |
| H | 3.275584 | 3.048603 | 1.128428 |
| H | 3.906102 | -1.511753 | 1.004661 |
| H | 3.166603 | -3.017204 | 1.511895 |
| H | 3.275822 | 3.055765 | -1.108465 |
| H | 1.768277 | 2.812989 | -1.999622 |
| H | 3.167671 | -3.007661 | -1.529171 |
| H | 3.906659 | -1.505283 | -1.012187 |
| H | 3.549779 | 1.210071 | -2.674044 |
| H | 3.614782 | 0.576164 | -1.030773 |
| H | 3.001781 | -1.228112 | -3.247334 |
| H | 1.401369 | -1.811330 | -2.745398 |
| H | 3.548651 | 1.193239 | 2.682732 |
| H | 3.614245 | 0.569498 | 1.035598 |
| Th | 0.000023 | 0.000145 | -0.000184 |
| H | 1.319875 | 0.458125 | 2.835225 |
| H | 1.509034 | -2.731458 | -0.008275 |
| H | 1.321201 | 0.475580 | -2.832019 |
| H | 0.997341 | 3.017321 | 0.009567 |
| H | -1.401828 | 1.811435 | 2.745800 |
| H | -1.509083 | 2.730689 | 0.008060 |
| H | -1.322361 | -0.475548 | 2.833405 |
| C | -2.287185 | 1.255042 | 2.407277 |
| N | -2.114702 | 1.910369 | 0.005452 |
| N | -1.834006 | -0.101539 | 2.032326 |
| H | -1.399997 | 1.828231 | -2.734691 |
| H | -3.167858 | 3.007537 | 1.528876 |
| C | -2.939711 | 1.971226 | 1.236378 |
| H | -3.002354 | 1.228235 | 3.247240 |
| C | -2.285504 | 1.269677 | -2.400142 |
| C | -2.939025 | 1.978743 | -1.225502 |
| N | -1.832331 | -0.088959 | -2.032733 |
| H | -1.319835 | -0.458103 | -2.835519 |
| H | -3.167068 | 3.016789 | -1.511870 |
| H | -0.996344 | -3.016544 | -0.008838 |
| N | -1.643194 | -2.230092 | -0.006648 |
| H | -1.768836 | -2.813071 | 2.000196 |
| C | -2.946548 | -1.035071 | 1.767826 |
| C | -2.429961 | -2.362822 | 1.244976 |
| H | -3.906949 | 1.505078 | 1.012317 |
| H | -3.000040 | 1.247671 | -3.240783 |
| H | -3.550499 | -1.209877 | 2.673703 |
| H | -1.766972 | -2.800634 | -2.017135 |
| C | -2.428825 | -2.355106 | -1.259759 |
| H | -3.906344 | 1.511115 | -1.004889 |
| C | -2.945011 | -1.024186 | -1.774880 |
| H | -3.614537 | -0.575932 | 1.030393 |
| H | -3.613692 | -0.569616 | -1.035240 |
| H | -3.276057 | -3.055670 | 1.108410 |
| H | -3.548163 | -1.193495 | -2.682331 |
| H | -3.274990 | -3.048846 | -1.128242 |

| | | | |
|---|---|---|---|
| C | -2.995404 | 0.615276 | -2.392397 |
| C | -1.727905 | -2.173108 | 2.593086 |
| H | -2.450986 | -2.198471 | 3.425155 |
| H | -3.354225 | 3.104715 | 1.257327 |
| H | -3.494180 | 1.643515 | 0.268271 |
| H | -3.080112 | -3.281206 | 1.358399 |
| H | -3.165018 | -1.524592 | 1.115566 |
| H | -2.920145 | 3.684692 | -1.067885 |
| H | -1.463747 | 3.902360 | -0.117538 |
| H | -0.689893 | 0.814540 | 3.752578 |
| H | -2.029244 | -0.197079 | 4.293545 |
| H | -3.239661 | 0.342350 | 2.127178 |
| H | -2.976274 | 1.708682 | 3.212884 |
| H | -2.902355 | -3.692153 | -0.923445 |
| H | -1.475244 | -3.165180 | -1.819021 |
| H | -1.389352 | 1.604159 | -3.399021 |
| H | -2.759504 | 2.664042 | -2.993766 |
| H | -3.554326 | -2.041146 | -2.599152 |
| H | -3.819441 | -1.373730 | -0.989490 |
| H | -3.745987 | 0.802876 | -1.612665 |
| H | -3.554158 | 0.386102 | -3.315835 |
| H | -1.067391 | -3.042060 | 2.747465 |
| Th | -0.082527 | 0.034953 | -0.165245 |
| N | 1.138924 | -1.880968 | -1.951975 |
| N | 2.286209 | 0.791190 | -1.653139 |
| N | 1.557627 | -2.229262 | 0.971364 |
| N | 1.188116 | 2.716075 | 0.208092 |
| N | 1.986406 | 0.508465 | 1.938746 |
| C | 3.082961 | 1.948058 | -1.265008 |
| C | 2.351705 | -2.933672 | -0.051952 |
| C | 2.183925 | 3.091977 | -0.822766 |
| C | 2.171199 | -1.325685 | -2.862568 |
| C | 3.080326 | -0.295782 | -2.216721 |
| C | 2.383757 | -1.912923 | 2.152438 |
| C | 1.660643 | 2.928587 | 1.588349 |
| C | 3.011111 | -0.540555 | 2.050476 |
| C | 2.602282 | 1.840210 | 2.054887 |
| C | 1.551769 | -3.149203 | -1.319444 |
| H | 2.140086 | -3.769198 | -2.015391 |
| H | 3.715579 | 2.309177 | -2.095283 |
| H | 3.774528 | 1.643945 | -0.467731 |
| H | 2.708680 | -3.907449 | 0.323023 |
| H | 3.245959 | -2.336003 | -0.263997 |
| H | 2.803782 | 3.939004 | -0.484717 |
| H | 1.627280 | 3.447825 | -1.703411 |
| H | 1.639659 | -0.837970 | -3.691602 |
| H | 2.781395 | -2.134237 | -3.296609 |
| H | 3.696781 | -0.735409 | -1.418428 |
| H | 3.784954 | 0.068459 | -2.985282 |
| H | 3.173921 | -2.666472 | 2.302116 |
| H | 1.754621 | -1.962296 | 3.053795 |
| H | 0.779199 | 2.953079 | 2.247999 |
| H | 2.158148 | 3.907128 | 1.696520 |

| | | | | |
|---|---|---|---|---|
| | | H -3.542203 -3.043849 0.083503<br>H -2.825750 -2.448841 -1.447639<br>H -1.036977 -3.703675 -1.257069<br>H -1.456241 -4.417967 0.334567<br>H -2.471899 2.548996 -1.787441 | | H 3.665285 -0.380339 2.924838<br>H 3.652441 -0.477290 1.159211<br>H 3.517271 1.845889 1.452643<br>H 2.920223 2.051015 3.089498<br>H 0.646570 -3.729728 -1.085147<br>H 1.590166 1.070876 -2.359929<br>H 0.343084 -2.097643 -2.550415<br>H 0.845046 -2.886971 1.285238<br>H 0.408992 3.359151 0.089623<br>H -0.056194 -1.208159 3.157096<br>H -0.898563 -3.184338 0.353215<br>H -1.190897 2.389331 1.879972<br>H -0.575370 2.749139 -1.765126<br>H -1.564331 -0.751535 -2.754830<br>H 1.360336 0.406324 2.739065 |
| 1<br>(S=$^{1}/_{2}$) | O -2.433437 1.079302 -1.066243<br>O -1.673498 1.442515 1.460364<br>O -1.673501 -1.442505 -1.460372<br>O -2.433372 -1.079340 1.066282<br>C -3.197863 0.367681 -2.030815<br>C -2.344930 2.522985 0.808434<br>C -2.345249 -0.777682 -2.532725<br>C -2.345207 0.777679 2.532732<br>C -2.344896 -2.522989 -0.808428<br>C -3.197804 -0.367706 2.030843<br>C -3.197693 -2.035194 0.342868<br>C -3.197749 2.035170 -0.342838<br>H -3.470578 1.032263 -2.869140<br>H -4.131626 0.011707 -1.562214<br>H -1.536583 3.164040 0.430868<br>H -2.949417 3.087969 1.534401<br>H -2.950027 -1.496799 -3.106097<br>H -1.537105 -0.392572 -3.169540<br>H -2.949995 1.496782 3.106111<br>H -1.537042 0.392594 3.169534<br>H -1.536528 -3.164033 -0.430890<br>H -2.949396 -3.087979 -1.534381<br>H -3.470512 -1.032274 2.869181<br>H -4.131570 -0.011750 1.562236<br>H -4.131443 -1.562323 -0.007438<br>H -3.470406 -2.881594 0.997134<br>H -3.470468 2.881557 -0.997118<br>H -4.131495 1.562306 0.007488<br>Th -0.000000 0.000018 -0.000020<br>H 3.470418 -0.996520 -2.881786<br>H 3.470575 -2.868932 -1.032866<br>C 3.197716 -0.342427 -2.035248<br>H 1.536540 0.431566 -3.163913<br>C 3.197844 -2.030750 -0.368111<br>O 2.433407 -1.066040 -1.079534<br>H 1.537092 -3.169652 0.391890<br>C 2.344917 0.808970 -2.522801<br>H 4.131472 0.007777 -1.562313 | O 1.584463 0.914115 1.879626<br>O -1.193617 0.990376 2.248120<br>O 1.973274 -1.643649 0.918300<br>O -2.481234 -0.757354 0.486567<br>O -0.550015 -2.732871 0.341814<br>C -2.299706 0.205671 2.715105<br>C 2.325434 -1.357390 2.281857<br>C -3.206111 -0.130758 1.562328<br>C 0.961436 1.844293 2.784551<br>C -0.311340 1.268472 3.341445<br>C 1.792197 -3.044631 0.677666<br>C -2.829415 -2.105072 0.142240<br>C 0.413997 -3.505923 1.066173<br>C -1.868761 -3.071988 0.775858<br>C 2.665880 0.103120 2.384747<br>H 2.860998 0.352522 3.437808<br>H -2.868605 0.781956 3.466796<br>H -1.898120 -0.712495 3.186143<br>H 3.205469 -1.957798 2.568069<br>H 1.478079 -1.609037 2.947799<br>H -3.996005 -0.806165 1.922987<br>H -3.675487 0.771451 1.147849<br>H 0.765994 2.749159 2.194857<br>H 1.655143 2.092314 3.599869<br>H -0.112274 0.326789 3.887607<br>H -0.790460 1.988637 4.027848<br>H 2.547943 -3.616086 1.238650<br>H 1.963546 -3.195293 -0.392910<br>H -2.781322 -2.157159 -0.953181<br>H -3.857880 -2.324012 0.462706<br>H 0.295055 -4.574675 0.812544<br>H 0.229503 -3.370517 2.147988<br>H -1.907843 -3.005066 1.879413<br>H -2.106352 -4.105889 0.470126<br>H 3.555861 0.344593 1.786471<br>Th 0.010930 0.011882 -0.015200<br>O 1.120061 -1.477167 -1.860152<br>O -1.482188 -0.463983 -2.284675 | N -2.097418 -1.442856 1.300071<br>N -1.846722 -1.469222 -1.581328<br>N -2.011356 1.431843 1.308278<br>N -1.710208 1.404309 -1.439048<br>C -2.632052 -0.681405 2.440726<br>C -2.557767 -2.533147 -0.854747<br>C -3.081564 0.704562 2.019756<br>C -2.695530 -0.604441 -2.424053<br>C -2.515453 2.602680 0.562819<br>C -2.975198 0.744988 -1.785345<br>C -1.869270 2.722519 -0.817533<br>C -3.147748 -2.015907 0.443514<br>H -1.818968 -0.590702 3.174598<br>H -3.476250 -1.207918 2.920619<br>H -1.824749 -3.322505 -0.632634<br>H -3.354630 -2.982627 -1.471319<br>H -3.945464 0.637471 1.343986<br>H -3.427905 1.256038 2.910344<br>H -3.646689 -1.104361 -2.669417<br>H -2.157067 -0.436580 -3.369027<br>H -2.366207 3.540124 1.119445<br>H -3.601746 2.494788 0.446977<br>H -3.577543 1.362427 -2.474946<br>H -3.564786 0.619689 -0.868371<br>H -2.472634 3.406489 -1.439488<br>H -0.866789 3.166819 -0.733991<br>H -3.694145 -2.824403 0.957621<br>H -3.885361 -1.231021 0.228768<br>Th -0.000024 -0.155558 -0.000149<br>H -1.540938 -2.212911 1.678149<br>H -1.340665 1.731727 2.022543<br>H -1.155579 1.489619 -2.300523<br>H -1.153408 -1.900259 -2.198066<br>H 1.819486 -0.587794 -3.174943<br>H 1.341070 1.734185 -2.021582<br>H 1.540832 -2.210768 -1.679609<br>C 2.632507 -0.678979 -2.441056<br>N 2.011330 1.433489 -1.307313 | N 0.089258 2.569826 -1.185248<br>N 1.160932 2.027050 1.480944<br>N 1.567017 0.330847 -2.294275<br>N 1.420377 -0.736716 2.353773<br>N 2.514764 -1.465886 -0.215844<br>C 2.405781 1.555538 2.117884<br>C 2.123350 1.695183 -2.347628<br>C 2.123091 0.378434 3.019884<br>C 0.268560 3.615735 -0.161191<br>C 1.419649 3.284411 0.757714<br>C 2.634221 -0.653708 -2.527360<br>C 2.291399 -1.907646 2.174357<br>C 3.408334 -0.953922 -1.266872<br>C 3.256149 -1.692855 1.035456<br>C 1.006233 2.710641 -2.340190<br>H 1.419814 3.730171 -2.389907<br>H 2.857613 2.361061 2.726497<br>H 3.115927 1.306897 1.316967<br>H 2.703033 1.831447 -3.279197<br>H 2.814238 1.840554 -1.507085<br>H 3.057096 0.033746 3.490803<br>H 1.473274 0.726382 3.848226<br>H -0.656755 3.692438 0.431906<br>H 0.426707 4.602347 -0.625517<br>H 2.342351 3.161087 0.175500<br>H 1.596736 4.114522 1.460784<br>H 3.323343 -0.308252 -3.315815<br>H 2.179264 -1.572940 -2.931491<br>H 1.655651 -2.788022 1.976177<br>H 2.843019 -2.136261 3.100089<br>H 4.219243 -1.669960 -1.489599<br>H 3.887410 -0.043260 -0.881978<br>H 3.879948 -0.810214 1.227521<br>H 3.943662 -2.550897 0.946461<br>H 0.414295 2.555935 -3.258434<br>Th -0.000266 0.000329 -0.006598<br>N -0.106680 -2.393900 -1.508310<br>N -1.142375 -2.209065 1.218986 |

| | | | | | | | | | | | | | | | | |
|---|---|---|---|---|---|---|---|---|---|---|---|---|---|---|---|---|
| | C | 2.345225 | -2.532907 | 0.777139 | O | 2.504528 | 0.700281 | -0.876441 | N | 2.097911 | -1.441355 | -1.301082 | N | -1.588085 | -0.029394 | -2.298701 |
| | H | 4.131598 | -1.562214 | -0.012030 | O | -1.958263 | 1.685340 | -0.529151 | H | 0.866358 | 3.165729 | 0.736852 | N | -1.397900 | 0.422014 | 2.437818 |
| | H | 2.949416 | 1.535044 | -3.087637 | O | 0.612276 | 2.747485 | -0.312509 | H | 3.428630 | 1.258836 | -2.908866 | N | -2.517151 | 1.487553 | -0.002752 |
| | H | 2.949999 | -3.106431 | 1.496137 | C | -2.162560 | 0.707329 | -2.746826 | C | 3.081818 | 0.706684 | -2.018883 | C | -2.381412 | -1.826454 | 1.922963 |
| | O | 1.673525 | 1.460691 | -1.442177 | C | 2.769177 | 0.268040 | -2.214900 | H | 3.476818 | -1.204973 | -2.921326 | C | -2.158468 | -1.373106 | -2.508232 |
| | O | 1.673470 | -1.460698 | 1.442184 | C | -2.868800 | 1.382420 | -1.597202 | C | 1.868973 | 2.721646 | 0.820002 | C | -2.091196 | -0.771543 | 2.963570 |
| | C | 2.345282 | 2.532892 | -0.777123 | C | 0.192631 | -2.091788 | -2.767886 | C | 2.515257 | 2.603525 | -0.560449 | C | -0.262239 | -3.564721 | -0.625421 |
| | H | 2.950077 | 3.106404 | -1.496113 | C | -0.741198 | -1.062350 | -3.351429 | N | 1.710162 | 1.402716 | 1.439980 | C | -1.403107 | -3.364769 | 0.343068 |
| | C | 2.344856 | -0.808986 | 2.522817 | C | 2.884287 | 2.057669 | -0.623968 | H | 1.155044 | 1.486836 | 2.301318 | C | -2.648852 | 0.984351 | -2.400483 |
| | H | 2.949335 | -1.535067 | 3.087666 | C | -1.742863 | 3.053681 | -0.168469 | H | 2.365900 | 3.541597 | -1.115981 | C | -2.272670 | 1.603933 | 2.421877 |
| | O | 2.433404 | 1.066027 | 1.079552 | C | 1.810947 | 3.034034 | -1.035443 | H | 1.153110 | -1.902283 | 2.196207 | C | -3.417217 | 1.124546 | -1.108558 |
| | H | 1.537151 | 3.169651 | -0.391892 | C | -0.462835 | 3.561261 | -0.779406 | N | 1.846590 | -1.470746 | 1.579994 | C | -3.248113 | 1.539297 | 1.273672 |
| | H | 4.131625 | 1.562173 | 0.012082 | C | 2.461305 | -1.205966 | -2.303797 | H | 1.824816 | -3.323146 | 0.629580 | C | -1.050729 | -2.388826 | -2.650093 |
| | C | 3.197871 | 2.030724 | 0.368145 | H | 2.591322 | -1.546735 | -3.340699 | C | 3.148019 | -2.015510 | -0.445099 | H | -1.474501 | -3.392296 | -2.812264 |
| | H | 4.131439 | -0.007827 | 1.562354 | H | -2.905962 | 0.423444 | -3.511663 | C | 2.557776 | -2.533987 | 0.852578 | H | -2.824971 | -2.703755 | 2.429986 |
| | H | 1.536476 | -0.431564 | 3.163913 | H | -1.403456 | 1.382035 | -3.182410 | H | 3.945381 | 0.639013 | -1.342743 | H | -3.100703 | -1.481917 | 1.166969 |
| | C | 3.197683 | 0.342396 | 2.035276 | H | 3.835179 | 0.427321 | -2.449236 | H | 2.472074 | 3.405077 | 1.442799 | H | -2.758191 | -1.389313 | -3.437010 |
| | H | 3.470601 | 2.868902 | 1.032905 | H | 2.132677 | 0.838906 | -2.912143 | H | 3.694284 | -2.823619 | -0.959965 | H | -2.833010 | -1.616246 | -1.676957 |
| | H | 3.470387 | 0.996481 | 2.881820 | H | -3.340875 | 2.305818 | -1.962310 | H | 2.156555 | -0.439542 | 3.368567 | H | -3.020655 | -0.492573 | 3.484208 |
| | | | | | H | -3.646498 | 0.730728 | -1.178308 | C | 2.695259 | -0.606680 | 2.423602 | H | -1.432258 | -1.219318 | 3.734934 |
| | | | | | H | -0.359287 | -2.832883 | -2.174342 | H | 3.601564 | 2.495656 | -0.444686 | H | 0.672063 | -3.707896 | -0.059431 |
| | | | | | H | 0.737875 | -2.611049 | -3.568026 | C | 2.975124 | 0.743302 | 1.786110 | H | -0.419268 | -4.484869 | -1.210760 |
| | | | | | H | -0.169079 | -0.270448 | -3.866729 | H | 3.885801 | -1.231024 | -0.229436 | H | -2.334194 | -3.174758 | -0.207040 |
| | | | | | H | -1.437338 | -1.538057 | -4.062425 | H | 3.565088 | 0.618874 | 0.869250 | H | -1.565681 | -4.280093 | 0.935026 |
| | | | | | H | 3.819705 | 2.285854 | -1.156808 | H | 3.354533 | -2.984011 | 1.468887 | H | -3.342978 | 0.746158 | -3.223498 |
| | | | | | H | 3.068168 | 2.124093 | 0.452799 | H | 3.577120 | 1.360202 | 2.476495 | H | -2.189254 | 1.944451 | -2.686515 |
| | | | | | H | -1.690704 | 3.069388 | 0.928219 | H | 3.646310 | -1.106885 | 2.668788 | H | -1.639484 | 2.504175 | 2.335843 |
| | | | | | H | -2.592604 | 3.671388 | -0.491783 | | | | | H | -2.815095 | 1.707308 | 3.375040 |
| | | | | | H | 2.142774 | 4.061547 | -0.807059 | | | | | H | -4.223671 | 1.868773 | -1.232924 |
| | | | | | H | 1.591923 | 2.952525 | -2.114209 | | | | | H | -3.901780 | 0.174483 | -0.845017 |
| | | | | | H | -0.504189 | 3.490081 | -1.880928 | | | | | H | -3.866296 | 0.635350 | 1.350116 |
| | | | | | H | -0.289121 | 4.610955 | -0.488837 | | | | | H | -3.939786 | 2.398028 | 1.307827 |
| | | | | | H | 3.136059 | -1.778835 | -1.652418 | | | | | H | -0.478124 | -2.127855 | -3.556532 |
| | | | | | | | | | | | | | H | -0.505040 | -2.539381 | 1.961041 |
| | | | | | | | | | | | | | H | 0.817762 | -2.475981 | -1.934452 |
| | | | | | | | | | | | | | H | -0.977619 | 0.131777 | -3.145106 |
| | | | | | | | | | | | | | H | -0.677377 | 0.641229 | 3.161117 |
| | | | | | | | | | | | | | H | -0.844095 | 2.706735 | -1.574791 |
| | | | | | | | | | | | | | H | 0.948585 | 0.265325 | -3.148075 |
| | | | | | | | | | | | | | H | 0.531860 | 2.262061 | 2.265768 |
| | | | | | | | | | | | | | H | 0.707404 | -1.044940 | 3.050903 |
| | | | | | | | | | | | | | H | 2.211855 | -2.392533 | -0.525329 |
| | | | | | | | | | | | | | H | -2.215123 | 2.448071 | -0.182438 |
| 1 (S=$^{3}/_{2}$) | O | -2.389433 | 1.548474 | -0.017064 | O | 0.445342 | -1.385384 | 2.116475 | N | 2.129874 | -1.389791 | -1.328842 | N | 0.303049 | 2.547772 | -1.214808 |
| | O | -1.660942 | 0.022687 | 2.082564 | O | -0.101761 | 1.356800 | 2.351838 | N | 1.896602 | -1.497264 | 1.533626 | N | 1.384610 | 1.950888 | 1.411748 |
| | O | -1.660943 | -0.022687 | -2.082563 | O | -1.561005 | -2.219713 | 0.426378 | N | 1.924926 | 1.484858 | -1.287896 | N | 1.620729 | 0.206736 | -2.286902 |
| | O | -2.389434 | -1.548474 | 0.017064 | O | -1.515269 | 2.191696 | 0.093192 | N | 1.657880 | 1.372710 | 1.474103 | N | 1.437639 | -0.783455 | 2.337488 |
| | C | -3.166081 | 1.682070 | -1.204286 | O | -3.218924 | -0.065652 | -0.671082 | C | 2.621378 | -0.587883 | -2.458336 | N | 2.414454 | -1.650008 | -0.227721 |
| | C | -2.332993 | 1.253318 | 2.347261 | C | -1.174497 | 2.295380 | 2.496849 | C | 2.636403 | -2.524574 | 0.785290 | C | 2.627292 | 1.385821 | 1.969616 |
| | C | -2.333062 | 1.201740 | -2.374458 | C | -1.634836 | -2.585968 | 1.806738 | C | 3.021218 | 0.805351 | -2.012871 | C | 2.303445 | 1.510720 | -2.285248 |
| | C | -2.333061 | -1.201740 | 2.374459 | C | -1.322710 | 3.069797 | 1.212200 | C | 2.714546 | -0.629118 | 2.399128 | C | 2.293520 | 0.267946 | 2.926705 |
| | C | -2.332995 | -1.253318 | -2.347261 | C | 0.938347 | -0.614241 | 3.224897 | C | 2.397811 | 2.648944 | -0.508166 | C | 0.510967 | 3.589085 | -0.191388 |
| | C | -3.166081 | -1.682069 | 1.204287 | C | 0.015111 | 0.541122 | 3.522085 | C | 2.947110 | 0.745298 | 1.794903 | C | 1.659180 | 3.221161 | 0.718891 |

| | | | | |
|---|---|---|---|---|
| | C -3.166215 -1.707616 -1.166909 | C -2.810942 -2.366801 -0.264265 | C 1.768469 2.709991 0.884476 | C 2.581723 -0.873220 -2.543789 |
| | C -3.166213 1.707616 1.166910 | C -2.769303 2.266197 -0.607658 | C 3.204617 -1.962226 -0.504202 | C 2.166761 -2.048343 2.171827 |
| | H -3.454027 2.736326 -1.356640 | C -3.766584 -1.214299 -0.046859 | H 1.797081 -0.516479 -3.182701 | C 3.343316 -1.249811 -1.296202 |
| | H -4.090801 1.091001 -1.093891 | C -3.729318 1.170114 -0.205135 | H 3.480150 -1.070068 -2.958322 | C 3.135247 -1.960743 1.017364 |
| | H -1.524363 1.973068 2.537917 | C -0.234867 -2.628122 2.366351 | H 1.923171 -3.327394 0.547753 | C 1.284141 2.623515 -2.324286 |
| | H -2.959414 1.162169 3.248815 | H -0.278698 -2.823112 3.447329 | H 3.446886 -2.964828 1.390585 | H 1.786975 3.602800 -2.332568 |
| | H -2.959698 1.090511 -3.273613 | H -0.945220 2.990417 3.323153 | H 3.884425 0.754827 -1.335214 | H 3.197258 2.159092 2.515150 |
| | H -1.524463 1.917035 -2.581316 | H -2.095924 1.729100 2.724663 | H 3.349872 1.386895 -2.890784 | H 3.251803 1.048332 1.128035 |
| | H -2.959696 -1.090510 3.273614 | H -2.091025 -3.586689 1.900815 | H 3.682883 -1.101164 2.631600 | H 2.955688 1.604969 -3.171389 |
| | H -1.524463 -1.917035 2.581316 | H -2.241781 -1.839312 2.350807 | H 2.172081 -0.504464 3.348974 | H 2.948298 1.583327 -1.397321 |
| | H -1.524366 -1.973068 -2.537917 | H -2.172611 3.761263 1.302663 | H 2.210817 3.597352 -1.033880 | H 3.215147 -0.160607 3.349040 |
| | H -2.959416 -1.162168 -3.248814 | H -0.414517 3.655739 1.012820 | H 3.488392 2.575158 -0.408704 | H 1.730464 0.699355 3.779225 |
| | H -3.454027 -2.736325 1.356641 | H 1.934498 -0.263343 2.924159 | H 3.533769 1.365880 2.495070 | H -0.407635 3.687708 0.407828 |
| | H -4.090800 -1.091000 1.093893 | H 1.033218 -1.254921 4.114132 | H 3.532810 0.661971 0.870667 | H 0.694766 4.571627 -0.655052 |
| | H -4.090693 -1.113904 -1.069434 | H -0.991419 0.170879 3.789976 | H 2.361904 3.396180 1.513699 | H 2.578814 3.100109 0.130675 |
| | H -3.454600 -2.764822 -1.296254 | H 0.419367 1.144820 4.353433 | H 0.752964 3.128081 0.829308 | H 1.850982 4.032504 1.439095 |
| | H -3.454598 2.764822 1.296255 | H -3.281517 -3.311639 0.050435 | H 3.760378 -2.745666 -1.046359 | H 3.286848 -0.592696 -3.343812 |
| | H -4.090692 1.113905 1.069435 | H -2.553965 -2.441289 -1.327249 | H 3.929379 -1.168016 -0.277404 | H 2.027443 -1.740406 -2.942088 |
| | Th -0.002652 -0.000001 -0.000001 | H -2.520524 2.185049 -1.674290 | Th 0.000051 -0.213165 -0.000213 | H 1.434948 -2.854766 1.995145 |
| | H 3.449879 1.282398 -2.753308 | H -3.226644 3.251098 -0.430037 | H 1.570552 -2.158142 -1.711555 | H 2.703348 -2.321014 3.094993 |
| | H 3.449820 -1.339811 -2.726074 | H -4.744780 -1.469205 -0.493920 | H 1.258753 1.794075 -2.000468 | H 4.066748 -2.051560 -1.525292 |
| | C 3.171579 1.166796 -1.691544 | H -3.911551 -1.021282 1.030219 | H 1.112843 1.417267 2.344175 | H 3.927819 -0.395085 -0.926792 |
| | H 1.530134 2.528499 -1.979182 | H -3.833010 1.137624 0.895030 | H 1.199929 -1.957389 2.128221 | H 3.863290 -1.158304 1.196099 |
| | C 3.171639 -1.202100 -1.666923 | H -4.719882 1.373881 -0.650648 | H -1.796296 -0.521230 3.181669 | H 3.714742 -2.894810 0.927490 |
| | O 2.401639 -0.015912 -1.509078 | H 0.356340 -3.417758 1.881235 | H -1.259132 1.789760 2.002965 | H 0.727027 2.532268 -3.271635 |
| | H 1.530597 -2.570193 -1.925941 | Th -0.000035 0.030524 -0.000006 | H -1.570652 -2.161689 1.708449 | Th -0.005605 0.003780 -0.005931 |
| | C 2.333455 2.349984 -1.252242 | O -0.445381 -1.383871 -2.117382 | C -2.620966 -0.592280 2.457682 | N -0.346832 -2.399575 -1.489261 |
| | H 4.102112 1.082675 -1.105009 | O 0.102151 1.358155 -2.350850 | N -1.925536 1.481996 1.289901 | N -1.329932 -2.109111 1.240071 |
| | C 2.333687 -2.375928 -1.202804 | O 1.560597 -2.219566 -0.427695 | N -2.129543 -1.392499 1.326919 | N -1.664407 0.053020 -2.272121 |
| | H 4.102246 -1.105688 -1.082398 | O 1.515701 2.191576 -0.092127 | H -0.753535 3.130067 -0.823381 | N -1.367211 0.534491 2.439596 |
| | H 2.956860 3.253990 -1.168098 | O 3.218503 -0.066633 0.671217 | H -3.350398 1.381606 2.892698 | N -2.430922 1.652657 -0.001749 |
| | H 2.957297 -3.277785 -1.099366 | C 1.174916 2.296746 -2.495693 | C -3.021620 0.801284 2.014032 | C -2.535611 -1.619999 1.933685 |
| | O 1.652039 2.096729 -0.021822 | C 1.634538 -2.584954 -1.808321 | H -3.479187 -1.075577 2.957525 | C -2.359717 -1.238616 -2.394734 |
| | O 1.652042 -2.096728 0.021822 | C 1.323762 3.070382 -1.210659 | C -1.768843 2.711681 -0.879804 | C -2.151372 -0.596692 2.974305 |
| | C 2.333683 2.375929 1.202804 | C -0.938516 -0.612043 -3.225298 | C -2.398530 2.647607 0.512567 | C -0.538182 -3.547561 -0.584354 |
| | H 2.957292 3.277787 1.099366 | C -0.015255 0.543469 -3.521790 | N -1.657622 1.375589 -1.472166 | C -1.652214 -3.274731 0.399778 |
| | C 2.333456 -2.349983 1.252242 | C 2.810470 -2.367462 0.262914 | H -1.113021 1.422403 -2.342295 | C -2.622099 1.159396 -2.394246 |
| | H 2.956863 -3.253988 1.168099 | C 2.769548 2.265443 0.609134 | H -2.211721 3.594947 1.040272 | C -2.146673 1.779965 2.420217 |
| | O 2.401636 0.015913 1.509080 | C 3.766298 -1.214957 0.046469 | H -1.199091 -1.953248 -2.130136 | C -3.370250 1.388445 -1.102886 |
| | H 1.530591 2.570194 1.925940 | C 3.729439 1.169344 0.206438 | N -1.896722 -1.494438 -1.535534 | C -3.137664 1.784218 1.282729 |
| | H 4.102242 1.105691 1.082400 | C 0.234691 -2.626509 -2.368243 | H -1.923917 -3.326758 -0.553820 | C -1.353090 -2.347871 -2.577158 |
| | C 3.171635 1.202103 1.666924 | H 0.278874 -2.820398 -3.449409 | C -3.204563 -1.963223 0.501377 | H -1.865757 -3.316741 -2.679473 |
| | H 4.102111 -1.082671 1.105012 | H 0.945334 2.992359 -3.321445 | C -2.636840 -2.523091 -0.789413 | H -3.065321 -2.451969 2.431650 |
| | H 1.530135 -2.528499 1.979181 | H 2.096195 1.730531 -2.724325 | H -3.884850 0.751290 1.336365 | H -3.214002 -1.207341 1.171679 |
| | C 3.171578 -1.166794 1.691545 | H 2.090578 -3.585693 -1.902954 | H -2.362326 3.398921 -1.507836 | H -3.033073 -1.232041 -3.270163 |
| | H 3.449815 1.339814 2.726076 | H 2.241691 -1.838028 -2.351831 | H -3.760324 -2.747595 1.042184 | H -2.983438 -1.400704 -1.503571 |
| | H 3.449877 -1.282395 2.753309 | H 2.174251 3.761174 -1.300883 | H -2.170759 -0.498971 -3.349562 | H -3.048040 -0.242221 3.505416 |
| | | H 0.416162 3.657201 -1.011040 | C -2.713990 -0.624994 -2.400311 | H -1.519450 -1.092626 3.740310 |
| | | H -1.934698 -0.261418 -2.924361 | H -3.489086 2.573862 0.412793 | H 0.397846 -3.725693 -0.031847 |
| | | H -1.033326 -1.252154 -4.114950 | C -2.946761 0.748513 -1.794177 | H -0.751375 -4.468923 -1.149800 |
| | | H 0.991151 0.173296 -3.790291 | H -3.929211 -1.168432 0.276307 | H -2.583511 -3.058231 -0.140821 |
| | | H -0.419670 1.147991 -4.352470 | H -3.532611 0.663864 -0.870173 | H -1.846105 -4.168103 1.014856 |
| | | H 3.280891 -3.312103 -0.052600 | H -3.447584 -2.961646 -1.395569 | H -3.336690 0.975158 -3.213624 |

| | | | | |
|---|---|---|---|---|
| | | H 2.553465 -2.442814 1.325829 | H -3.533355 1.370048 -2.493571 | H -2.069239 2.065276 -2.695451 |
| | | H 2.520470 2.184114 1.675684 | H -3.682187 -1.096611 -2.634189 | H -1.443294 2.624970 2.320045 |
| | | H 3.227303 3.250229 0.431893 | | H -2.667879 1.934324 3.378681 |
| | | H 4.744297 -1.470232 0.493762 | | H -4.092043 2.214285 -1.226488 |
| | | H 3.911665 -1.021290 -1.030438 | | H -3.955639 0.497791 -0.834007 |
| | | H 3.833833 1.137615 -0.893687 | | H -3.830542 0.937254 1.376562 |
| | | H 4.719755 1.372616 0.652751 | | H -3.753928 2.698675 1.312599 |
| | | H -0.356773 -3.416547 -1.884101 | | H -0.815377 -2.158983 -3.521550 |
| | | | | H -0.699036 -2.437307 1.982026 |
| | | | | H 0.547634 -2.537003 -1.963281 |
| | | | | H -1.040872 0.123762 -3.118509 |
| | | | | H -0.622995 0.696564 3.162399 |
| | | | | H -0.602714 2.731403 -1.648117 |
| | | | | H 0.982706 0.218553 -3.126552 |
| | | | | H 0.794895 2.172362 2.228228 |
| | | | | H 0.719686 -0.968581 3.073626 |
| | | | | H 1.988225 -2.526777 -0.530837 |
| | | | | H -2.021542 2.569813 -0.186414 |
| 0 | O 2.248350 0.972519 1.154791 | O 1.748600 -0.433324 1.936559 | N -1.738004 -1.302047 -1.493486 | N -0.278400 1.827019 -2.152847 |
| | O 1.105665 1.731180 -1.153073 | O -0.315140 1.460993 2.307392 | N -1.570495 1.611319 -1.482230 | N -1.358888 -0.934897 -2.299797 |
| (S=0) | O 2.013860 -1.690922 1.071086 | O 0.382746 -2.584504 0.848072 | N -2.283040 -1.380968 1.203307 | N -1.498866 2.410223 0.440901 |
| | O 2.340729 -0.650385 -1.344469 | O -2.386739 1.051547 0.444930 | N -2.026198 1.388640 1.372654 | N -1.461197 -2.488063 0.194966 |
| | C 3.219051 0.252579 1.905083 | O -2.236604 -1.717048 0.178876 | C -2.141125 -2.605393 -0.942624 | N -2.373431 -0.114219 1.679612 |
| | C 1.736751 2.738773 -0.345586 | C -1.682509 1.551643 2.722281 | C -2.422867 0.860036 -2.424990 | C -2.568126 -1.672401 -1.901693 |
| | C 2.649441 -1.114735 2.214078 | C 0.770578 -2.635594 2.227031 | C -2.882963 -2.435052 0.380189 | C -2.156884 2.805029 -0.812998 |
| | C 1.718725 1.405167 -2.410216 | C -2.548453 1.948440 1.556491 | C -2.272545 2.652808 -0.709180 | C -2.230580 -2.845752 -1.013998 |
| | C 2.807689 -2.433074 0.145294 | C 1.847153 0.642313 2.882422 | C -3.237801 -0.645373 2.035946 | C -0.586416 1.115599 -3.404398 |
| | C 2.795969 0.350489 -2.255570 | C 0.488759 1.022807 3.406156 | C -2.976519 2.093975 0.514875 | C -1.727156 0.140763 -3.228155 |
| | C 3.374406 -1.542279 -0.941345 | C -0.672695 -3.493924 0.526943 | C -2.637312 0.674986 2.493489 | C -2.470945 2.349614 1.535879 |
| | C 2.785323 2.157842 0.569961 | C -3.516950 0.278974 0.028258 | C -2.889618 -0.474846 -1.882437 | C -2.261802 -2.552213 1.423314 |
| | H 3.446796 0.788300 2.843463 | C -2.026990 -2.941964 0.885531 | H -1.222900 -3.186643 -0.785557 | C -3.251793 1.056666 1.540079 |
| | H 4.150183 0.190363 1.315391 | C -3.452260 -1.106596 0.610065 | H -2.780038 -3.165442 -1.650160 | C -3.162106 -1.348721 1.563780 |
| | H 0.933020 3.188361 0.250111 | C 1.990431 -1.772927 2.405681 | H -1.819027 0.672304 -3.329045 | C -1.130830 3.011142 -1.902994 |
| | H 2.167367 3.526872 -0.983334 | H 2.263499 -1.755809 3.471485 | H -3.294163 1.462681 -2.735111 | H -1.631663 3.333299 -2.831011 |
| | H 3.434957 -1.791183 2.588141 | H -1.777393 2.313898 3.517087 | H -3.928109 -2.156882 0.185136 | H -3.104858 -2.056066 -2.791642 |
| | H 1.839956 -1.030144 2.952580 | H -1.990890 0.571929 3.131233 | H -2.917148 -3.406336 0.902757 | H -3.222790 -0.951162 -1.391195 |
| | H 2.139945 2.314407 -2.869253 | H 1.020181 -3.675107 2.502264 | H -2.999259 3.193869 -1.339759 | H -2.707664 3.756401 -0.677038 |
| | H 0.896511 1.026991 -3.028079 | H -0.057598 -2.281673 2.865861 | H -1.514084 3.376631 -0.375970 | H -2.880122 2.028004 -1.090816 |
| | H 2.105359 -3.159841 -0.288428 | H -3.598470 1.951515 1.886414 | H -3.566240 -1.225553 2.917690 | H -3.154122 -3.387430 -0.748424 |
| | H 3.612957 -2.965377 0.676432 | H -2.286499 2.952187 1.195964 | H -4.140840 -0.463745 1.432058 | H -1.619573 -3.563894 -1.595582 |
| | H 3.009445 -0.103511 -3.239436 | H 2.309080 1.475884 2.337386 | H -3.474058 2.920679 1.054258 | H 0.311335 0.560077 -3.720594 |
| | H 3.734409 0.781905 -1.867809 | H 2.503535 0.350057 3.714849 | H -3.771922 1.397758 0.211177 | H -0.821534 1.825792 -4.215664 |
| | H 4.243806 -0.956703 -0.595596 | H -0.001388 0.158986 3.891281 | H -3.413007 1.282720 2.996003 | H -2.599481 0.662059 -2.810858 |
| | H 3.697301 -2.156965 -1.800047 | H 0.577880 1.837448 4.147043 | H -1.833157 0.483999 3.219062 | H -2.029717 -0.265129 -4.209635 |
| | H 3.035480 2.895641 1.353659 | H -0.513329 -4.447495 1.055448 | H -3.494106 -0.983262 -2.657153 | H -3.173959 3.200892 1.491605 |
| | H 3.716513 1.914083 0.030599 | H -0.601284 -3.669335 -0.551324 | H -3.535550 -0.338795 -1.006835 | H -1.931285 2.480061 2.488413 |
| | Th 0.022872 -0.354471 0.055610 | H -3.471168 0.250993 -1.068518 | Th 0.001073 -0.012805 0.169806 | H -1.573230 -2.606600 2.284127 |
| | H -3.187009 -0.175306 3.139491 | H -4.449612 0.775066 0.334385 | H -1.200355 -1.475926 -2.359457 | H -2.865810 -3.474853 1.456173 |
| | H -3.702754 -2.243307 1.704144 | H -2.808290 -3.670477 0.601951 | H -1.542163 -1.782101 1.799180 | H -4.005607 1.088951 2.350378 |
| | C -2.953964 0.302446 2.170843 | H -2.109786 -2.746107 1.969416 | H -1.327286 2.058875 1.742366 | H -3.797083 0.939857 0.593876 |
| | H -1.110069 0.995939 3.032342 | H -3.466375 -1.071567 1.714757 | H -0.875478 2.092971 -2.064945 | H -3.803247 -1.255224 0.677660 |
| | C -3.385676 -1.624747 0.845371 | H -4.313604 -1.706793 0.266714 | H 1.168656 3.181488 -0.734051 | H -3.834003 -1.479579 2.431214 |
| | O -2.429439 -0.662172 1.268261 | H 2.834590 -2.167264 1.821816 | H 1.512829 1.772935 1.818733 | H -0.475152 3.841963 -1.591258 |

| | | | | |
|---|---|---|---|---|
| | H -2.002312 -3.182460 0.291352<br>C -1.908458 1.381977 2.385076<br>H -3.890568 0.707141 1.750223<br>C -2.713429 -2.497969 -0.195957<br>H -4.274428 -1.102769 0.449728<br>H -2.365161 2.278700 2.833841<br>H -3.455891 -3.079261 -0.765053<br>O -1.235722 1.733421 1.170056<br>O -1.900931 -1.728121 -1.080666<br>C -1.851459 2.709980 0.320176<br>H -2.336779 3.491372 0.925085<br>C -2.493652 -1.178683 -2.256789<br>H -3.238049 -1.880362 -2.667489<br>O -2.222732 0.934547 -1.203381<br>H -1.032555 3.176679 -0.240652<br>H -3.777178 1.806029 -0.152333<br>C -2.832937 2.091293 -0.646570<br>H -4.080406 0.071553 -1.444453<br>H -1.656765 -1.073201 -2.959207<br>C -3.125569 0.170787 -1.989943<br>H -3.072111 2.822218 -1.440171<br>H -3.331987 0.686611 -2.944463 | Th 0.008076 -0.011154 -0.000561<br>O -0.061228 -1.797433 -1.940576<br>O -1.326115 0.706416 -2.304817<br>O 2.382947 -1.075103 -0.849280<br>O -0.359506 2.582231 -0.439899<br>O 2.263063 1.682461 -0.182274<br>C -1.041662 2.048489 -2.715177<br>C 2.322202 -1.456538 -2.229763<br>C -1.183492 2.986241 -1.546077<br>C -1.122701 -1.596246 -2.886270<br>C -1.117704 -0.183711 -3.404841<br>C 3.545452 -0.306780 -0.528840<br>C 0.693013 3.458282 -0.024163<br>C 3.383654 1.146351 -0.889828<br>C 2.006759 3.019264 -0.610894<br>C 1.161241 -2.396753 -2.408354<br>H 1.070518 -2.655804 -3.473884<br>H -1.751092 2.350390 -3.507183<br>H -0.015834 2.080605 -3.125726<br>H 3.254993 -1.975471 -2.510972<br>H 2.200578 -0.560636 -2.863672<br>H -0.900636 3.997942 -1.874514<br>H -2.218445 3.007074 -1.179809<br>H -2.050724 -1.816822 -2.342397<br>H -1.018415 -2.303261 -3.721985<br>H -0.150620 0.053972 -3.884618<br>H -1.922533 -0.046370 -4.149023<br>H 4.419028 -0.720842 -1.057407<br>H 3.695902 -0.420838 0.549787<br>H 0.710065 3.405859 1.072544<br>H 0.469553 4.491116 -0.329082<br>H 4.297418 1.700095 -0.607186<br>H 3.217189 1.278285 -1.973815<br>H 1.973220 3.044370 -1.715553<br>H 2.820502 3.683119 -0.268658<br>H 1.310243 -3.315744 -1.823602 | H 1.198011 1.490691 -2.352494<br>C 2.099491 2.623454 -0.907624<br>N 2.253985 1.364787 1.214088<br>N 1.728373 1.321152 -1.484153<br>H 1.832386 -0.508933 3.217004<br>H 2.839431 3.405619 0.957201<br>C 2.840527 2.444782 0.413865<br>H 2.723769 3.213964 -1.602713<br>C 2.640755 -0.689921 2.494137<br>C 3.220665 0.642279 2.044143<br>N 2.039691 -1.408231 1.368892<br>H 1.353304 -2.084557 1.727913<br>H 3.530563 1.223929 2.931454<br>H 0.900214 -2.054891 -2.086935<br>N 1.598183 -1.582564 -1.495591<br>H 1.855656 -0.636542 -3.335919<br>C 2.897265 0.516257 -1.868415<br>C 2.452622 -0.819896 -2.425498<br>H 3.893781 2.197238 0.221943<br>H 3.424602 -1.288664 2.994893<br>H 3.502782 1.041583 -2.630775<br>H 1.543317 -3.365908 -0.423032<br>C 2.300482 -2.632245 -0.737501<br>H 4.131215 0.481016 1.445664<br>C 2.997492 -2.087119 0.497018<br>H 3.534157 0.383120 -0.985962<br>H 3.778154 -1.370564 0.204114<br>H 3.333504 -1.410939 -2.730500<br>H 3.513178 -2.913087 1.020402<br>H 3.034024 -3.159867 -1.371903 | Th 0.001186 -0.000956 -0.000794<br>N 0.397316 0.864715 2.678853<br>N 1.207944 -1.842292 1.754612<br>N 1.691563 2.288496 0.480592<br>N 1.269021 -2.320526 -1.156149<br>N 2.399796 0.365710 -1.628871<br>C 2.348771 -2.509181 1.103777<br>C 2.379718 2.083772 1.762505<br>C 1.935918 -3.198149 -0.175764<br>C 0.608492 -0.293070 3.562368<br>C 1.658213 -1.230535 3.012270<br>C 2.653303 2.592236 -0.582316<br>C 2.126950 -1.977761 -2.296889<br>C 3.348887 1.349465 -1.088329<br>C 3.105408 -0.879253 -1.963993<br>C 1.372628 1.960233 2.880863<br>H 1.895572 1.843105 3.844406<br>H 2.792288 -3.269664 1.778185<br>H 3.103252 -1.732542 0.919125<br>H 3.045413 2.939084 1.988833<br>H 2.994649 1.177189 1.685539<br>H 2.811948 -3.697490 -0.622740<br>H 1.230669 -4.016462 0.070265<br>H -0.344527 -0.838445 3.658842<br>H 0.890206 0.030255 4.579013<br>H 2.581388 -0.673722 2.803504<br>H 1.905537 -2.002086 3.763335<br>H 3.411506 3.318662 -0.238423<br>H 2.119892 3.103896 -1.400048<br>H 1.475508 -1.661308 -3.130894<br>H 2.670868 -2.865251 -2.662409<br>H 4.106570 1.632644 -1.843057<br>H 3.884088 0.859321 -0.263519<br>H 3.714142 -1.162330 -1.095488<br>H 3.801724 -0.730086 -2.809954<br>H 0.811178 2.909038 2.936059<br>H 0.558665 -2.599027 2.014150<br>H -0.507657 1.259887 2.936619<br>H 1.126273 3.154519 0.603854<br>H 0.522364 -2.911597 -1.569184<br>H 0.666297 2.194535 -2.259719<br>H -0.868775 3.206979 0.679123<br>H -0.782965 -1.605544 -2.829606<br>H -0.759673 -3.239341 0.291229<br>H -2.026040 -0.096448 2.644767<br>H 2.068570 0.742791 -2.523050 |
| 0<br><br>(S=1) | O 2.248017 0.951291 1.206819<br>O 1.215112 1.726978 -1.162628<br>O 1.924169 -1.718190 1.087017<br>O 2.412241 -0.656443 -1.266720<br>C 3.170471 0.181538 1.962837<br>C 1.840650 2.709289 -0.326707<br>C 2.537618 -1.163291 2.249170 | O -1.357391 1.387484 1.768744<br>O -0.903990 -1.343725 2.217862<br>O 1.124094 2.305815 0.984381<br>O 1.214263 -2.249551 0.617042<br>O 2.734289 0.042323 0.551985<br>C 0.072389 -2.229446 2.760609<br>C 0.674983 2.582545 2.312588 | N -1.741249 -1.311581 -1.485848<br>N -1.569003 1.600422 -1.492360<br>N -2.288374 -1.369772 1.213819<br>N -2.022191 1.398731 1.366114<br>C -2.150532 -2.608314 -0.923352<br>C -2.417330 0.844974 -2.434778<br>C -2.895415 -2.423071 0.395646 | N -0.452497 2.175318 -1.754282<br>N -1.504879 -0.533766 -2.276992<br>N -1.564460 2.184164 0.996168<br>N -1.227768 -2.590371 -0.184405<br>N -2.348919 -0.555229 1.558268<br>C -2.515756 -1.584579 -2.070802<br>C -2.081696 3.041524 -0.084507 |

| | | | | |
|---|---|---|---|---|
| | C 1.867384 1.379013 -2.390322 | C 0.669387 -3.069165 1.659035 | C -2.273970 2.644512 -0.725841 | C -1.929837 -2.832498 -1.453511 |
| | C 2.721745 -2.492023 0.192065 | C -2.235511 0.598058 2.595576 | C -3.235673 -0.628237 2.049345 | C -1.186865 1.798602 -2.975717 |
| | C 2.914362 0.296410 -2.195152 | C -1.474943 -0.524348 3.244858 | C -2.975785 2.093432 0.502770 | C -2.174919 0.680132 -2.773933 |
| | C 3.373102 -1.624140 -0.867566 | C 2.543463 2.407243 0.817913 | C -2.630709 0.694463 2.494512 | C -2.648102 1.836956 1.925832 |
| | C 2.847062 2.098400 0.618508 | C 2.624660 -2.318590 0.352079 | C -2.889886 -0.484396 -1.884699 | C -2.042859 -2.921256 0.992076 |
| | H 3.412138 0.694363 2.910528 | C 3.260514 1.158777 1.262384 | H -1.234571 -3.190753 -0.757900 | C -3.325475 0.544341 1.557375 |
| | H 4.106701 0.075029 1.387292 | C 3.335388 -1.169003 1.007995 | H -2.789254 -3.172693 -1.627729 | C -3.033594 -1.835870 1.324240 |
| | H 1.030016 3.169823 0.250955 | C -0.832665 2.611303 2.303868 | H -1.808634 0.649636 -3.334271 | C -0.999204 3.367159 -1.083831 |
| | H 2.305722 3.494339 -0.942756 | H -1.210132 2.768464 3.323989 | H -3.285127 1.447591 -2.754517 | H -1.379135 4.090899 -1.822999 |
| | H 3.285410 -1.865322 2.652926 | H -0.402543 -2.891596 3.504891 | H -3.937131 -2.135908 0.195541 | H -3.003147 -1.847477 -3.027819 |
| | H 1.711955 -1.049065 2.963524 | H 0.853620 -1.617728 3.245771 | H -2.940962 -3.391205 0.923291 | H -3.292598 -1.155508 -1.425202 |
| | H 2.317666 2.276831 -2.843055 | H 1.055835 3.566961 2.632962 | H -3.001553 3.179641 -1.360529 | H -2.478231 3.982693 0.336180 |
| | H 1.058674 0.998929 -3.027783 | H 1.042151 1.795784 2.991572 | H -1.517251 3.372767 -0.397594 | H -2.922457 2.528175 -0.569551 |
| | H 2.002700 -3.180556 -0.277579 | H 1.456338 -3.711085 2.079894 | H -3.557923 -1.202491 2.937269 | H -2.723735 -3.588157 -1.330503 |
| | H 3.475391 -3.068763 0.751105 | H -0.099173 -3.706796 1.201593 | H -4.143186 -0.449606 1.451379 | H -1.209826 -3.290477 -2.159023 |
| | H 3.116585 -0.193158 -3.164795 | H -3.028362 0.214552 1.937389 | H -3.475271 2.923226 1.035665 | H -0.427940 1.493098 -3.722713 |
| | H 3.864410 0.700925 -1.805960 | H -2.717117 1.232930 3.352898 | H -3.769194 1.392015 0.206098 | H -1.695711 2.673708 -3.412368 |
| | H 4.275143 -1.106855 -0.496590 | H -0.659104 -0.133772 3.877358 | H -3.403719 1.307310 2.994985 | H -2.946510 0.949678 -2.040147 |
| | H 3.663619 -2.245977 -1.733340 | H -2.161061 -1.130174 3.861650 | H -1.824321 0.507681 3.218619 | H -2.691342 0.475142 -3.730101 |
| | H 3.113084 2.838010 1.395071 | H 2.922162 3.277319 1.376434 | H -3.493402 -0.997263 -2.657014 | H -3.388428 2.652111 1.994483 |
| | H 3.775003 1.803713 0.099681 | H 2.713843 2.576278 -0.249946 | H -3.537966 -0.339797 -1.012113 | H -2.216882 1.747774 2.939879 |
| | Th -0.022607 -0.358518 -0.034028 | H 2.739940 -2.274487 -0.739559 | Th 0.001016 -0.011420 0.166533 | H -1.352670 -3.086898 1.838413 |
| | H -3.084085 -0.047426 3.215183 | H 3.033926 -3.277495 0.701184 | H -1.203620 -1.496996 -2.349357 | H -2.566327 -3.881311 0.849977 |
| | H -3.742441 -2.130414 1.795026 | H 4.338143 1.261017 1.043106 | H -1.545517 -1.771497 1.806186 | H -4.141570 0.347876 2.278707 |
| | C -2.837378 0.383922 2.229093 | H 3.130202 0.980096 2.343962 | H -1.326506 2.075438 1.729459 | H -3.779537 0.593600 0.556642 |
| | H -0.945578 1.047949 3.025249 | H 3.242042 -1.217964 2.107768 | H -0.872416 2.081272 -2.074763 | H -3.745642 -1.695724 0.500808 |
| | C -3.394235 -1.529241 0.936678 | H 4.404133 -1.181191 0.729297 | H 1.179631 3.177792 -0.737765 | H -3.631124 -2.130387 2.204877 |
| | O -2.364419 -0.638831 1.353455 | H -1.198689 3.430053 1.666861 | H 1.515020 1.782782 1.817425 | H -0.173446 3.875020 -0.559802 |
| | H -2.110317 -3.154286 0.334244 | Th -0.003095 0.013585 -0.002873 | H 1.200881 1.487069 -2.352639 | Th 0.000133 0.015913 -0.010147 |
| | C -1.754940 1.431191 2.393365 | O 1.352069 1.363878 -1.789095 | C 2.108333 2.616041 -0.910593 | N 0.423158 0.281021 2.772859 |
| | H -3.760113 0.816120 1.805628 | O 0.898584 -1.367056 -2.185787 | N 2.254140 1.366440 1.216578 | N 1.452323 -1.979604 1.253330 |
| | C -2.807503 -2.437370 -0.123842 | O -1.131439 2.280413 -1.019755 | N 1.732761 1.314418 -1.485394 | N 1.619639 2.113250 0.888312 |
| | H -4.250834 -0.942892 0.561296 | O -1.201087 -2.272956 -0.575717 | H 1.821994 -0.505451 3.221259 | N 1.340137 -1.937619 -1.627680 |
| | H -2.177246 2.346013 2.839198 | O -2.719677 0.017921 -0.554207 | H 2.854438 3.401836 0.949376 | N 2.263660 0.766615 -1.575950 |
| | H -3.602587 -2.981068 -0.658541 | C -0.055875 -2.281674 -2.720488 | C 2.848588 2.438280 0.410775 | C 2.656526 -2.308185 0.471112 |
| | O -1.119481 1.744898 1.145321 | C -0.686108 2.541283 -2.352818 | H 2.733774 3.203036 -1.607935 | C 2.378948 1.660049 2.066552 |
| | O -2.000884 -1.709347 -1.049516 | C -0.651109 -3.105366 -1.606517 | C 2.633289 -0.687304 2.501929 | C 2.259051 -2.832429 -0.889232 |
| | C -1.738498 2.742447 0.314565 | C 2.231814 0.562984 -2.605004 | C 3.215808 0.644075 2.053057 | C 0.677809 -1.038789 3.374130 |
| | H -2.184432 3.536242 0.934631 | C 1.478723 -0.576687 -3.230515 | N 2.036495 -1.406197 1.374744 | C 1.805300 -1.746227 2.662980 |
| | C -2.621015 -1.148501 -2.209227 | C -2.552158 2.380966 -0.853109 | H 1.351457 -2.084453 1.731597 | C 2.519469 2.707496 -0.115186 |
| | H -3.408938 -1.825151 -2.578055 | C -2.613261 -2.339560 -0.318924 | H 3.521740 1.226787 2.940951 | C 1.994756 -1.317595 -2.790887 |
| | O -2.225198 0.947097 -1.160596 | C -3.263887 1.122643 -1.273347 | H 0.901148 -2.066228 -2.078216 | C 3.237112 1.644374 -0.910278 |
| | H -0.925121 3.188669 -0.270063 | C -3.322783 -1.199394 -0.991902 | N 1.599617 -1.588093 -1.492140 | C 2.975186 -0.268045 -2.338619 |
| | H -3.715241 1.923890 -0.098205 | C 0.820998 2.575894 -2.346151 | H 1.848865 -0.645535 -3.335251 | C 1.426720 1.286745 3.175486 |
| | C -2.767257 2.149497 -0.615868 | H 1.196410 2.715779 -3.369608 | C 2.899053 0.508280 -1.875175 | H 1.987096 0.951102 4.062848 |
| | H -4.130277 0.184545 -1.366054 | H 0.441917 -2.953794 -3.441019 | C 2.450221 -0.828313 -2.427613 | H 3.246492 -3.092987 0.981483 |
| | H -1.805013 -1.084603 -2.942125 | H -0.839071 -1.696639 -3.234356 | H 3.899948 2.181968 0.220051 | H 3.282096 -1.405723 0.407825 |
| | C -3.182636 0.229000 -1.930473 | H -1.071548 3.519811 -2.685709 | H 3.415102 -1.285930 3.006086 | H 3.042033 2.468379 2.429285 |
| | H -2.989525 2.872405 -1.421918 | H -1.053100 1.745969 -3.021844 | H 3.499807 1.032647 -2.642107 | H 3.018759 0.818049 1.772163 |
| | H -3.377846 0.757857 -2.880133 | H -1.435092 -3.757116 -2.018083 | H 1.549432 -3.367837 -0.414389 | H 3.157816 -3.050681 -1.487537 |
| | | H 0.119999 -3.733119 -1.139682 | C 2.304877 -2.632564 -0.729544 | H 1.739162 -3.795975 -0.734484 |
| | | H 3.028194 0.196028 -1.942271 | H 4.129168 0.481406 1.459397 | H -0.240322 -1.644054 3.300772 |
| | | H 2.705517 1.185434 -3.377677 | C 2.998151 -2.081968 0.504731 | H 0.903554 -0.944793 4.448695 |

| | | | | |
|---|---|---|---|---|
| | | H 0.669891 -0.207069 -3.884020 | H 3.541707 0.376423 -0.996798 | H 2.719870 -1.139274 2.700690 |
| | | H 2.173808 -1.199620 -3.821488 | H 3.775320 -1.361880 0.211400 | H 2.033134 -2.701258 3.166323 |
| | | H -2.934508 3.238492 -1.428505 | H 3.328591 -1.420683 -2.736853 | H 3.251543 3.380020 0.363006 |
| | | H -2.723873 2.572569 0.210730 | H 3.517571 -2.904702 1.029597 | H 1.929260 3.325205 -0.807265 |
| | | H -2.732779 -2.280585 0.771002 | H 3.041033 -3.159560 -1.361465 | H 1.230640 -0.841353 -3.427684 |
| | | H -3.019671 -3.302757 -0.660064 | | H 2.501286 -2.074607 -3.412699 |
| | | H -4.339910 1.218842 -1.039719 | | H 3.884917 2.137278 -1.658364 |
| | | H -3.146449 0.929272 -2.353690 | | H 3.885202 1.030724 -0.268170 |
| | | H -3.230640 -1.264801 -2.091020 | | H 3.755875 -0.711343 -1.705489 |
| | | H -4.391798 -1.204752 -0.712368 | | H 3.476319 0.179844 -3.215613 |
| | | H 1.183307 3.407137 -1.723511 | | H 0.876069 2.198061 3.478269 |
| | | | | H 0.892042 -2.856477 1.246076 |
| | | | | H -0.460227 0.617672 3.194921 |
| | | | | H 1.021567 2.879870 1.237805 |
| | | | | H 0.611741 -2.554432 -2.004767 |
| | | | | H 0.469135 2.490781 -2.140050 |
| | | | | H -0.931900 2.797853 1.555694 |
| | | | | H -0.884847 -0.853008 -3.031440 |
| | | | | H -0.469528 -3.313723 -0.155992 |
| | | | | H -1.988996 -0.597773 2.519880 |
| | | | | H 1.856303 1.378772 -2.329747 |
| 0 | O 2.657466 -0.982888 -0.840740 | O -0.700725 -1.755021 1.816631 | N -1.702846 -1.288070 -1.520622 | N -0.627803 2.220386 -1.662523 |
| | O 1.224117 -1.663262 1.299744 | O 1.592908 -0.229121 2.220593 | N -1.553582 1.635070 -1.457565 | N -1.624925 -0.502399 -2.214679 |
| (S=2) | O 1.935659 1.488582 -1.390702 | O -2.567490 0.080811 0.981552 | N -2.240204 -1.375222 1.190968 | N -1.636492 2.097009 1.121705 |
| | O 1.921192 0.980010 1.257963 | O 1.536658 2.047028 0.619893 | N -1.994459 1.380295 1.404233 | N -1.152614 -2.630877 -0.233526 |
| | C 3.622235 -0.252517 -1.574498 | O -1.166865 2.466438 0.514907 | C -2.136319 -2.580907 -0.962989 | N -2.310690 -0.682476 1.580823 |
| | C 2.084103 -2.654222 0.738104 | C 2.050482 1.013248 2.752475 | C -2.345470 0.888582 -2.454900 | C -2.566214 -1.616523 -2.024494 |
| | C 2.894183 0.888541 -2.258373 | C -2.668357 -0.437431 2.310615 | C -2.884123 -2.390909 0.350735 | C -2.202201 2.990904 0.097476 |
| | C 1.624469 -1.048463 2.527324 | C 2.538557 1.898191 1.632333 | C -2.317561 2.627205 -0.676933 | C -1.884046 -2.854405 -1.490119 |
| | C 2.344011 2.530153 -0.507370 | C 0.378277 -2.237132 2.643895 | C -3.159568 -0.673134 2.091211 | C -1.433759 1.862928 -2.844688 |
| | C 2.474503 0.185675 2.313912 | C 1.111556 -1.082482 3.266789 | C -2.987706 2.025715 0.545846 | C -2.367204 0.700764 -2.624378 |
| | C 2.832218 1.985681 0.819507 | C -3.250154 1.325421 0.784587 | C -2.558578 0.650873 2.543092 | C -2.684499 1.675573 2.060779 |
| | C 3.207237 -2.041866 -0.075478 | C 1.027416 3.358133 0.325874 | C -2.835320 -0.454071 -1.954805 | C -1.923090 -3.023231 0.954174 |
| | H 4.099045 -0.897531 -2.333754 | C -2.414584 2.505422 1.206234 | H -1.231938 -3.179593 -0.788129 | C -3.326322 0.377558 1.649401 |
| | H 4.409867 0.106032 -0.887593 | C -0.326857 3.537103 0.951538 | H -2.775972 -3.134348 -1.674950 | C -2.945689 -1.987917 1.347966 |
| | H 1.432930 -3.240583 0.076562 | C -2.039246 -1.808849 2.330175 | H -1.689155 0.716030 -3.325910 | C -1.159329 3.376921 -0.922308 |
| | H 2.488640 -3.300607 1.533517 | H -2.032509 -2.200252 3.357058 | H -3.200492 1.489307 -2.810439 | H -1.573681 4.127419 -1.614638 |
| | H 3.603398 1.645885 -2.627877 | H 2.879898 0.828559 3.457120 | H -3.914901 -2.065503 0.153811 | H -3.078009 -1.863154 -2.972512 |
| | H 2.297138 0.505939 -3.097336 | H 1.206755 1.489242 3.283110 | H -2.956553 -3.365929 0.863775 | H -3.339978 -1.263403 -1.330355 |
| | H 2.169238 -1.765244 3.163830 | H -3.731557 -0.521810 2.593422 | H -3.074213 3.126941 -1.306642 | H -2.603682 3.904464 0.569778 |
| | H 0.693494 -0.799171 3.052666 | H -2.144566 0.238807 3.006176 | H -1.601524 3.392302 -0.340824 | H -3.048941 2.482866 -0.384347 |
| | H 1.439734 3.140572 -0.368450 | H 2.807630 2.882581 2.041089 | H -3.427614 -1.276440 2.976263 | H -2.624572 -3.664107 -1.379357 |
| | H 3.124032 3.149288 -0.978894 | H 3.425848 1.460769 1.154106 | H -4.097110 -0.495768 1.543135 | H -1.161388 -3.230968 -2.239523 |
| | H 2.506116 0.769247 3.252714 | H 1.040892 -2.825505 1.992083 | H -3.534471 2.820611 1.085255 | H -0.716728 1.610959 -3.650708 |
| | H 3.508859 -0.081800 2.044102 | H -0.007301 -2.916002 3.417883 | H -3.736974 1.280214 0.244289 | H -2.001358 2.734812 -3.209455 |
| | H 3.835556 1.537207 0.725733 | H 0.442702 -0.493822 3.917895 | H -3.324983 1.243360 3.076226 | H -3.105718 0.913737 -1.838058 |
| | H 2.890087 2.796367 1.565877 | H 1.965539 -1.461971 3.855826 | H -1.727819 0.466275 3.238580 | H -2.933263 0.511779 -3.555188 |
| | H 3.638354 -2.811642 -0.740803 | H -4.200510 1.321335 1.340703 | H -3.396029 -0.954835 -2.766374 | H -3.448762 2.461676 2.182582 |
| | H 4.017354 -1.646625 0.562415 | H -3.474697 1.386453 -0.285367 | H -3.527785 -0.333581 -1.113483 | H -2.221701 1.556998 3.058778 |
| | Th -0.000104 -0.002185 -0.433167 | H 0.963589 3.433634 -0.768804 | Th -0.000198 -0.000358 0.168691 | H -1.202270 -3.183806 1.775411 |
| | H -4.100252 0.895444 -2.333051 | H 1.723460 4.130567 0.683751 | H -1.152798 -1.483281 -2.374314 | H -2.413384 -3.999162 0.799274 |
| | H -3.636163 2.812329 -0.745529 | H -2.947219 3.438208 0.948438 | H -1.495852 -1.839384 1.748312 | H -4.120761 0.120535 2.374720 |
| | C -3.623632 0.251480 -1.572772 | H -2.212262 2.491588 2.291064 | H -1.328244 2.089176 1.752306 | H -3.802610 0.454242 0.659592 |

| | | | | |
|---|---|---|---|---|
| | H -2.300686 -0.511361 -3.095044 | H -0.262698 3.504924 2.053793 | H -0.859600 2.166708 -1.999693 | H -3.697548 -1.863045 0.558033 |
| | C -3.205184 2.043925 -0.078531 | H -0.762184 4.501363 0.633350 | H 1.223749 3.178183 -0.794896 | H -3.493629 -2.327314 2.244544 |
| | O -2.657422 0.982205 -0.841343 | H -2.608544 -2.504134 1.695388 | H 1.496051 1.840793 1.751831 | H -0.320656 3.870396 -0.405680 |
| | H -1.429235 3.240824 0.068088 | Th -0.018017 0.001518 0.013414 | H 1.156806 1.475819 -2.378184 | Th -0.002805 0.009245 -0.016655 |
| | C -2.897419 -0.891777 -2.254890 | O -1.794126 0.601811 -1.815276 | C 2.130657 2.582353 -0.966569 | N 0.543879 0.224570 2.770852 |
| | H -4.411208 -0.104657 -0.884533 | O 0.856883 1.341737 -2.233364 | N 2.237998 1.379207 1.189407 | N 1.606481 -1.935831 1.153117 |
| | C -2.080395 2.657196 0.732046 | O -1.589331 -2.012722 -0.990206 | N 1.703231 1.286293 -1.521220 | N 1.606342 2.131916 0.886617 |
| | H -4.015067 1.651487 0.561404 | O 2.560582 -0.147563 -0.612880 | H 1.735446 -0.460481 3.237799 | N 1.392004 -1.851728 -1.712281 |
| | H -3.607690 -1.649197 -2.622210 | O 1.132795 -2.477021 -0.543149 | H 2.939904 3.374675 0.861899 | N 2.216830 0.881835 -1.613877 |
| | H -2.483197 3.306501 1.525956 | C 2.098835 0.883512 -2.760887 | C 2.876502 2.398837 0.349369 | C 2.799535 -2.192384 0.329255 |
| | O -1.938710 -1.491127 -1.386886 | C -2.042816 -1.750878 -2.319491 | H 2.769793 3.136148 -1.678755 | C 2.438841 1.641458 1.998471 |
| | O -1.221132 1.666774 1.295564 | C 3.087638 0.704810 -1.636246 | C 2.563751 -0.646901 2.539843 | C 2.377423 -2.715254 -1.023962 |
| | C -2.347505 -2.529978 -0.500538 | C -1.455789 1.726082 -2.653449 | C 3.163052 0.676105 2.083185 | C 0.842826 -1.105796 3.324805 |
| | H -3.128853 -3.149242 -0.969708 | C -0.104987 1.522575 -3.278830 | N 1.995796 -1.378927 1.404638 | C 1.973458 -1.759786 2.567368 |
| | C -1.621407 1.055584 2.524936 | C -1.079761 -3.337568 -0.796206 | H 1.326982 -2.083553 1.756303 | C 2.432062 2.804869 -0.130353 |
| | H -2.165260 1.774454 3.159884 | C 3.229075 -1.388115 -0.334481 | H 3.438350 1.279318 2.966083 | C 1.979676 -1.177729 -2.881148 |
| | O -1.920999 -0.976141 1.260959 | C 0.358566 -3.461664 -1.224279 | H 0.861799 -2.163927 -2.003578 | C 3.171514 1.804384 -0.985758 |
| | H -1.443807 -3.141207 -0.361152 | C 2.492286 -2.529640 -0.975389 | N 1.554938 -1.635895 -1.456212 | C 2.933865 -0.097955 -2.440356 |
| | H -3.836525 -1.532040 0.731573 | C -2.692378 -0.389737 -2.332826 | H 1.705437 -0.719832 -3.324726 | C 1.556691 1.229797 3.150358 |
| | C -2.833730 -1.981661 0.825487 | H -2.991675 -0.128417 -3.357426 | C 2.839827 0.452959 -1.945739 | H 2.168353 0.875213 3.994979 |
| | H -3.506990 0.089404 2.045446 | H 2.492921 1.626253 -3.476346 | C 2.354679 -0.890579 -2.448020 | H 3.445362 -2.952591 0.806379 |
| | H -0.690432 0.806915 3.050566 | H 1.921778 -0.074149 -3.282228 | H 3.910263 2.081401 0.155240 | H 3.379494 -1.259548 0.259134 |
| | C -2.472724 -0.178349 2.315262 | H -2.786560 -2.511335 -2.612489 | H 3.331911 -1.238145 3.071814 | H 3.127606 2.435318 2.342428 |
| | H -2.892040 -2.790542 1.573769 | H -1.187353 -1.780687 -3.014047 | H 3.406732 0.953410 -2.753182 | H 3.057549 0.808344 1.637169 |
| | H -2.504239 -0.759458 3.255579 | H 4.019097 0.279084 -2.035795 | H 1.590544 -3.391847 -0.337215 | H 3.261296 -2.883606 -1.659042 |
| | | H 3.315292 1.673739 -1.170577 | C 2.311818 -2.631970 -0.673949 | H 1.905766 -3.702907 -0.865774 |
| | | H -1.464241 2.616921 -2.008802 | H 4.096317 0.497274 1.528258 | H -0.060555 -1.732147 3.247858 |
| | | H -2.224405 1.871789 -3.426173 | C 2.985294 -2.032600 0.548032 | H 1.089222 -1.041251 4.397268 |
| | | H -0.097288 0.626797 -3.923908 | H 3.525317 0.333944 -1.098528 | H 2.877182 -1.137486 2.620366 |
| | | H 0.162020 2.411393 -3.878577 | H 3.739709 -1.292758 0.245318 | H 2.226985 -2.731482 3.024413 |
| | | H -1.699736 -4.061193 -1.348309 | H 3.212792 -1.491487 -2.795622 | H 3.146430 3.500672 0.341469 |
| | | H -1.172256 -3.548948 0.274184 | H 3.526599 -2.829917 1.089361 | H 1.781447 3.409118 -0.778923 |
| | | H 3.239933 -1.500094 0.758466 | H 3.065594 -3.137616 -1.302408 | H 1.173989 -0.717356 -3.476446 |
| | | H 4.269901 -1.351870 -0.687509 | | H 2.495310 -1.898589 -3.538335 |
| | | H 0.729555 -4.468873 -0.961308 | | H 3.747869 2.349639 -1.754884 |
| | | H 0.474823 -3.309778 -2.311519 | | H 3.890169 1.220415 -0.392236 |
| | | H 2.516091 -2.452882 -2.077322 | | H 3.763670 -0.522133 -1.857993 |
| | | H 2.943689 -3.489130 -0.664280 | | H 3.372140 0.395552 -3.326464 |
| | | H -3.589219 -0.386837 -1.695552 | | H 1.012867 2.125075 3.508649 |
| | | | | H 1.061702 -2.818610 1.111579 |
| | | | | H -0.325842 0.544742 3.233246 |
| | | | | H 0.996521 2.852413 1.303347 |
| | | | | H 0.680299 -2.495369 -2.076519 |
| | | | | H 0.264266 2.557977 -2.092683 |
| | | | | H -0.997002 2.689789 1.692710 |
| | | | | H -1.007992 -0.746958 -2.996492 |
| | | | | H -0.370597 -3.329489 -0.250465 |
| | | | | H -1.893003 -0.727329 2.516293 |
| | | | | H 1.718303 1.473719 -2.323473 |
